# Predicting electronic structure properties of transition metal complexes with neural networks†
†Electronic supplementary information (ESI) available. See DOI: 10.1039/c7sc01247k


**DOI:** 10.1039/c7sc01247k

**Published:** 2017-05-17

**Authors:** Jon Paul Janet, Heather J. Kulik

**Affiliations:** a Department of Chemical Engineering , Massachusetts Institute of Technology , Cambridge , MA 02139 , USA . Email: hjkulik@mit.edu ; Tel: +1-617-253-4584

## Abstract

High-throughput computational screening has emerged as a critical component of materials discovery. Direct density functional theory (DFT) simulation of inorganic materials and molecular transition metal complexes is often used to describe subtle trends in inorganic bonding and spin-state ordering, but these calculations are computationally costly and properties are sensitive to the exchange–correlation functional employed. To begin to overcome these challenges, we trained artificial neural networks (ANNs) to predict quantum-mechanically-derived properties, including spin-state ordering, sensitivity to Hartree–Fock exchange, and spin-state specific bond lengths in transition metal complexes. Our ANN is trained on a small set of inorganic-chemistry-appropriate empirical inputs that are both maximally transferable and do not require precise three-dimensional structural information for prediction. Using these descriptors, our ANN predicts spin-state splittings of single-site transition metal complexes (*i.e.*, Cr–Ni) at arbitrary amounts of Hartree–Fock exchange to within 3 kcal mol^–1^ accuracy of DFT calculations. Our exchange-sensitivity ANN enables improved predictions on a diverse test set of experimentally-characterized transition metal complexes by extrapolation from semi-local DFT to hybrid DFT. The ANN also outperforms other machine learning models (*i.e.*, support vector regression and kernel ridge regression), demonstrating particularly improved performance in transferability, as measured by prediction errors on the diverse test set. We establish the value of new uncertainty quantification tools to estimate ANN prediction uncertainty in computational chemistry, and we provide additional heuristics for identification of when a compound of interest is likely to be poorly predicted by the ANN. The ANNs developed in this work provide a strategy for screening transition metal complexes both with direct ANN prediction and with improved structure generation for validation with first principles simulation.

## Abstract 2

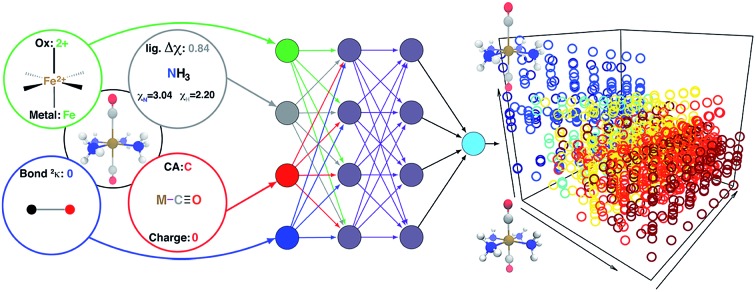
Our neural network predicts spin-state ordering of transition metal complexes to near-chemical accuracy with respect to DFT reference.

## 1. Introduction

High-throughput computational screening has become a leading component of the workflow for identifying new molecules,^1^,^2^ catalysts,^3^ and materials.^4^ First-principles simulation remains critical to many screening and discovery studies, but relatively high computational cost of direct simulation limits exploration of chemical space to a small fraction of feasible compounds.^5^,^6^ In order to accelerate discovery, lower levels of theory, including machine-learning models, have emerged as alternate approaches for efficient evaluation of new candidate materials.^7^ Artificial neural networks (ANNs) have recently found wide application in the computational chemistry community.^8^–^10^ Machine learning approaches were initially appreciated for their flexibility to fit potential energy surfaces and thus force field models.^10^–^17^ Broader applications have recently been explored, including in exchange–correlation functional development,^8^,^18^ general solutions to the Schrödinger equation,^19^ orbital free density functional theory,^20^,^21^ many body expansions,^22^ acceleration of dynamics,^23^–^25^ band-gap prediction,^26^,^27^ and molecular^1^,^2^ or heterogeneous catalyst^28^ and materials^29^–^32^ discovery, to name a few.

Essential challenges for ANNs to replace direct calculation by first-principles methods include the appropriate determination of broadly applicable descriptors that enable the use of the ANN flexibly beyond molecules in the training set, *e.g.* for larger molecules or for those with diverse chemistry. Indeed, the most successful applications of ANNs at this time beyond proof-of-concept demonstrations have been in the development of force fields for well-defined compositions, *e.g.* of water.^33^,^34^ Within organic chemistry, structural descriptors such as a Coulomb matrix^35^ or local descriptions of the chemical environment and bonding^36^,^37^ have been useful to enable predictions of energetics as long as a relatively narrow range of compositions is considered (*e.g.*, C, H, N, O compounds). These observations are consistent with previous successes in cheminformatics for evaluating molecular similarity,^38^ force field development,^39^ quantitative structure–activity relationships,^40^ and group additivity^41^ theories. For transition metal complexes, few force fields have been established that can capture a full range of inorganic chemical bonding,^42^ and the spin-state- and coordination-environment-dependence of bonding^43^ suggests that more careful development of descriptors is required to broadly predict properties of open-shell transition metal complexes. Similarly, descriptors that worked well for organic molecules have been demonstrated to not be suitable in inorganic crystalline materials.^44^ It is well-known^45^–^47^ that there is a strong relationship between sensitivity of electronic properties (*e.g.*, spin-state splitting) and the direct ligand–atom and ligand field strength^48^,^49^ in transition-metal complexes. Since ligands with the same direct metal-bonding atom can have substantially different ligand-field strengths (*e.g.*, C for both weaker field CH_3_CN *versus* strong-field CO), whereas distant substitutions (*e.g.*, tetraphenylporphyrin *vs.* base porphine) will have a limited effect, a transition-metal complex descriptor set that carefully balances metal-proximal and metal-distant descriptors is needed.

Within transition metal chemistry and correlated, inorganic materials, a second concern arises for the development of ANN predictions of first-principles properties. Although efficient correlated wavefunction theory methods (*e.g.*, MP2) may be straightforwardly applied to small organic molecules, such methods are not appropriate for transition metal complexes where best practices remain an open question.^50^ Although promising avenues for ANNs include the mapping of lower-level theory results, *e.g.* from semi-empirical theory,^51^ to a higher-level one, as has been demonstrated on atomization energies^52^ and more recently reaction barriers,^53^ suitable levels of theory for extrapolation are less clear in transition metal chemistry.

Additionally, uncertainty remains about the amount of exact (Hartree–Fock, HF) exchange to include in study of transition metal complexes, with recommendations ranging from no exchange, despite disproportionate delocalization errors in approximate DFT on transition metal complexes,^48^,^54^,^55^ to alternately low^56^–^58^ or high^59^ amounts of exact exchange in a system-dependent manner. Indeed, there has been much interest recently in quantifying uncertainty with respect to functional choice in energetic predictions,^60^–^62^ including through evaluation of sensitivity of predictions with respect to inclusion of exact exchange.^45^,^59^ Spin-state splitting is particularly sensitive to exchange fraction,^45^–^47^ making it a representative quantity for which it is useful to obtain both a direct value and its sensitivity to varying the exchange fraction. Thus, a machine-learning model that predicts spin-state ordering across exchange values will be useful for translating literature predictions or providing sensitivity measures on computed data.

Overall, a demonstration of ANNs in inorganic chemistry, *e.g.* for efficient discovery of new spin-crossover complexes,^63^,^64^ for dye-sensitizers in solar cells,^65^ or for identification of reactivity of open-shell catalysts^66^*via* rapid evaluation of spin-state ordering should satisfy two criteria: (i) contain flexible descriptors that balance metal-proximal and metal-distant features and (ii) be able to predict spin-state ordering across exchange–correlation mixing. In this work, we make progress toward both of these aims, harnessing cheminformatics-inspired transition metal-complex structure generation tools^67^ and established structure–functional sensitivity relationships in transition metal complexes^45^,^59^ to train ANNs for transition metal complex property prediction.

The outline of the rest of this work is as follows. In Section 2 (Methods), we review the computational details of data set generation, we discuss our variable selection procedure, and we review details of the artificial neural network trained. In Section 3, we provide the Results and discussion on the trained neural networks for spin-state ordering, spin-state exchange sensitivity, and bond-length prediction on both training-set-representative complexes and diverse experimental complexes. Finally, in Section 4, we provide our Conclusions.

## 2. Methods

### 2.1 Test set construction and simulation details

#### Data set construction

Our training set consists of octahedral complexes of first-row transition metals in common oxidation states: Cr^2+/3+^, Mn^2+/3+^, Fe^2+/3+^, Co^2+/3+^, and Ni^2+^. High-spin (H) and low-spin (L) multiplicities were selected for each metal from the ground, high-spin state of the isolated atom and the higher-energy, lowest-spin state within 5 eV that had a consistent d-orbital occupation for both states, as obtained from the National Institute of Standards and Technology atomic spectra database.^68^ The selected H–L states were: triplet-singlet for Ni^2+^, quartet-doublet for Co^2+^ and Cr^3+^, quintet-singlet for Fe^2+^ and Co^3+^, quintet-triplet for Cr^2+^ and Mn^3+^ (due to the fact that there is no data available for Mn^3+^ singlets^68^), and sextet-doublet for Mn^2+^ and Fe^3+^.

**Fig. 1:**
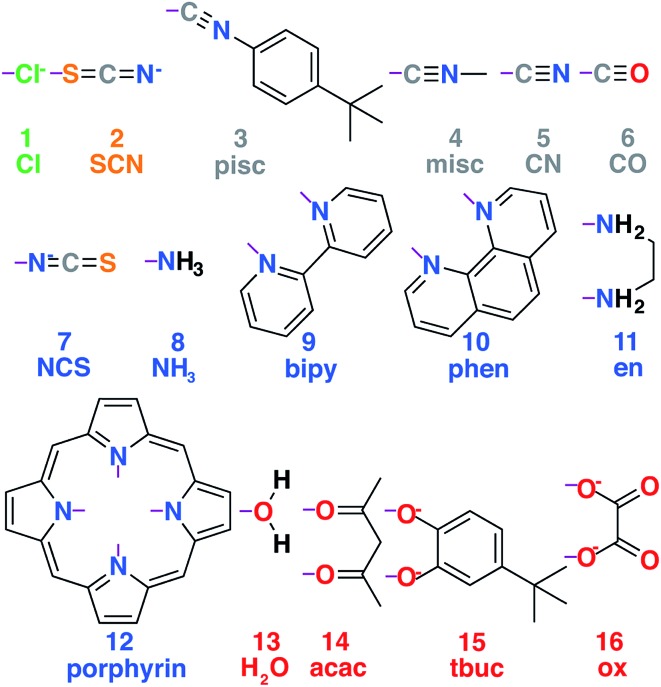
Set of ligands used to generate the transition metal complex data set. Ligands are numbered **1–16** and colored according to the atom type that coordinates with the metal, with chlorine in green, carbon in gray, sulfur in orange, nitrogen in blue, and oxygen in red. Purple lines indicate the bonds formed to metal-coordinating atoms in the ligand complexes. Abbreviations for each ligand used in the text are also shown. Full chemical names are provided in ESI Table S3.†

**Fig. 2:**
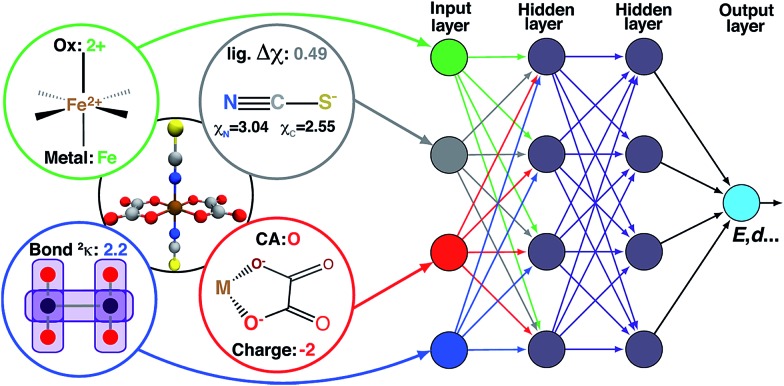
Schematic diagram of descriptors (left) as inputs to the ANN (right), along with hidden layers, and output (*e.g.*, spin-state splittings). The additive bias term in each node is omitted.

A set of common ligands in inorganic chemistry was chosen for variability in denticity, rigidity, and size (nine monodentate, six bidentate, and one tetradentate in Fig. 1 and ESI Table S1†). These ligands span the spectrochemical series from weak-field chloride (**1**, Cl^–^) to strong-field carbonyl (**6**, CO) along with representative intermediate-field ligands and connecting atoms, including S (**2**, SCN^–^), N (*e.g.*, **9**, NH_3_), and O (*e.g.*, **14**, acac). All possible homoleptic structures with all metals/oxidation states were generated from ten of these ligands (90 molecules) using the molSimplify toolkit^67^ (ESI Table S2†). Additional heteroleptic complexes (114 molecules) were generated using molSimplify with one mono- or bidentate axial ligand type (L_ax_) and an equatorial ligand type (L_eq_) of compatible denticity (ligands shown in Fig. 1, schematic shown in Fig. 2, geometries provided in the ESI†). We also selected 35 molecules from the Cambridge Structural Database^69^ (ESI Table S3†).

#### First-principles geometry optimizations

DFT gas-phase geometry optimizations were carried out using TeraChem.^70^,^71^ DFT calculations employ the B3LYP hybrid functional^72^–^74^ with 20% Hartree–Fock (HF) exchange (*a*_HF_ = 0.20) and a variant^45^ (*a*_HF_ = 0.00 to 0.30 in 0.05 increments) that holds the semi-local DFT portion of exchange in a constant ratio. We calculate and predict spin-state splitting sensitivities HF exchange, 
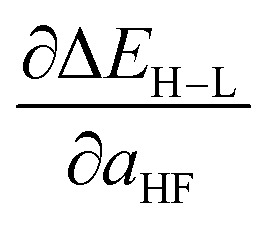
, as approximated from linear fits, in units of kcal per mol per HFX, where 1 HFX corresponds to varying from 0% to 100% HF exchange. B3LYP^72^–^74^ is chosen here due to its widespread use and our prior experience^45^ with tuning it to study HF exchange sensitivity, where we observed^45^ similar behavior with other GGA hybrids, *e.g.* PBE0, as long as the same HF exchange fraction was compared.

The composite basis set used consists of the LANL2DZ effective core potential^75^ for transition metals and the 6-31G* basis for the remaining atoms. All calculations are spin-unrestricted with virtual and open-shell orbitals level-shifted^76^ by 1.0 and 0.1 eV, respectively, to aid self-consistent field (SCF) convergence to an unrestricted solution.

For all training and test case geometry optimizations, default tolerances of 10^–6^ hartree for SCF energy changes between steps and a maximum gradient of 4.5 × 10^–4^ hartree per bohr were employed, as implemented in the DL-FIND interface^77^ with TeraChem (ESI Table S4†). Entropic and solvent effects that enable comparison to experimental spin-state splittings have been omitted, and we instead evaluate the DFT adiabatic electronic spin state splitting, as in previous work because our goal is to predict DFT properties and sensitivity to functional choice.^45^,^78^ In high-throughput screening efforts ongoing in our lab, entropic and solvent effects that influence catalytic and redox properties will be considered explicitly.

For each molecular structure (90 homoleptic, 114 heteroleptic) 14 geometry optimizations were carried out at 7 exchange fractions (from 0.00 to 0.30) and in high- or low-spin, for a theoretical maximum of 2856 geometry optimizations. In practice, 166 structures were excluded due to (i) large spin contamination, as defined by an expectation value of For each molecular structure (90 homoleptic, 114 heteroleptic) 14 geometry optimizations were carried out at 7 exchange fractions (from 0.00 to 0.30) and in high- or low-spin, for a theoretical maximum of 2856 geometry optimizations. In practice, 166 structures were excluded due to (i) large spin contamination, as defined by an expectation value of 〈*ŝ*^2^〉 that deviated more than 1 that deviated more than 1 *μ*_B_ from the exact value (<1%, 26 of 2856, see ESI Table S5†), (ii) dissociation in one or both spin states, especially of negatively charged ligands, leading to loss of octahedral coordination (4%, 126 of 2856, see ESI Table S6†), or (iii) challenges associated with obtaining a stable minimized geometry (<1%, 14 of 2856, see ESI Table S2†). Eliminating these cases produced a final data set of 2690 geometry optimizations (structures and energetics provided in ESI, as outlined in ESI Text S1†). Although these excluded cases are a fraction of our original data set, they highlight considerations for application of the ANN in high-throughput screening: highly negatively charged complexes should be avoided, and single point DFT calculations should be used to confirm that a high-fitness complex does not suffer from large ). Although these excluded cases are a fraction of our original data set, they highlight considerations for application of the ANN in high-throughput screening: highly negatively charged complexes should be avoided, and single point DFT calculations should be used to confirm that a high-fitness complex does not suffer from large 〈*ŝ*^2^〉 deviations. deviations.

### 2.2 Descriptor selection

High-throughput screening of transition-metal complex properties with direct prediction from an ANN requires mapping of an empirical feature space that represents the complex, *χ*, to quantum-mechanical predictions. This feature space should be balanced to avoid (i) too few descriptors with insufficient predictive capability or (ii) too many descriptors that lead to over-fitting of the ANN. Molecular descriptors^79^ that have been used for parameterizing chemical space include: atomic composition, electronegativity,^37^ formal charges, and representations of the geometric structure. This last class of descriptors may be divided into those that depend either on 3D structural information^13^,^20^,^80^–^82^ or on graph-theoretic connectivity maps^83^ (*e.g.*, the Randić,^84^ Wiener shape,^85^ or Kier^86^ indices). Graph-theoretic methods are preferable to 3D structural information to avoid sensitivity to translation/rotation or molecule size,^87^ though we note that subsystem descriptors^13^,^82^,^88^ and element-specific pairwise potentials^81^,^87^ have been employed successfully to overcome some challenges. A secondary reason to avoid use of 3D structural information is the implicit requirement of equilibrium geometries obtained from a geometry optimization, which are readily achieved with semi-empirical methods on small organic molecules^83^ but would be prohibitive and error-prone for transition metal complexes.

To evaluate candidate descriptor sets, we use L1-regularized, least absolute shrinkage and selection operator (LASSO) linear least-squares regression,^89^ as implemented in the glmnet^90^ package in R3.2.5.^91^ LASSO is used to reduce over-fitting, force the coefficients of the least-powerful indicators to zero, and avoid monotonic decrease of model error as feature space dimensionality increases. Given observed input–output pairs (**x**_*i*_, **y**_*i*_) for *i* = 1, …, *n* with **x** ∈ *χ* ⊂ ℝ_*i*_^*m*^ and *λ* ∈ ℝ, the output is modeled as:

1 **ỹ** = *β*^T^**X** + *β*_0_**1**

for *β*,*β*_0_ ∈ ℝ^*m*^ × ℝ, where:2
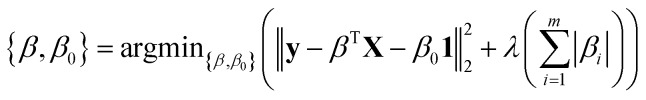



The parameter *λ* is selected by ten-fold cross-validation with values typically between 10^–1^ and 10^–6^. Our descriptors include both continuous variables that are normalized and discrete variables that are described by zero-one binary coding (ESI Table S7†). Metal identity is best described by a set of discrete variables: 4 binary variables are chosen to represent Cr, Mn, Fe, and Ni, and Co corresponds to the case where all 4 variables are zero. This leads to a higher number of overall variables than for continuous descriptors (see Table 1).

**Table 1:** Comparison of variable sets by root-mean-squared errors (RMSE) after regularization in Δ*E*_H–L_ and 
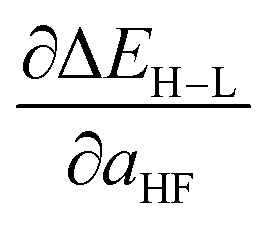
 prediction along with number of discrete variables (with all binary levels of the discrete variables counted in parentheses) and the number of continuous variables

Set	RMSE (Δ*E*_H–L_) (kcal mol^–1^)	RMSE 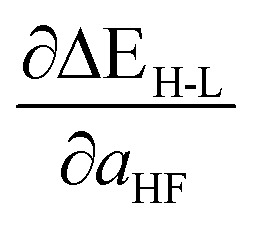 (kcal per mol per HFX)	Discrete variables	Continuous variables
a	14.6	20.6	3 (37)	6
b	15.1	21.7	3 (15)	8
c	15.2	21.2	3 (15)	11
d	15.1	21.3	3 (15)	10
e	14.9	21.1	3 (15)	12
f	15.1	23.5	3 (15)	10
g	14.9	21.3	3 (15)	12

Based on our previous studies of transition metal complexes,^45^,^49^ we expect that spin-state ordering is predominantly determined by the immediate chemical environment around the metal center, potentially enabling predictive descriptors that are widely transferable across a range of molecule sizes. We compare 7 descriptor sets on the data and select the subset of descriptors that give the best simultaneous predictive performance for spin-state splitting, Δ*E*_H–L_, and its sensitivity with respect to HF exchange variation, 
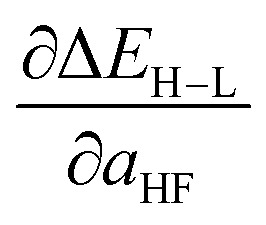
, as indicated by the prediction root mean squared error (RMSE):3
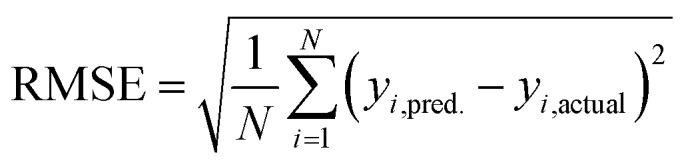



**Fig. 3:**
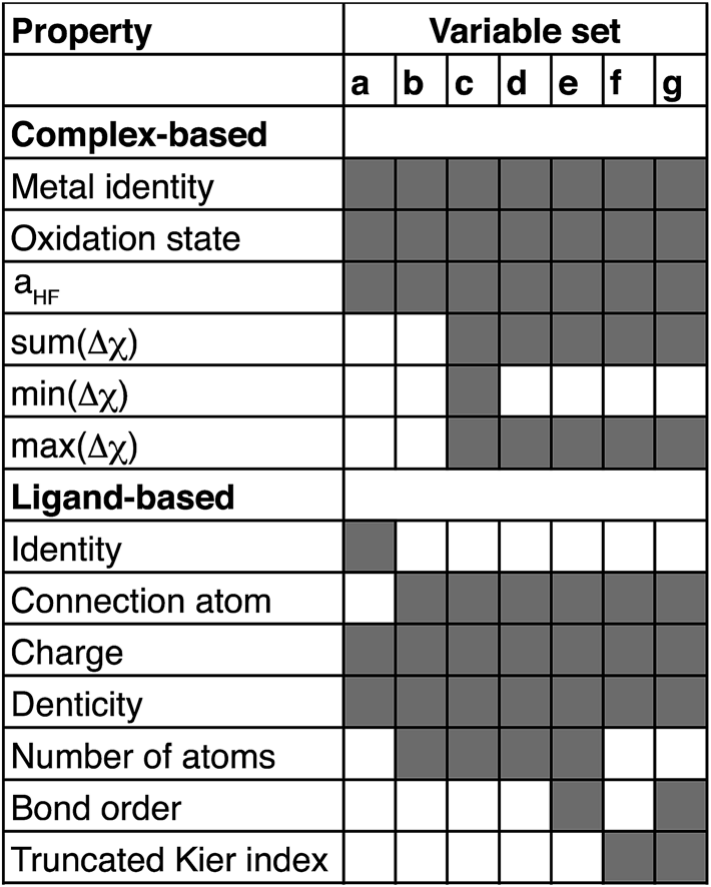
Summary of variables chosen for each set a through g. Employed variables are indicated in shaded gray and grouped by whether they are assessed on the whole complex (complex-based) or on each individual axial or equatorial ligand (ligand-based). Δ*χ* is the difference in Pauling electronegativity between the ligand connecting atom and all atoms bonded to it, and the sum, maximum or minimum values are obtained over all ligands.

When two variable sets perform comparably, we select the variable set that will enable broader application of the ANN. All sets include the metal identity as a discrete variable and metal oxidation state, ligand formal charge, and ligand denticity as continuous variables (Fig. 3, some descriptors shown in Fig. 2). Set a represents our most specific model, where we explicitly code the full axial or equatorial ligand identity as a discrete variable, limiting the application of the model but producing one of the lowest RMSEs for Δ*E*_H–L_ and 
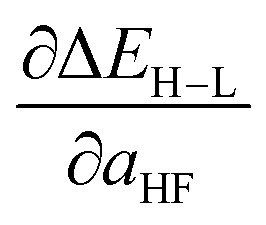
 (Table 1). Elimination of ligand identity in favor of ligand connecting atom elemental identity and total number of atoms in set b increases Δ*E*_H–L_ RMSE slightly and decreases 
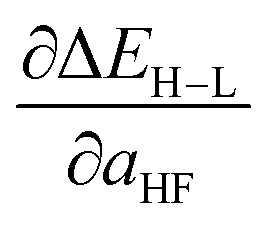
 RMSE (see Table 1).

The shift from set a to b increases the model applicability but at the cost of omitting subtler ligand effects. For instance, ethylenediamine (**11**, en) and phenanthroline (**10**, phen) have the same ligand charge/denticity and direct ligand atom (N), making them equivalent in set b except for the larger size of phen. System size alone is not expected to be a good predictor of field strength (*e.g.*, the small CO is one of the strongest field ligands). In set c, we introduce properties that depend on the empirical pairwise Pauling electronegativity difference (Δ*χ*) between the ligand connecting atom (LC) and any *i*th atom connected (CA) to it:

4 Δ*χ*_LC,*i*_ = *χ*_LC_ – *χ*_*i*_

These whole-complex differences include the maximum, max(Δ*χ*), and minimum, min(Δ*χ*), as well as sum:5
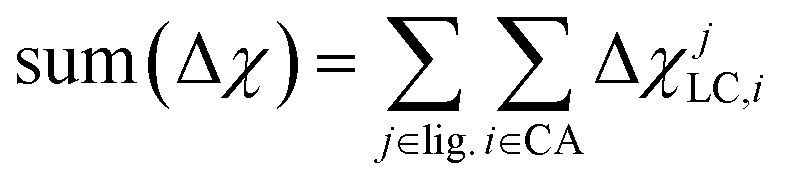
which is taken over the direct ligand atom and all atoms bonded to it for all ligands (lig.) in the complex. These additional set c descriptors reduce Δ*E*_H–L_ RMSE slightly and decrease the 
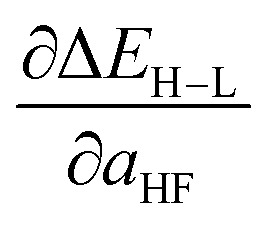
 RMSE to its lowest value (see Table 1). In set d, we eliminate min(Δ*χ*), expecting it to be redundant with the max and sum, at the cost of a small increase in both RMSEs.

Finally, in sets e–g, we replace ligand size (*i.e.*, number of atoms) with general descriptors to enable prediction on molecules larger than those in any training set. For example, tetraphenylporphyrin will have comparable electronic properties to unfunctionalized porphyrin (**12**), despite a substantial size increase. In set e, we introduce the maximum bond order of the ligand connecting atom to any of its nearest neighbors, a measure of the rigidity of the ligand environment, which is zero if the ligand is atomic (see ESI Table S1†). In set f, we eliminate the number of atoms and bond order metric, replacing them with a broader measure of the ligand geometry adjacent to the metal. After trial and error, we have selected the truncated Kier shape index,^86^^2^*κ*, which is defined by the inverse ratio of the square of number unique paths of length two (^2^*P*) in the molecular graph of heavy atoms to the theoretical maximum and minimum for a linear alkane with the same number of atoms:6
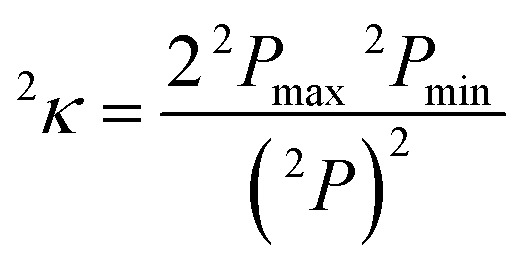
and set to zero for any molecules that do not have paths of length two. The truncation means that only the ligand atoms within three bonds of the connecting atom are included in the graph. The set f MSEs are comparable to or a slight increase from sets with molecule size, but they beneficially eliminate system size dependence. In set g, we reintroduce the bond order metric as well, providing the lowest MSEs except for set a or c, both of which are much less transferable than set g. Thus, the comparable performance of set g to a full ligand descriptor (set a) supports our hypothesis that a combination of metal-centric and ligand-centric in a heuristic descriptor set can be predictive and transferable. This final feature space is 15-dimensional with five per-complex descriptors and five per-ligand descriptors for each equatorial or axial ligand (see Table 2 for ranges of values and descriptions). A comparison of all errors and weights of variables across the seven data sets is provided in ESI Tables S7–S21 and Fig. S1.†

**Table 2:** Optimal (set g) input space descriptors and their range in the training set. Δ*χ* is the difference in Pauling electronegativity between the ligand connecting atom and all atoms bonded to it. When training the ANNs, a continuous descriptor corresponds to a single input node, whereas discrete descriptors correspond to one node per level

Symbol	Type	Descriptor	Values or range
**Whole-complex descriptors**			
M	Discrete	Metal identity	Cr, Mn, Fe, Co, Ni
O	Continuous	Oxidation state	2 to 3
me	Continuous	Max. Δ*χ* over all ligands	–0.89 to 1.20
se	Continuous	Sum of Δ*χ* over all ligands	–5.30 to 7.20
*a*_HF_	Continuous	HF exchange fraction	0.00 to 0.30

**Ligand-specific descriptors**			
L	Discrete	Ligand connection atom	Cl, S, C, N, or O
C	Continuous	Ligand charge	0 to –2
k	Continuous	Truncated Kier index	0.00 to 6.95
b	Continuous	Ligand bond order	0 to 3
D	Continuous	Ligand denticity	1 to 4

### 2.3 Training and uncertainty quantification of ML models

ANNs enable complex mapping of inputs to outputs^92^ beyond multiple linear regression and support the use of both discrete (*i.e.*, binary choices such as metal identity) and continuous (*e.g.*, the % of HF exchange) variables. Here, we apply an ANN with an input layer, two intermediate hidden layers, and an output layer (Fig. 2). The network topology was determined by trial and error, with additional hidden layers yielding no improved performance. All analysis is conducted in R3.2.5,^91^ using the h2o^93^ package with tanh non-linearity and linear output. Network weights and full training and test data are provided in the ESI.†

As with many ML models, ANNs are sensitive to over-fitting due to the number of weights to be trained.^94^ We address overfitting using dropout,^95^,^96^ wherein robustness of the fit is improved by zeroing out nodes in the network with an equal probability, *p*_drop_, at each stage of training (5% for spin-state splitting, 15% for HF exchange sensitivity, and 30% for bond lengths, selected by trial and error). Dropout has been shown to address overfitting when training feedforward ANNs on small datasets,^96^ with larger values of *p*_drop_ giving more aggressive regularization that worsens training errors but improves test errors. We use L2 weight regularization with a fixed penalty weight *λ*, as is applied in standard ridge regression, with an effective loss function for training:7
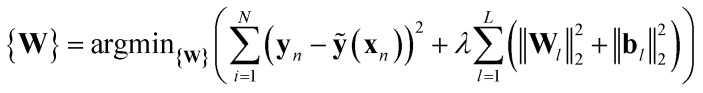
here, **W**_*l*_ refers to the weights from layer *l* to *l* + 1, **b**_*l*_ are biases at layer *l*, **ỹ**(**x**_*n*_) is the ANN prediction for the input–output pair (**x**_*n*_, **y**_*n*_), and the sums run over *N* training pairs and *L* layers.

During network training, we randomize the order of data points and partition the first 60% as training data and the last 40% for testing. Dropout networks, consisting of two hidden layers of 50 nodes each, are trained on the data set for varying values of *λ* ranging from 10^–1^ to 10^–6^ using 10-fold cross validation. For each λ, the training data is partitioned into ten groups, and a network is trained on each combination of nine of the groups and scored based on eqn (7) on the left-out group. The parameter with the lowest average prediction error is used to select the best regularization parameter: 5 × 10^–4^ for spin-state splitting, 10^–2^ for HF exchange sensitivity, and 10^–3^ for bond lengths. We varied and optimized^97^ the learning rate between 0.05 and 1.5, and optimal rates were selected as 1.0 (bond lengths) and 1.5 (spin-state splitting or HF exchange sensitivity). We use batch optimization for training (batch size = 20) for 2000 epochs. The training algorithm minimizes eqn (7) over the training data using stochastic gradient descent.^97^–^100^

It has not been possible to estimate ANN model uncertainty^95^,^101^ with the possible exception of bootstrapping^102^ by training the ANN on numerous subsamples of available training data. Model uncertainty will be due to either high-sensitivity to descriptor changes or test molecule distance in chemical space to training data (see also Section 3). Recent work^94^ showed that minimization of the loss function in eqn (7) is equivalent to approximate variational optimization of a Gaussian process (GP), making previously suggested ANN sampling for different dropout realizations^95^ a rigorously justified^94^ model uncertainty estimate.

We sample *J* distinct networks (in this work, *J* = 100) with different nodes dropped at the optimized weights and average over the predictions:8
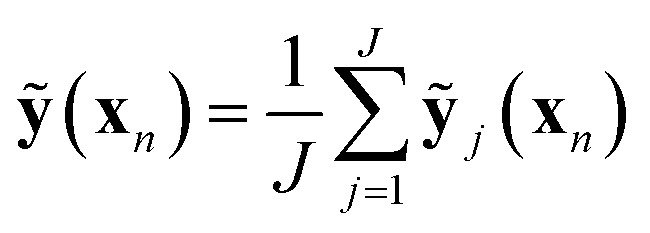
The ANN predictive variance is:^94^9
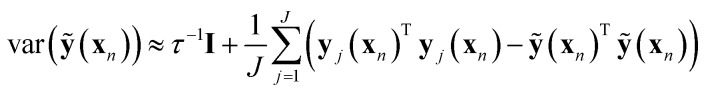
here, *τ* is10
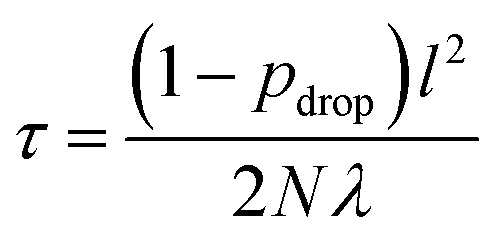
where *N* is the number of training data points, and *l* is a model hyperparameter for the GP that affects the estimation of predictive variance but does not enter into the ANN training. The contribution of *τ* in eqn (9) is a baseline variance inherent in the data, whereas the second term represents the variability of the GP itself. We obtain *τ* values of 0.6 for spin-state splitting, 0.07 for HF exchange sensitivity, and 10 000 for bond lengths (see Section 3). We choose *l* by maximizing the log predictive likelihood of the corresponding GP based on the training data (details are provided in the ESI Text S2†).

We selected an ANN based on the successful demonstrations^11^,^14^,^103^ of ANN-based models for predicting quantum chemical properties but also provide a comparison to two other common machine learning models:^89^ kernel ridge regression (KRR) and a support vector regression model (SVR), both using a square-exponential kernel. We used the R package kernlab^104^ and selected hyperparameters (the width of the kernel, and the magnitude of the regularization parameters which are given in the ESI Table S22†) using a grid search and ten-fold cross-validation using the R package CVST.^105^ We also compared training on our descriptor set to a KRR model with a kernel based on the L1 distance between sorted Coulomb matrix representations,^87^ as demonstrated previously.^52^,^103^

## 3. Results and discussion

### 3.1 Overview of data set spin-state energetics

**Fig. 4:**
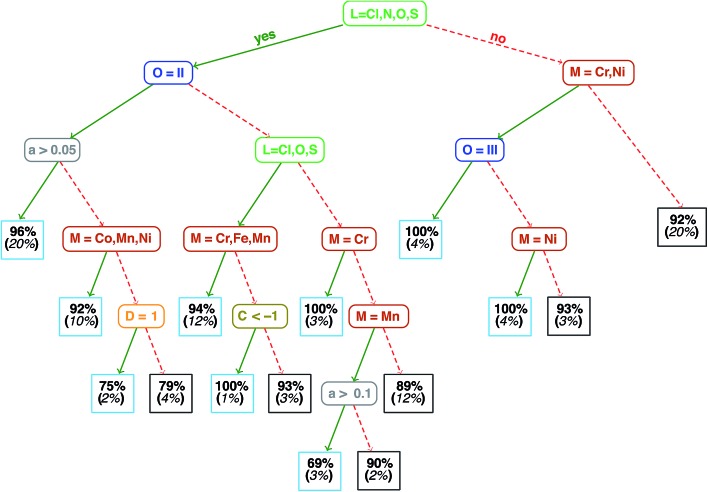
Binary ground state classification tree for homoleptic compounds. M indicates metal identity, L ligand connection atom, O oxidation state, a the fraction of HF exchange, C the charge, and D the ligand denticity. Each leaf node indicates the percent of elements in that leaf (light blue boxes for high-spin and dark gray boxes for low-spin) in bold font and percentage of total homoleptic population in the node (italic font, in parentheses).

Analysis of the qualitative and quantitative features of the spin-state splitting data set motivates the training of an ANN to move beyond ligand field arguments. We visualize qualitative ground states (*i.e.*, high-spin or low-spin) for the homoleptic subset of the data using a recursive binary tree (Fig. 4, descriptor definitions provided in Table 2), as previously outlined^106^ and implemented in the open source rpart package^107^ for R3.2.5.^91^ A recursive binary tree is a list of “branches” of the data ordered by statistical significance that gives the most homogeneous final “leaves” (here, with at least 10 data points) after a given number of permitted divisions (here, 6). Using descriptor set g, the data are partitioned into branches by testing which descriptors provide the “best” division to produce majority high- or low-spin states in leaves based on the concept of information impurity^107^ and pruning to remove statistically insignificant branches. The resulting electronic structure spectrochemical “tree” simultaneously addresses metal-specific strengths of ligands and exchange–correlation sensitivity. As expected, strong field direct carbon ligands (no Cl, N, O or S in Fig. 4) provide the root division of the tree, producing low-spin ground states for 92% of all Mn, Fe, and Co complexes (far right box on the third tier in Fig. 4). Next level divisions include the M(ii) oxidation state for *a*_HF_ > 0.05 that are predominantly (96%) high-spin. Spin-state ordering is well-known^45^,^59^ to be sensitive to HF exchange, and the tree reveals Mn^3+^ with nitrogen ligands to have the strongest *a*_HF_ dependence, since they are 69% high-spin for *a*_HF_ > 0.1 but 90% low-spin for *a*_HF_ ≤ 0.1. Extension of the recursive binary tree to heteroleptic compounds produces a second-level division based on sum(Δ*χ*), validating the relevance of the identified electronegativity descriptors for predicting heteroleptic spin-state ordering (ESI Fig. S2†).

**Fig. 5:**
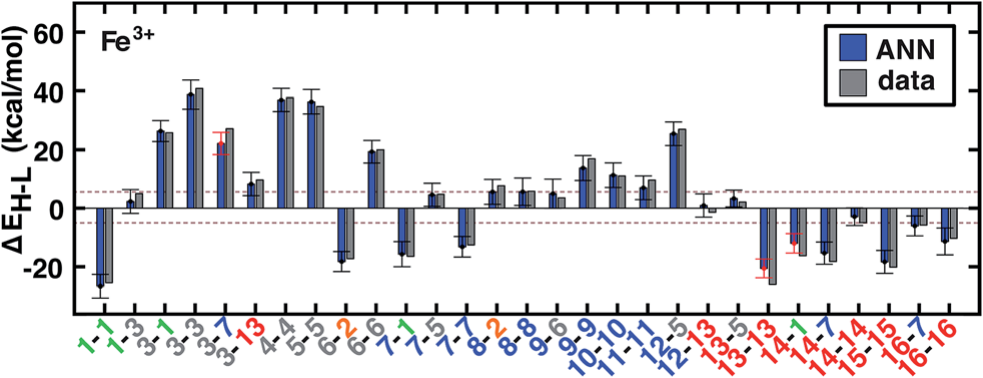
ANN model predicted (ANN, blue bars) and computed (data, gray bars) spin-state splittings, Δ*E*_H–L_, for the B3LYP functional (*a*_HF_ = 0.20) in kcal mol^–1^. Complexes are labeled by equatorial and then axial ligands according to the numbering indicated in Fig. 1 and color-coded by direct ligand atom (green for chlorine, gray for carbon, blue for nitrogen, red for oxygen, and orange for sulfur). The error bars represent an estimated ±1 standard deviation credible interval from the mean prediction, and error bars that do not encompass the computed value are highlighted in red. Brown dashed lines indicate a ±5 kcal mol^–1^ range around zero Δ*E*_H–L_, corresponding to near-degenerate spin states.

Quantitatively, the maximum Δ*E*_H–L_ in the data set is 90.7 kcal mol^–1^ for the strong-field Co(iii)(misc)_6_ complex at *a*_HF_ = 0.00, and the minimum value is –54.2 kcal mol^–1^ for the weak-field Mn(ii)(NCS^–^)_6_ at *a*_HF_ = 0.30. These extrema are consistent with (i) the ordering of metals in the spectrochemical series^43^ and (ii) the uniform effect of stabilizing high-spin states with increasing HF exchange. By comparing compound trends in the data set, we are able to identify whether additivity in ligand field effects, which has been leveraged previously in heuristic DFT correction models,^108^–^110^ is a universally good assumption. For the Fe(iii)(Cl^–^)_6–*n*_(pisc)_*n*_ complexes (denoted **1-1** through **3-3** in Fig. 5), increasing *n* from 0 to 2 through the addition of two axial pisc ligands increases the spin-state splitting by 15.1 kcal mol^–1^ per replaced chloride. Transitioning to a complex with all equatorial pisc ligands (*n* = 4) increases the spin-state splitting by only 10.4 kcal mol^–1^ per additional ligand, and the homoleptic structure pisc (*n* = 6) only adds 7.5 kcal mol^–1^ per additional ligand beyond the *n* = 4 case. An additive model cannot precisely reproduce diminishing ligand effects. As a stronger example for the need for nonlinear models such as an ANN, replacing two axial ligands from the strong-field Mn(ii)(CO)_6_ complex with the weaker-field NCS^–^ (**6-6** and **6-7** in ESI Fig. S3†) alters Δ*E*_H–L_ by <1 kcal mol^–1^, as strong-field ligands (*e.g.*, CO, CN^–^) have an overriding effect on spin-state splitting.

### 3.2 Spin-state splittings from an ANN

Motivated by non-linear effects in ligand additivity, we trained an ANN using a heuristic descriptor set (see Section 2.2) to predict qualitative spin-state ordering and quantitative spin-state splitting. The ANN predicts the correct ground state in 98% of the test cases (528 of 538) and 96% of training cases (777 of 807). All of the misclassifications are for cases in which DFT Δ*E*_H–L_ is <±5 kcal mol^–1^ (ESI Table S23†). The ANN spin-state prediction errors are not sensitive to HF exchange mixing, and thus our trained ANN is able to predict ground states of transition metal complexes from the pure GGA limit to hybrids with moderate exchange.

**Fig. 6:**
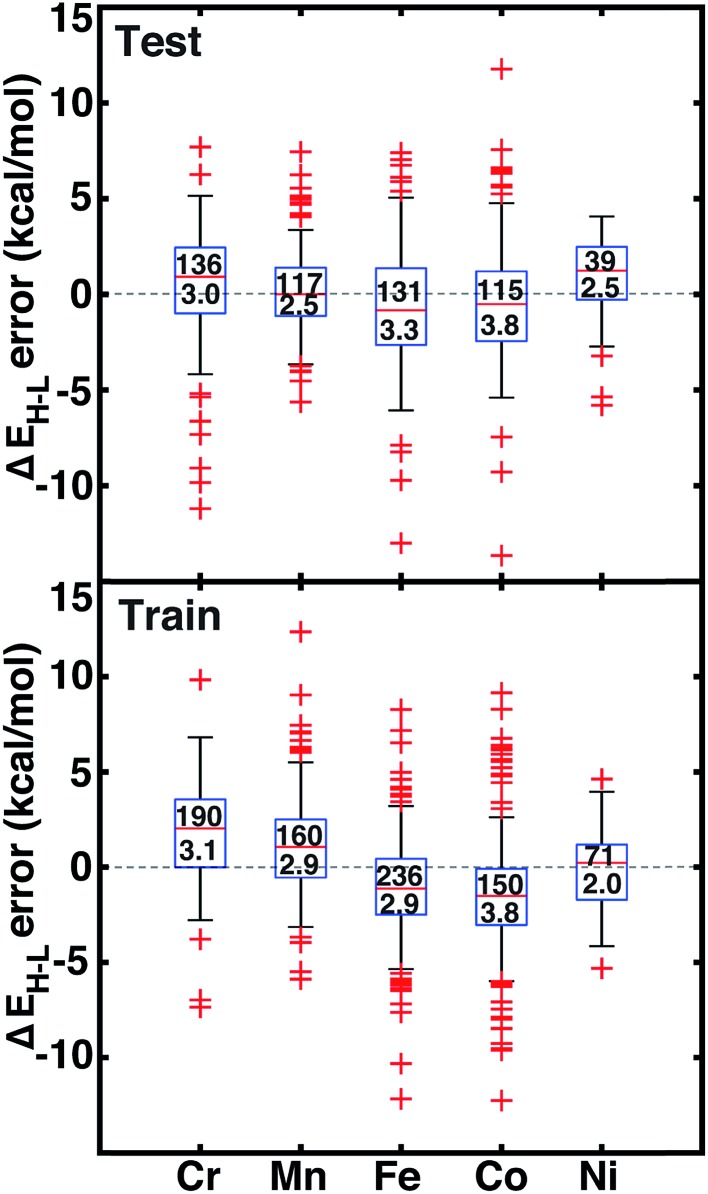
Error boxplots for Δ*E*_H–L_ in kcal mol^–1^ using the ANN for test (top) and training (bottom) data partitioned by metal identity. The top number inside the box indicates the number of cases in each set, and the bottom number indicates the RMSE in kcal mol^–1^. The range for both graphs is from 15 kcal mol^–1^ to –15 kcal mol^–1^.

We assess quantitative performance with root mean squared errors (RMSE) of the ANN (eqn (3)), overall and by metal (Fig. 6, ESI Table S24 and Fig. S3–S6†). The comparable RMSE of 3.0 and 3.1 kcal mol^–1^ for the training and test data, respectively, indicate an appropriate degree of regularization. The ANN predicts DFT spin-state splittings within 1 kcal mol^–1^ (*i.e.*, “chemical accuracy”) for 31% (168 of 538) of the test data and within 3 kcal mol^–1^ (*i.e.*, “transition metal chemical accuracy^111^”) for 72% (389 of 538) of the test data. Only a small subset of 49 (4) test molecules have errors above 5 (10) kcal mol^–1^, and correspond to strong-field Co and Cr complexes, *e.g.*, Cr(ii)(NCS^–^)_2_(pisc)_4_ (ESI Fig. S5†). The model is equivalently predictive for homoleptic and heteroleptic compounds at 2.2 and 2.3 kcal mol^–1^ average unsigned error respectively.

The training and test RMSEs broken down by metal reveal comparable performance across the periodic table (Fig. 6). Slightly higher test RMSEs (maximum unsigned errors) for Co and Fe complexes at 3.8 (15.7) and 3.3 (13.0) kcal mol^–1^, respectively, are due to the train/test partition and more variable ligand dependence of spin-state ordering in these complexes (Fig. 6 and ESI Table S24†). When the ANN performs poorly, the errors are due to both under- and over-estimation of Δ*E*_H–L_ for both strong- and weak-field ligands, regardless of HF exchange fraction: *e.g.*, Δ*E*_H–L_ for Co(iii)(CN^–^)_6_ at *a*_HF_ = 0.00 and Co(iii)(en)_3_ at *a*_HF_ = 0.20 are overestimated by 14 and 9 kcal mol^–1^, respectively, but Δ*E*_H–L_ for Fe(iii)(Cl^–^)_6_ at *a*_HF_ = 0.10 and Co(ii)(H_2_O)_2_(CN^–^)_4_ at *a*_HF_ = 0.30 and are underestimated by 9 and 7 kcal mol^–1^, respectively.

Quantified uncertainty estimates correspond to a baseline standard deviation in the model of approximately 1.5 kcal mol^–1^
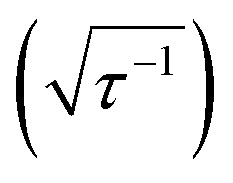
 and a mean total estimated standard deviation across the training and test cases of 3.8 and 3.9 kcal mol^–1^, respectively (see Section 2.3 and error bars on Fig. 5). These credible intervals are not rigorously confidence intervals but can highlight when prediction uncertainty is high: a ±1 (±2) standard deviation (std. dev.) interval on ANN predictions captures 83% (98%) of computed values for test set (see ESI Fig. S7†). Highest std. dev. values of around 5 kcal mol^–1^ are observed for Fe(ii) and Mn(ii) complexes and the lowest are around 3 for Cr and Co complexes (see ESI†). A single std. dev. around the ANN prediction contains the calculated Δ*E*_H–L_ for 26 of 29 Fe(iii) complexes at *a*_HF_ = 0.20 but misses heteroleptic oxygen coordinating complexes, **13-13** and **14-1**, and underestimates the effect of C/N ligands in **3-7** (Fig. 5). The model performs consistently across different ligand sizes, from porphyrin Fe(iii) complexes (**12-13**, **12-5**) to Fe(ii)(NH_3_)_6_ and Fe(ii)(CO)_6_ (**6-6** and **8-8**). For ligand-specific effects, the ANN performs well, reversing splitting magnitude as equatorial and axial ligands are swapped (*e.g.*, **1-3***versus***3-1**).

**Fig. 7:**
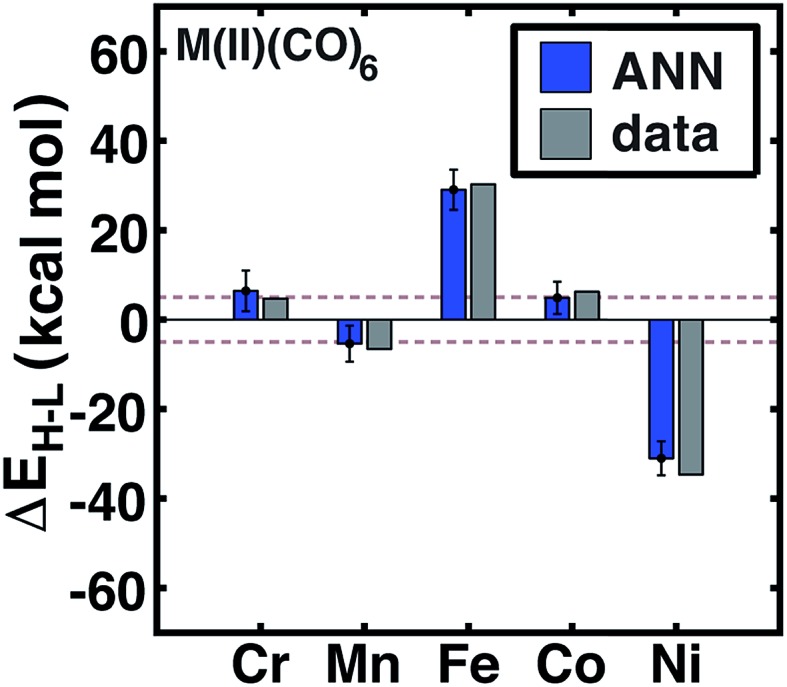
ANN model predicted (ANN, blue bars) and computed (data, gray bars) spin-state splittings, Δ*E*_H–L_, with the B3LYP functional (*a*_HF_ = 0.20) in kcal mol^–1^ on M(ii)(CO)_6_ complexes, where M = Cr, Mn, Fe, Co, or Ni. The error bars represent an estimated ±1 standard deviation credible interval from the mean prediction, and brown dashed lines indicate a ±5 kcal mol^–1^ range around zero Δ*E*_H–L_, corresponding to near-degenerate spin states.

Review of other metals/oxidation states reveals comparable performance for cases where the high-spin state is always favored (*e.g.*, Mn(ii), Cr(iii), or Ni(ii)), low-spin state is always favored (*e.g.*, Cr(iii)), and those where ligands have strong influence over the favored spin state (*e.g.*, Fe(ii) and Cr(ii)) (see ESI Fig. S3–S6†). For instance, metal-specific effects examined through comparison of M(ii)(CO)_6_ complexes (Fig. 7) reveal good ANN performance both for where the strong-field ligand strongly favors the low-spin state (*i.e.*, Fe and Ni) and where the spin-states are nearly degenerate (*i.e.*, Cr, Mn, Co). The trends outlined here for 20% HF exchange hold at other exchange mixing values (ESI Table S23†). Thus, our ANN trained on a modest data set with heuristic descriptors predicts spin-state splitting within a few kcal mol^–1^ of the DFT result.

Comparing our results to KRR, SVR, and LASSO regression reinforces the choice of an ANN (Table 3 and ESI Fig. S8†). The ANN outperforms KRR with either our descriptor set or the sorted Coulomb matrix descriptor both on the full data set or at fixed HF exchange (ESI Text S3†). The ANN also performs slightly better than SVR on test data with our descriptors. Linear LASSO regression was employed for feature selection (Section 2.2) but is outperformed by all other methods (Table 3). We will revisit the performance of these models on a more diverse molecule test set in Section 3.5 to assess the question of transferability.

**Table 3:** Train/test data and CSD test set RMSEs and max UEs for Δ*E*_H–L_ in kcal mol^–1^ for different machine learning methods and descriptor sets compared: KRR, kernel ridge regression, using square-exponential kernel for descriptor set g and the L1 matrix distance^52^ for the sorted Coulomb matrix descriptor; SVR, support vector regression using square-exponential kernel; ANN, artificial neural network. Results are also given for the KRR/Coulomb case, restricted to B3LYP only since the Coulomb matrix does not naturally account for varying HF exchange

Model	Descriptor	Training		Test		CSD	
		RMSE	Max UE	RMSE	Max UE	RMSE	Max UE
LASSO	Set g	16.1	89.7	15.7	93.5	19.2	72.5
KRR	Set g	1.6	8.5	3.9	17.0	38.3	88.4
SVR	Set g	2.1	20.9	3.6	20.4	20.3	64.8
ANN	Set g	3.0	12.3	3.1	15.6	13.1	30.4
KRR	Sorted Coulomb	4.3	41.5	30.8	103.7	54.5	123.9
KRR, B3LYP only	Sorted Coulomb	17.2	58.0	28.1	69.5	46.7	118.7

### 3.3 Predicting exchange sensitivity with an ANN

Spin-state splittings exhibit high sensitivity to exchange^45^,^59^ with linear behavior that we previously identified^45^ to be strongly dependent on direct ligand identity and field strength when we compared a set of Fe complexes. Over the current data set, computed exchange sensitivities are indeed linear, ranging from –174 kcal per mol per HFX for strong-field Fe(ii)(CO)_6_ to –13 kcal per mol per HFX for weak-field Cr(iii)(en)_2_(NH_3_)_2_. Cr(iii) is the least exchange-sensitive metal in our test set, whereas Fe(ii) and Mn(ii) are the most sensitive (ESI Table S25 and Fig. S9†).

**Fig. 8:**
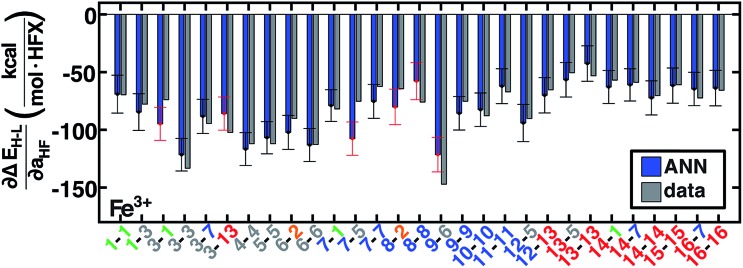
ANN model predicted (ANN, blue bars) and computed (data, gray bars) spin-state splitting sensitivities to HF exchange, 
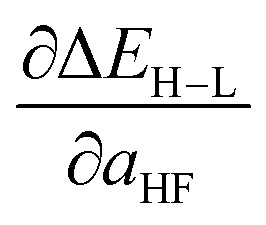
, in kcal per mol per HFX, for Fe^3+^ complexes. Complexes are labeled as equatorial and then axial ligands according to the numbering indicated in Fig. 1 and color-coded by direct ligand atom (green for chlorine, gray for carbon, blue for nitrogen, red for oxygen, and orange for sulfur). The error bars represent an estimated ±1 standard deviation credible interval from the mean prediction, and error bars that do not encompass the computed value are highlighted in red.

We therefore generalize previous observations^45^ in an ANN that predicts HF exchange sensitivity of spin-state ordering, 
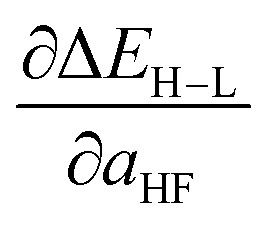
, using the same descriptors as for direct spin-state splitting, excluding only *a*_HF_. The smaller size of this data set (1/7 the size of the Δ*E*_H–L_ data set) leads to overfitting, with lower RMSE values of 13 kcal per mol per HFX for the training data *versus* 22 kcal per mol per HFX for the test set (Table 4, ESI Fig. S10 and Table S26†). Although results are reported in units of HFX (from 0 to 100% exchange), for typical 20% variation in exchange, a 20 kcal per mol per HFX sensitivity error only corresponds to a 4 kcal mol^–1^ energy difference. Both maximum unsigned errors (UE) and RMSEs are largest for Mn(ii/iii) and Cr(ii) complexes, with the largest case producing an 92 kcal per mol per HFX underprediction for Mn(iii)(H_2_O)_4_(pisc)_2_. Overall, the ANN prediction errors are less than less than 20 (40) kcal per mol per HFX for 65% (95%) of the test data. The ANN provides a valuable strategy for predicting exchange sensitivity, reproducing nonmonotonic and nonconvex ligand sensitivity in heteroleptic compounds: a Fe(iii) complex with ox, **16**, and NCS^–^, **7**, ligands is more sensitive to HFX than the respective homoleptic complexes (Fig. 8, other metals in ESI Fig. S11–S14†).

**Table 4:** Test set RMSEs for 
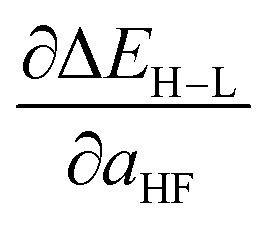
 in kcal per mol per HFX separated by metal and oxidation state along with minimum and maximum unsigned test errors (UE). The number of test cases is indicated in parentheses

Species	RMSE	Min. UE	Max. UE
Cr(ii)	21 (14)	4	45
Cr(iii)	17 (8)	2	37
Mn(ii)	24 (6)	3	40
Mn(iii)	38 (8)	4	92
Fe(ii)	18 (9)	2	41
Fe(iii)	15 (12)	<1	32
Co(ii)	17 (8)	<1	26
Co(iii)	20 (8)	<1	46
Ni(ii)	9 (4)	1	15

Uncertainty intervals of ANN predictions for HFX sensitivity yield a narrow range from 14 kcal per mol per HFX to 17 kcal per mol per HFX. For the 29 Fe(iii) complexes studied, 23 (80%) of the ANN credible intervals span the computed exchange sensitivity (Fig. 8). Across the full metal and oxidation state data set, 70% (83%) of the computed data is contained by ±1 (±2) std. dev. intervals (Fig. 8 and ESI S15†). This performance can be further improved by extending the training data. Exchange-sensitivity provides value both for extrapolation of computed (see Section 3.6) or literature values obtained at an arbitrary exchange mixing and in identification of cases of high-sensitivity to DFT functional choice.

### 3.4 Predicting equilibrium geometries with an ANN

**Fig. 9:**
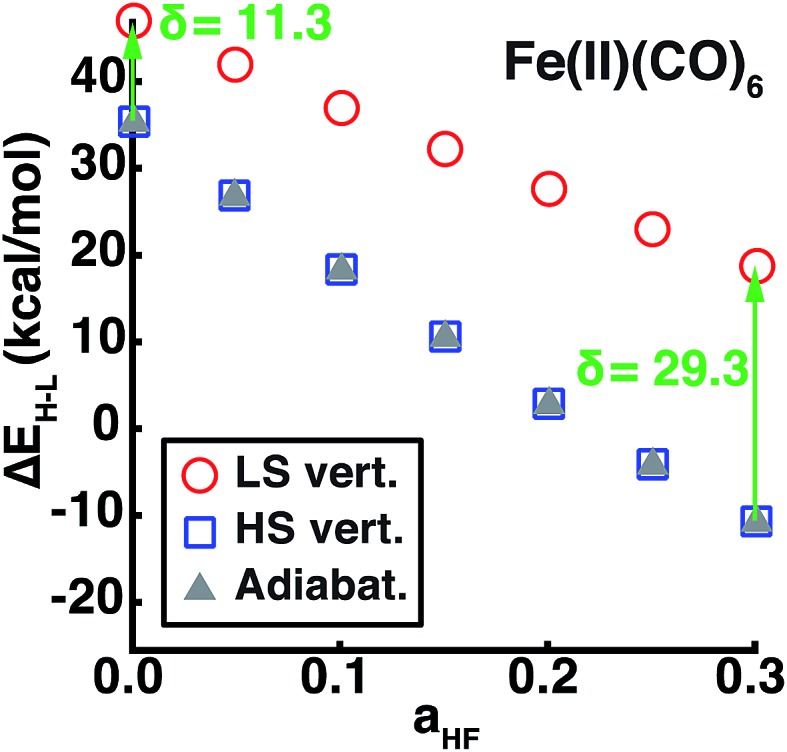
The vertical or adiabatic spin-state splittings, Δ*E*_H–L_, in kcal mol^–1^ as a function of HF exchange, *a*_HF_, for Fe(ii)(CO)_6_. Spin-state splittings evaluated at the HS or LS geometries are indicated by open blue squares and open red circles, respectively. The adiabatic spin-state splitting is shown as filled gray triangles. The HS vertical and adiabatic splittings overlap, whereas the LS vertical splitting overestimates Δ*E*_H–L_, as indicated by the green arrow and annotated *δ* in kcal mol^–1^ for *a*_HF_ = 0.00 and *a*_HF_ = 0.30.

Using our descriptor set, we trained an ANN on the minimum metal–ligand bond distances for both low-spin and high-spin geometries (min(*R*_LS/HS_)), which only differ from the exact metal–ligand bond length for distorted or heteroleptic compounds. This ANN for bond length prediction extends capabilities we have recently introduced for generating high-quality transition metal complex geometries^67^ in order to enable spin-state dependent predictions without requiring extended geometry-optimization. Furthermore, comparison of adiabatic and vertical spin-state splittings computed either at the low- or high-spin optimized geometries reveals that the vertical splitting at the HS geometry is indistinguishable from the adiabatic splitting, but the LS geometry vertical splitting favors the LS state by 10–30 kcal mol^–1^, increasing with *a*_HF_ (Fig. 9). Thus, if the ANN bond length predictions are accurate, adiabatic spin-state splittings can be obtained from DFT single points at ANN-predicted HS-only or both LS/HS geometries.

Metal–ligand bond distances in the *a*_HF_ = 0.20 data set vary from min(*R*_LS_) = 1.81 Å (in Fe(ii)(pisc)_2_(Cl^–^)_4_) to min(*R*_HS_) = 2.55 Å (in Fe(iii)(Cl^–^)_6_). The metal–ligand bond length ANN produces comparable RMSE across training (0.02 Å for LS and HS) and test (0.02 Å for LS and 0.03 Å for HS) data with comparable errors regardless of metal identity and oxidation- or spin-state (ESI Tables S27–S29 and Fig. S16–S27†). ANN bond length std. devs. range from 0.026 to 0.045 Å with a ∼0.01 Å baseline contribution. For low-spin (high-spin) complexes, 79% (81%) and 96% (96%) of the calculated values fall within one and two std. dev. of ANN-predicted bond lengths, respectively (ESI Fig. S17 and S23†).

**Fig. 10:**
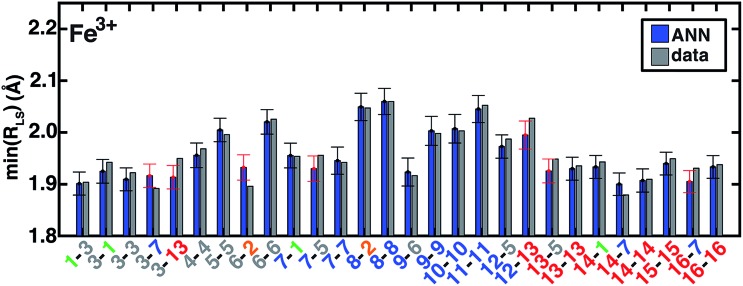
ANN model predictions (ANN, blue bars) and computed (data, gray bars) minimum LS Fe^3+^ bond lengths, min(*R*_LS_), in Å. Complexes are labeled as equatorial and then axial ligands according to the numbering indicated in Fig. 1 and color-coded by direct ligand atom (green for chlorine, gray for carbon, blue for nitrogen, and red for oxygen). The error bars represent an estimated ±1 standard deviation credible interval around the mean prediction, and error bars that do not encompass the computed value are highlighted in red. Fe(iii)(Cl)_6_ (**1-1**) is excluded due to being off scale: it has a predicted/calculated bond length of 2.44/2.45 Å, and an error standard deviation of ±0.02.

The ANN bond lengths fall outside of computed values for low-spin Fe(iii) complexes by more than a full standard deviation in seven cases, *e.g.*, underestimating Fe–C distances in CN (**7-5**, **13-5**) and pisc (**3-7**, **3-13**) complexes (Fig. 10). However, it also reproduces subtle trends, *e.g.* replacing axial ligands in homoleptic LS Fe(iii)(pisc)_6_ (**3-3** in Fig. 10, min(*R*_LS_) = 1.92 Å) with Cl^–^ increases the minimum bond distance to 1.94 Å (**3-1** in Fig. 10), but replacing equatorial pisc ligands instead with Cl^–^ (**1-3** in Fig. 10) decreases the minimum bond distance to 1.90 Å, a feature reproduced by the ANN. Non-additive bond length effects motivate the use of the ANN in initial geometry construction.^67^ Indeed, when we use ANN-predicted metal–ligand bond lengths in structure generation instead of our previous strategy based on a discrete database of DFT bond lengths,^67^ we reduce the metal–ligand component of the gradient by 54–90% (ESI Text S4, Fig. S28 and Table S30†). The ANN-predicted bond lengths and spin states are now available in molSimplify^67^ as an improved tool for structure generation.

### 3.5 Expanding the test set with experimental transition metal complexes

**Fig. 11:**
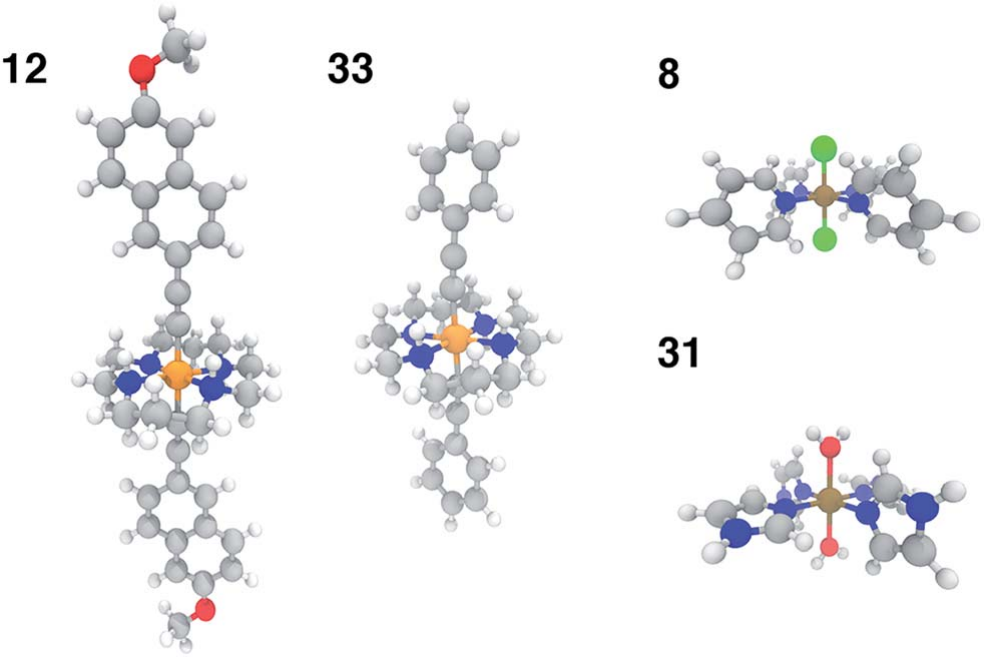
Representative CSD test set molecules shown in ball and stick representation with carbon atoms in gray, nitrogen atoms in blue, oxygen in red, hydrogen in white, chlorine in green, chromium in orange, and iron in brown. Test molecules 12 (CSD ID: SUMLET) and 33 (CSD ID: YUJCIQ) are Cr(iii) cyclams for which the ANN performs least well, and test molecules 8 (CSD ID: TPYFEC04) and 31 (CSD ID: BIPGEN) are cases for which the ANN predicts Δ*E*_H–L_ within 3 kcal mol^–1^.

In order to test the broad applicability of the trained ANNs, we selected 35 homoleptic and heteroleptic octahedral complexes from the Cambridge Structural Database^69^ (CSD) with a range of metals (Cr to Ni) and direct ligand atom types (N, C, O, S, Cl) (ESI Table S30†). The CSD test cases span a broader range of compounds than the training set, containing (i) larger macrocycles, *e.g.* substituted porphyrins (tests 9, 25), clathrochelates (test 16), phthalocyanines (tests 4, 7), and cyclams (tests 5, 12, 14, 17, 24, 29, and 33, 12 and 33 shown in Fig. 11) and (ii) coordination combinations or functional groups, *e.g.*, OCN in test 30, absent from the training set. Indeed, large CSD test molecule sizes, *e.g.* up to 103 atoms in a single equatorial ligand, further motivates our relatively size-independent descriptor set over forms that do not scale well with molecule size.

**Fig. 12:**
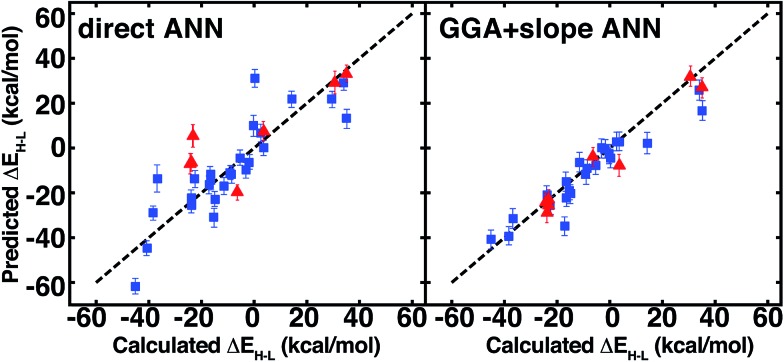
ANN spin-state splitting energy, Δ*E*_H–L_, predicted values on CSD test structures *vs.* DFT-calculated values, both at *a*_HF_ = 0.20 and in kcal mol^–1^. Direct prediction (left) is compared to GGA calculations and extrapolation using the predicted slope from the ANN (right). Error bars represent a credible interval of one standard deviation from the model uncertainty analysis (either in direct ANN at left or slope ANN at right), and a parity line (black, dashed) is indicated. Cyclams are indicated by red triangles, as described in main text, and the remaining test cases are indicated by blue squares.

The ANN predicts CSD test case spin-state splittings within 5 kcal mol^–1^ for 15 of the 35 complexes, an overall mean unsigned error of 10 kcal mol^–1^, and RMSE of 13 kcal mol^–1^ (see ESI Table S31†). The large RMSE is due in part to poor performance on early-transition-metal cyclams (red symbols in left panel of Fig. 12) for which the ANN overestimates spin-state splitting by at about 30 kcal mol^–1^ (Cr-cyclams, tests 12 and 33 in Fig. 11). The ANN predicts spin-state splittings within around 3 kcal mol^–1^ for several non-macrocyclic complexes that are better represented in the training data (*e.g.*, test cases 8 and 31 in Fig. 11). The correct ground state is assigned in 90% of CSD test cases (96% after excluding cyclams); the only incorrect, non-cyclam spin state assignment is a spin-crossover complex, test 25 (calculated Δ*E*_H–L_ = –0.2 kcal mol^–1^). Compared to other machine learning models (KRR and SVR), the ANN is more transferable to dissimilar CSD structures (Table 3), outperforming the next-best model, SVR, by 30%. The relative success of the ANN on the CSD data is partially attributable to the use of dropout regularization, which has been shown^96^ to improve robustness.

The observation of good performance with reasonable similarity between CSD structures and the training data but poor performance when the CSD structure is not well-represented motivates a quantitative estimate of test compound similarity to training data. We first computed overall molecular similarity metrics (*e.g.*, FP2 fingerprint *via* Tanimoto,^38^,^112^ as implemented in OpenBabel^113^,^114^) but found limited correlation (*R*^2^ = 0.1) to prediction error (see ESI Fig. S29 and Text S5†). Comparing the Euclidean and uncentered Pearson distances in descriptor space between the CSD test cases and the closest training data descriptors provides improved correlation to prediction error of *R*^2^ = 0.3 and *R*^2^ = 0.2, respectively (ESI Fig. S30†). Large errors (*i.e.*, >15 kcal mol^–1^) are only observed at a Euclidean norm difference exceeding 1.0 (half of the CSD data), providing an indication of lack of reliability in ANN prediction. This high distance to training data does not guarantee inaccurate prediction, *e.g.*, CSD test case 8, a Fe(ii) tetrapyridine complex, is predicted with fortuitously good ∼2 kcal mol^–1^ error but has a Euclidean norm difference >1.4. We have implemented the Euclidean norm metric alongside the ANN in our automated screening code^67^ to detect complexes that are poorly represented in training data and advise retraining or direct calculation.

ANN-predicted equilibrium metal–ligand bond lengths for both HS and LS CSD geometries produced RMSEs of 0.10 and 0.07 Å, respectively (ESI Tables S32 and S33†). Trends in bond length prediction error differ from those obtained for spin-state splitting. For instance, bond length errors are average in the cyclams even though spin-state splitting predictions were poor. The large Euclidean distance to training data heuristic (>1.0) is observed for five of the seven large (*i.e.*, >0.1 Å) HS bond distance errors (see ESI Texts S4 and S5, Fig. S31 and S32†). The highest HS prediction errors (>0.2 Å) occur for tests 8 and 35, underestimating the Fe–N bond length by 0.2 Å (2.1 Å ANN *vs.* 2.3 Å calculated) in the former case. Despite poor geometric predictions, the ANN predicts test 8 Δ*E*_H–L_ to within 3 kcal mol^–1^, and this differing performance is due to the fact that predictions of these two outputs are independent. Interligand effects that are ignored by our descriptor set can restrict bond length extension, *e.g.* in test 16, where an O–H···O^–^ interligand hydrogen-bond produces an unusually short 1.9 Å high-spin Fe–N bond distance (*vs.* ANN prediction of 2.1 Å). Future work will focus on incorporating extended metrics of rigidity to account for these effects.

**Fig. 13:**
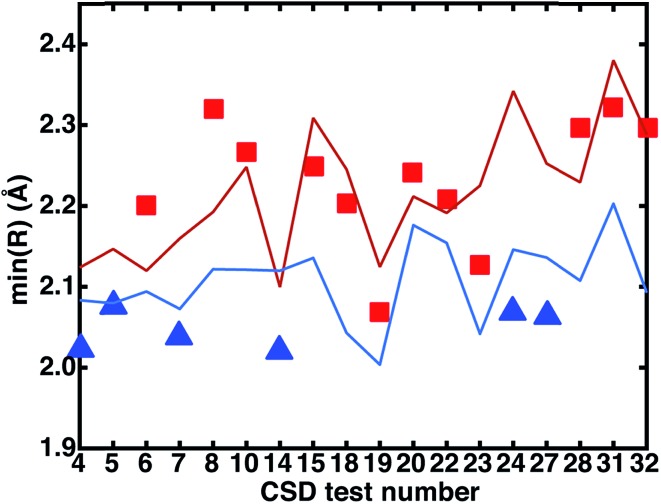
Comparison of measured CSD bond distances in the crystal phase, represented by symbols (red squares for high-spin or blue triangles for low-spin based on DFT assignment at *a*_HF_ = 0.20) with the ANN predicted HS (red line) and LS (blue line) bond distances. Only the CSD test cases where the difference between DFT LS and HS computed bond distances is ≥0.05 Å are shown for clarity. For all of these cases, the ANN correctly predicts the DFT spin state.

We investigated the relationship between the experimental CSD bond distances and the ANN-predicted bond distances. If the experimentally measured bond distance lies close to one spin state's predicted bond length, then the complex may be expected to be in that spin state, assuming (i) the ANN provides a good prediction of the spin-state specific bond lengths and (ii) that the gas-phase optimized DFT and CSD bond distances are comparable. The majority of experimental bond lengths are near the extrema of the ANN predictions (subset with a calculated DFT LS–HS bond distance of at least 0.05 Å shown in Fig. 13 to improve readability, full set in ESI Fig. S33†). Nine of the twelve (9 of 9 in Fig. 13) experimental bond lengths that are on or above the predicted HS bond distance boundary have an HS ground state, eleven of the fifteen (6 of 6 in Fig. 13) experimental bond lengths that are on or below the predicted LS bond distance have an LS ground state, and remaining structures (3 in Fig. 13) reside at intermediate distances. Some discrepancies are due to differences between the gas phase geometries and those in the crystal environment (*e.g.*, test 27 in Fig. 13 and see ESI Tables S31–S33†). This bond-length-based spin-assignment thus provides a strategy for corroboration of direct spin-state prediction.

### 3.6 Extrapolating pure exchange–correlation functionals to hybrids with an ANN

Linear spin-state HF exchange sensitivity may be exploited to predict properties at one *a*_HF_ value from computed properties obtained at another, *e.g.*, to translate literature values or to accelerate periodic, plane-wave calculations where incorporation of HF exchange increases computational cost. We carry out comparison of the utility of this Δ-ML-inspired^52^ strategy on the 35 CSD test set to identify if prediction errors are improved, especially for molecules poorly-represented in the training set.

On the CSD molecules, extrapolating *a*_HF_ = 0.00 spin-state ordering to *a*_HF_ = 0.20 with the exchange-sensitivity ANN reduces the maximum error to 23 kcal mol^–1^ and decreases the mean unsigned error and RMSE to 5 kcal mol^–1^ and 7 kcal mol^–1^ (the right pane of Fig. 11 and ESI Table S34†). For the GGA + slope ANN approach, excluding the nine cyclams does not change the RMSE/MUE values, confirming good ANN exchange-sensitivity prediction even when spin-state splitting prediction is poor.

These reduced average errors are quite close to the uncertainty introduced by the slope prediction performance at around 4 kcal mol^–1^ over a 20% exchange interval. Although this approach does eliminate the largest outliers and improve prediction across the CSD test set, it necessitates semi-local DFT geometry optimizations or a judicious bond length choice for vertically-approximated spin-state ordering. This approach also has limited benefit for cases well-represented in the training data set due to the sparser data set in the exchange sensitivity ANN. Indeed, over the original test set molecules, extrapolated ANN exchange sensitivities on top of calculated *a*_HF_ = 0.00 splittings produce an RMSE of around 4 kcal mol^–1^ comparable to or slightly worse than direct prediction (ESI Fig. S34†).

## 4. Conclusions

We have presented a series of ANN models trained using 2690 DFT geometry optimizations of octahedral transition metal complexes generated from a set of 16 candidate axial and equatorial ligands and transition metals (Cr–Ni) at varying fractions of HF exchange. From the unseen test cases of a 60–40% train-test partition, we demonstrated good accuracy on spin-state splitting predictions of around 3 kcal mol^–1^ and metal–ligand bond distances around 0.02–0.03 Å. Our simple descriptor set, including: (i) the ligand connection atom, (ii) electronegativity and bonding of the coordinating ligand atom environment, (iii) ligand formal charge, (iv) ligand denticity, and (v) metal identity and oxidation state ensures transferability of the ANN. Importantly, the employed connectivity models are not 3D-structure-based, instead relying on a truncated graph-theoretic representation of the ligand, making the approach suitable for screening large numbers of complexes without precise structural information. Although we have trained ANNs to predict bond lengths and spin-state splitting, the data set and descriptors could be used to predict other quantities such as ionization potential, redox potential, or molecular orbital energies. Such efforts are currently underway in our lab.

A test of our ANN on diverse molecules obtained from an experimental database indicated good performance, with MUEs of 5 kcal mol^–1^ for spin states for compounds within our proposed Euclidean distance reliability criteria and 10 kcal mol^–1^ for the full set. In both diverse and representative cases, the ANN outperforms other machine learning models. Our ANN predictions of HF exchange sensitivity provide a tool for interpolating between exchange–correlation functionals or extrapolating from semi-local GGAs to a hybrid result, which we demonstrated on CSD cases, improving MUE to 5 kcal mol^–1^ across the full 35 molecule set.

Natural extensions to this work include the development of the current ANN for extrapolation of GGA to hybrid functional properties in condensed matter systems and generalizing the coordination definition to enable prediction of properties of unsaturated metals in catalytic cycles. Overall, we have demonstrated a relatively sparse feature space to be capable of predicting electronic structure properties of transition metal complexes, and we anticipate that this strategy may be used for both high-throughput screening with knowledge of functional choice sensitivity and in guiding assessment of sources of errors in approximate DFT.

## Supplementary Material

Supplementary information

Supplementary information

Supplementary information

## References

[cit1] Gomez-Bombarelli R., Aguilera-Iparraguirre J., Hirzel T. D., Duvenaud D., Maclaurin D., Blood-Forsythe M. A., Chae H. S., Einzinger M., Ha D. G., Wu T., Markopoulos G., Jeon S., Kang H., Miyazaki H., Numata M., Kim S., Huang W., Hong S. I., Baldo M., Adams R. P., Aspuru-Guzik A. (2016). Nat. Mater..

[cit2] Pyzer-Knapp E. O., Li K., Aspuru-Guzik A. (2015). Adv. Funct. Mater..

[cit3] Norskov J. K., Bligaard T. (2013). Angew. Chem., Int. Ed. Engl..

[cit4] Jain A., Ong S. P., Hautier G., Chen W., Richards W. D., Dacek S., Cholia S., Gunter D., Skinner D., Ceder G. (2013). APL Mater..

[cit5] Virshup A. M., Contreras-García J., Wipf P., Yang W., Beratan D. N. (2013). J. Am. Chem. Soc..

[cit6] Kirkpatrick P., Ellis C. (2004). Nature.

[cit7] Meredig B., Agrawal A., Kirklin S., Saal J. E., Doak J. W., Thompson A., Zhang K., Choudhary A., Wolverton C. (2014). Phys. Rev. B: Condens. Matter Mater. Phys..

[cit8] Li L., Snyder J. C., Pelaschier I. M., Huang J., Niranjan U.-N., Duncan P., Rupp M., Müller K.-R., Burke K. (2016). Int. J. Quantum Chem..

[cit9] Rupp M. (2015). Int. J. Quantum Chem..

[cit10] Behler J. (2016). J. Chem. Phys..

[cit11] Behler J. (2014). J. Phys.: Condens. Matter.

[cit12] Lorenz S., Groß A., Scheffler M. (2004). Chem. Phys. Lett..

[cit13] Artrith N., Morawietz T., Behler J. (2011). Phys. Rev. B: Condens. Matter Mater. Phys..

[cit14] Behler J., Parrinello M. (2007). Phys. Rev. Lett..

[cit15] Prudente F. V., Neto J. J. S. (1998). Chem. Phys. Lett..

[cit16] Mones L., Bernstein N., Csanyi G. (2016). J. Chem. Theory Comput..

[cit17] Smith J. S., Isayev O., Roitberg A. E. (2017). Chem. Sci..

[cit18] Snyder J. C., Rupp M., Hansen K., Muller K.-R., Burke K. (2012). Phys. Rev. Lett..

[cit19] Mills K., Spanner M. and Tamblyn I., Deep Learning and the Schrödinger Equation, arXiv preprint arXiv:1702.01361, 2017.

[cit20] Yao K., Parkhill J. (2016). J. Chem. Theory Comput..

[cit21] Snyder J. C., Rupp M., Hansen K., Blooston L., Müller K.-R., Burke K. (2013). J. Chem. Phys..

[cit22] Yao K., Herr J. E., Parkhill J. (2017). J. Chem. Phys..

[cit23] Hase F., Valleau S., Pyzer-Knapp E., Aspuru-Guzik A. (2016). Chem. Sci..

[cit24] Li Z., Kermode J. R., De Vita A. (2015). Phys. Rev. Lett..

[cit25] Botu V., Ramprasad R. (2015). Int. J. Quantum Chem..

[cit26] Pilania G., Gubernatis J., Lookman T. (2017). Comput. Mater. Sci..

[cit27] Pilania G., Mannodi-Kanakkithodi A., Uberuaga B. P., Ramprasad R., Gubernatis J. E., Lookman T. (2016). Sci. Rep..

[cit28] Ma X., Li Z., Achenie L. E. K., Xin H. (2015). J. Phys. Chem. Lett..

[cit29] Mannodi-Kanakkithodi A., Pilania G., Huan T. D., Lookman T., Ramprasad R. (2016). Sci. Rep..

[cit30] Huan T. D., Mannodi-Kanakkithodi A., Ramprasad R. (2015). Phys. Rev. B: Condens. Matter Mater. Phys..

[cit31] Pilania G., Wang C., Jiang X., Rajasekaran S., Ramprasad R. (2013). Sci. Rep..

[cit32] Lee J., Seko A., Shitara K., Tanaka I. (2016). Phys. Rev. B.

[cit33] Morawietz T., Behler J. (2013). J. Phys. Chem. A.

[cit34] Morawietz T., Singraber A., Dellago C., Behler J. (2016). Proc. Natl. Acad. Sci. U. S. A..

[cit35] Rupp M., Tkatchenko A., Müller K.-R., von Lilienfeld O. A. (2012). Phys. Rev. Lett..

[cit36] Huang B., von Lilienfeld O. A. (2016). J. Chem. Phys..

[cit37] De S., Bartk A. P., Csányi G., Ceriotti M. (2016). Phys. Chem. Chem. Phys..

[cit38] Maggiora G., Vogt M., Stumpfe D., Bajorath J. (2013). J. Med. Chem..

[cit39] Wang J., Wolf R. M., Caldwell J. W., Kollman P. A., Case D. A. (2004). J. Comput. Chem..

[cit40] Kubinyi H. (1997). Drug Discovery Today.

[cit41] Benson S. W., Cruickshank F., Golden D., Haugen G. R., O'neal H., Rodgers A., Shaw R., Walsh R. (1969). Chem. Rev..

[cit42] Deeth R. J. (2001). Coord. Chem. Rev..

[cit43] Shriver D. F. and Atkins P. W., Inorganic Chemistry, W. H. Freeman and Co., 3rd edn, 1999.

[cit44] Schütt K. T., Glawe H., Brockherde F., Sanna A., Müller K. R., Gross E. K. U. (2014). Phys. Rev. B: Condens. Matter Mater. Phys..

[cit45] Ioannidis E. I., Kulik H. J. (2015). J. Chem. Phys..

[cit46] Ashley D. C., Jakubikova E. (2017). Coord. Chem. Rev..

[cit47] Bowman D. N., Jakubikova E. (2012). Inorg. Chem..

[cit48] Gani T. Z. H., Kulik H. J. (2016). J. Chem. Theory Comput..

[cit49] Ioannidis E. I., Kulik H. J. (2017). J. Phys. Chem. A.

[cit50] Huang W., Xing D.-H., Lu J.-B., Long B., Schwarz W. E., Li J. (2016). J. Chem. Theory Comput..

[cit51] Stewart J. J. P. (2013). J. Mol. Model..

[cit52] Ramakrishnan R., Dral P. O., Rupp M., von Lilienfeld O. A. (2015). J. Chem. Theory Comput..

[cit53] Shen L., Wu J., Yang W. (2016). J. Chem. Theory Comput..

[cit54] Kulik H. J. (2015). J. Chem. Phys..

[cit55] Cohen A. J., Mori-Sanchez P., Yang W. (2008). Science.

[cit56] Salomon O., Reiher M., Hess B. A. (2002). J. Chem. Phys..

[cit57] Reiher M. (2002). Inorg. Chem..

[cit58] Reiher M., Salomon O., Hess B. A. (2001). Theor. Chem. Acc..

[cit59] Droghetti A., Alfé D., Sanvito S. (2012). J. Chem. Phys..

[cit60] Sutton J. E., Guo W., Katsoulakis M. A., Vlachos D. G. (2016). Nat. Chem..

[cit61] Simm G. N., Reiher M. (2016). J. Chem. Theory Comput..

[cit62] Walker E., Ammal S. C., Terejanu G. A., Heyden A. (2016). J. Phys. Chem. C.

[cit63] Halcrow M. A. (2011). Chem. Soc. Rev..

[cit64] Létard J.-F., Guionneau P. and Goux-Capes L., Towards Spin Crossover Applications, in Spin Crossover in Transition Metal Compounds III, Springer, 2004, pp. 221–249.

[cit65] Bignozzi C. A., Argazzi R., Boaretto R., Busatto E., Carli S., Ronconi F., Caramori S. (2013). Coord. Chem. Rev..

[cit66] Harvey J. N., Poli R., Smith K. M. (2003). Coord. Chem. Rev..

[cit67] Ioannidis E. I., Gani T. Z. H., Kulik H. J. (2016). J. Comput. Chem..

[cit68] A. Kramida, Y. Ralchenko and J. Reader, NIST ASD Team NIST Atomic Spectra Database (Version 5.3), http://physics.nist.gov/asd, accessed March 14, 2017.

[cit69] Groom C. R., Bruno I. J., Lightfoot M. P., Ward S. C. (2016). Acta Crystallogr., Sect. B: Struct. Sci., Cryst. Eng. Mater..

[cit70] Ufimtsev I. S., Martinez T. J. (2009). J. Chem. Theory Comput..

[cit71] Petachem, http://www.petachem.com. accessed March 14, 2017.

[cit72] Stephens P. J., Devlin F. J., Chabalowski C. F., Frisch M. J. (1994). J. Phys. Chem..

[cit73] Becke A. D. (1993). J. Chem. Phys..

[cit74] Lee C., Yang W., Parr R. G. (1988). Phys. Rev. B: Condens. Matter Mater. Phys..

[cit75] Hay P. J., Wadt W. R. (1985). J. Chem. Phys..

[cit76] Saunders V. R., Hillier I. H. (1973). Int. J. Quantum Chem..

[cit77] Kästner J., Carr J. M., Keal T. W., Thiel W., Wander A., Sherwood P. (2009). J. Phys. Chem. A.

[cit78] Ganzenmuller G., Berkaine N., Fouqueau A., Casida M. E., Reiher M. (2005). J. Chem. Phys..

[cit79] Cereto-Massague A., Ojeda M. J., Valls C., Mulero M., Garcia-Vallve S., Pujadas G. (2015). Methods.

[cit80] Sheridan R. P., Miller M. D., Underwood D. J., Kearsley S. K. (1996). J. Chem. Inf. Model..

[cit81] Hansen K., Biegler F., Ramakrishnan R., Pronobis W. (2015). J. Phys. Chem. Lett..

[cit82] Gastegger M., Marquetand P. (2015). J. Chem. Theory Comput..

[cit83] Hageman J. A., Westerhuis J. A., Frhauf H. W., Rothenberg G. (2006). Adv. Synth. Catal..

[cit84] Randic M. (1975). J. Am. Chem. Soc..

[cit85] Wiener H. (1947). J. Am. Chem. Soc..

[cit86] Kier L. B. (1985). Quant. Struct.-Act. Relat..

[cit87] Montavon G., Hansen K., Fazli S. and Rupp M., in Learning Invariant Representations of Molecules for Atomization Energy Prediction, Advances in Neural Information Processing Systems, ed. F. Pereira, C. J. C. Burges, L. Bottou and K. Q. Weinberger, Curran Associates, Inc., 2012, pp. 440–448.

[cit88] Gastegger M., Kauffmann C., Behler J., Marquetand P. (2016). J. Chem. Phys..

[cit89] Hastie T., Tibshirani R. and Friedman J., The Elements of Statistical Learning, Springer, New York, 2009, vol. 18, p. 764.

[cit90] Friedman J., Hastie T., Tibshirani R. (2010). J. Stat. Software.

[cit91] R Core Development Team, R: A Language and Environment for Statistical Computing. 2016.

[cit92] Larochelle H., Bengio Y., Louradour J., Lamblin P. (2009). J. Mach. Learn. Res..

[cit93] Aiello S., Kraljevic T. and Maj P., H2O: R Interface for H2O, 2015.

[cit94] Gal Y. and Ghahramani Z., Dropout as a Bayesian Approximation: Representing Model Uncertainty in Deep Learning, arXiv preprint arXiv:1506.02142, 2015.

[cit95] Srivastava N., Hinton G. E., Krizhevsky A., Sutskever I., Salakhutdinov R. (2014). J. Mach. Learn. Res..

[cit96] Hinton G. E., Srivastava N., Krizhevsky A., Sutskever I. and Salakhutdinov R. R., Improving Neural Networks by Preventing Co-Adaptation of Feature Detectors, arXiv preprint arXiv:1207.0580, 2012, pp. 1–18.

[cit97] Bengio Y., Practical Recommendations for Gradient-Based Training of Deep Architectures, in Neural Networks: Tricks of the Trade, ed. G. B. Orr, K. R. Muller and M. Gregoire, Springer, 2012, pp. 437–478.

[cit98] LeCun Y., Bottou L., Bengio Y., Haffner P. (1998). Proc. IEEE.

[cit99] Candel A., Parmar V., LeDell E. and Arora A., Deep Learning with H2O. H2O, 2015.

[cit100] Niu F., Recht B., Re C. and Wright S. J., Hogwild!: A Lock-Free Approach to Parallelizing Stochastic Gradient Descent, Advances in Neural Information Processing Systems, 2011, p. 21.

[cit101] Kingston G. B., Lambert M. F., Maier H. R. (2005). Water Resour. Res..

[cit102] Secchi P., Zio E. (2008). Ann. Nucl. Energy.

[cit103] Hansen K., Montavon G., Biegler F., Fazli S., Rupp M., Scheffler M., von Lilienfeld O. A., Tkatchenko A., Müller K.-R. (2013). J. Chem. Theory Comput..

[cit104] Zeileis A., Hornik K., Smola A., Karatzoglou A. (2004). J. Stat. Software.

[cit105] Krueger T., Panknin D., Braun M. (2015). J. Mach. Learn. Res..

[cit106] Breiman L., Friedman J., Olshen R. A. and Stone C., Classification and Regression Trees, Chapman and Hall, CRC, 1984, vol. 5, pp. 95–96.

[cit107] T. Therneau, B. Atkinson, B. Ripley, Rpart: Recursive Partitioning and Regression Trees, https://cran.r-project.org/package=rpart, accessed March 14, 2017.

[cit108] Coskun D., Jerome S. V., Friesner R. A. (2016). J. Chem. Theory Comput..

[cit109] Hughes T. F., Harvey J. N., Friesner R. A. (2012). Phys. Chem. Chem. Phys..

[cit110] Hughes T. F., Friesner R. A. (2011). J. Chem. Theory Comput..

[cit111] Jiang W., Deyonker N. J., Determan J. J., Wilson A. K. (2012). J. Phys. Chem. A.

[cit112] Bajusz D., Racz A., Heberger K. (2015). J. Cheminf..

[cit113] O'Boyle N. M., Banck M., James C. A., Morley C., Vandermeersch T., Hutchison G. R. (2011). J. Cheminf..

[cit114] The Open Babel Package Version 2.3.1, http://openbabel.org, accessed March 14, 2017.

