# Supplementary material for: Predicting electronic structure properties of transition metal complexes with neural networks
Source: Chem Sci. 2017 May 17;8(7):5137–52. doi: 10.1039/c7sc01247k (PMC6100542; doi:10.1039/c7sc01247k)
Supplement: Supplementary file 3 [file SC-008-C7SC01247K-s003.pdf]

Electronic Supplementary Material (ESI) for Chemical Science.
This journal is © The Royal Society of Chemistry 2017

Supporting Information for Predicting Electronic Structure Properties of Transition Metal Complexes with Neural Networks

Jon Paul Janet[1] and Heather Kulik *[1]

[1] *Department of Chemical Engineering, Massachusetts Institute of Technology, Cambridge, MA, USA*

Contents:

Text S1: Supporting files index . . . S3
Text S2: Estimation of τ . . . S4
Text S3: Use of Coulomb matrix fescriptor . . . S4
Text S4: Testing ANN performance in molSimplify . . . S5
Text S5: Molecular descriptors for CSD compounds . . . S5
Text S6: Dissimilarity metrics for LS/HS bond length prediction . . . S5
Table S1: Ligand properties . . . S6
Table S2: Core homoleptic ligands . . . S6
Table S3: CSD structure references . . . S7
Table S4: Relaxed tolerances for some CSD cases . . . S8
Table S5: Excluded structures: spin contamination . . . S8
Table S6: Excluded structures: geometric brekaup . . . S8
Table S7: Variable scaling constants . . . S8
Table S8: Variable Selection for splitting energy: set a . . . S9
Table S9: Variable Selection for splitting energy: set b . . . S10
Table S10: Variable Selection for splitting energy: set c . . . S11
Table S11: Variable Selection for splitting energy: set d . . . S12
Table S12: Variable Selection for splitting energy: set e . . . S13
Table S13: Variable selection for splitting energy: set f . . . S14
Table S14: Variable selection for splitting energy: set g . . . S15
Table S15: Variable selection for HF slope: set a . . . S16
Table S16: Variable selection for HF slope: set b . . . S17
Table S17: Variable selection for HF slope: set c . . . S18
Table S18: Variable selection for HF slope: set d . . . S19
Table S19: Variable selection for HF slope: set e . . . S20
Table S20: Variable selection for HF slope: set f . . . S21
Table S21: Variable selection for HF slope: set g . . . S22
Figure S1: Variable selection plots . . . S23
Table S22: Hyperparameters for different KRR and SVR models . . . S23
Figure S2: Binary ground state classification tree for heteroleptic compounds . . . S24
Figure S3: Splitting energy prediction: Mn . . . S25
Figure S4: Splitting energy prediction: Co . . . S26
Figure S5: Splitting energy prediction: Cr . . . S27

*Corresponding Author

Figure S6: Splitting energy prediction: Fe(II) and Ni(II) S28
Table S23: Splitting energy predictions and data compared S29
Figure S7: Splitting energy prediction: comparison of uncertainty S34
Figure S8: Test set error histogram for different machine learning models S34
Table S24: RMS prediction errors for splitting energy S35
Table S25: Average HF exchange sensitivity by metal and ligand connection atom . S35
Figure S9: Scatter plot of HFX sensitivity S36
Figure S10: HF slope prediction: error boxplot S36
Figure S11: HF slope prediction: Co S37
Figure S12: HF slope prediction: Cr S38
Figure S13: HF slope prediction: Fe(II) and Ni(II) S39
Figure S14: HF slope prediction: Mn S40
Table S26: HFX sensitivity predictions and data compared S41
Figure S15: HF slope prediction: comparison of uncertainty S45
Figure S16: Bond distance prediction: LS error boxplot S46
Figure S17: Bond distance prediction: comparison of LS uncertainty S46
Figure S18: Bond distance prediction: LS Co S47
Figure S19: Bond distance prediction: LS Cr S48
Figure S20: Bond distance prediction: LS Fe(II) and Ni(II) S49
Figure S21: Bond distance prediction: LS Mn S50
Table S27: LS bond distance predictions and data compared S51
Figure S22: Bond distance prediction: HS error boxplot S56
Figure S23: Bond distance prediction: comparison of HF uncertainty S56
Figure S24: Bond distance prediction: HS Co S57
Figure S25: Bond distance prediction: HS Cr S58
Figure S26: Bond distance prediction: HS Fe(II), Fe(III) and Ni(II) S59
Figure S27: Bond distance prediction: HS Mn S60
Table S28: HS bond distance predictions and data compared S61
Table S29: Error breakdown for bond distance predictions S66
Table S30: molSimplify gradients for ANN assisted structure design S66
Figure S28: Metal-ligand gradient bond projection S66
Table S31: CSD split energy prediction S67
Figure S29: CSD splitting energy errors and dissimilarity: Tanimoto/FP2 . . . . S68
Figure S30: CSD splitting energy errors and dissimilarity: Euclidean and Pearson distances S68
Table S32: LS CSD geometry prediction S69
Table S33: HS CSD geometry prediction S70
Figure S31: CSD LS geometry errors and dissimilarity comparison S71
Figure S32: CSD HS geometry errors and dissimilarity comparison S71
Figure S33: Comparison of measured CSD bond distances and DFT predicted spin states S72
Figure S34: Comparison of HFX interpolation and direct prediction S72
Table S34: CSD splitting interpolation S73

Text S1: supporting files index

In addition to results reported here, additional files are provided as follows:

provided files:

- **geometries.zip**
  - **csd_optimized_geos**
    - *test_[test number]_[% HFX]_[HS/LS].xyz*
  - **optimized_geos**
    - *ID_[metal]_[oxidation]_[spin]_[ligands][% HFX].xyz*
- **data.zip**
  - **ann_weights**
    - **split** (splitting energy)
      - *[name]_[w/b (weight/bias)][layer number].csv*
    - **slope** (HFX sensitivity)
      - (same)
    - **hs** (high-spin bond length)
      - (same)
    - **ls** (low-spin bond length)
      - (same)
- **raw_data**
  - **split** (splitting energy)
    - *raw_data.csv*<descriptors of all data>
    - *all_results.csv* <ANN pred. and data for all runs>
    - *test_results.csv* <ANN pred. and data for test runs>
  - **slope** (HFX sensitivity)
    - (same)
  - **hs** (high-spin bond length)
    - (same)
  - **ls** (low-spin bond length)
    - (same)
  - **csd_metrics** (error and distance metrics for csd)
    - error_comp_split.csv
    - error_comp_ls.csv
    - error_comp_hs.csv

Text S2: Estimation of τ

We determine a representative value of τ by maximizing the log predictive likelihood of the corresponding GP based on the training data, which is a measure of how likely the observed data are under the GP, and is approximated [6] by

$$\log p\left(\mathbf{y}\left(\mathbf{x}_n\right)|\mathbf{x}_n,\mathbf{X},\mathbf{Y}\right) \approx \log\left[\sum_{j=1}^{J} e^{-\frac{1}{2}\tau\left\|\tilde{\mathbf{y}}(\mathbf{x}_n)-\tilde{\mathbf{y}}_j(\mathbf{x}_n)\right\|_2^2}\right] - \log J - \frac{1}{2}\log 2\pi - \frac{1}{2}\log\tau^{-1} \tag{S1}$$

In the application here, we have only scalar output and we use the training data to optimize equation S1 with respect to τ numerically. We use $J = 100$ repeats, as in the network itself. The determined values of τ, based on the respective training data, are 0.4 for predicting the splitting energy, 0.07 for predicting the HF exchange sensitivity, and 10000 for the metal-ligand distances respectively. The magnitude of these numbers are close to the training errors observed, with $\sqrt{0.4^{-1}} \sim 2.5$,$0.4^{-1} \sim 1.6$, $\sqrt{0.007^{-1}} \sim 12$,$0.4^{-1} \sim 0.01$. These numbers represent the estimated inherent variance in the training data that limit of accuracy that could be expected from the trained networks.

Text S3: Use of Coulomb matrix descriptor

We compare the descriptors proposed in this work to the Coulomb matrix descriptor, which has previously [22, 9] been correlated with various molecular properties for a number of organic molecule data sets. In order to allow comparison of complexes with differing numbers of atoms, we pad all matrices with zeros to a size of of 151×151, necessitating $\mathcal{O}(10^4)$ elements per compound. We sort the rows and columns of the matrices in order to obtain indexing-invariant representations and use KRR with an exponential kernel and the matrix L_1 norm as a distance metric as in Ref [32]. The complexes in our training data range in size from 7 to 151 atoms, but have a mean and median size of 38 and 29 atoms respectively. This large skew toward smaller complexes means that most of the descriptor elements are zero, and this may make learning good model parameters difficult. For example, the L_1 distances between the sorted matrix representations of the small Fe(III)$(CN)_6$ complex and two large complexes, Fe(III)$(tbuc)_6$ and Fe(III)$(pisc)_6$ are very similar (36.85 to 36.88 where the range of distances spans $\sim 20 - 60$), despite pisc being a similar strong C-conntecting ligand and tbuc being a much weaker O-connecting ligand. We train and test on the same data as used in the other methods, but because the Coulomb matrix representation does not encode any functional-dependent information, we also provide a comparison against only B3LYP data (as opposed to varying HFX fractions).

Text S4: Testing ANN performance in molSimplify

In order to asses if the ANN can assist automated structure design, we used it to predict bond lengths instead of using the metal-ligand bond length databse integrated into our structure generation toolbox, molSimplify [13]. We selected four of the original benchmark structures where molSimplify was found not to reduce RMS gradient error relative to simple force fields. Further details about the test cases are in the original paper. We project the negative of the energy gradient on the metal and connection atoms at the initial geometry onto the vector joining them, as explained in Figure S32, and use this a measure of how close to an equilibrium bond length the initial geometry is. Note that a negative value for g means the bond would shrink in a steepest descent step, while a positive value means that it would lengthen. Large magnitudes indicate the bonds are far from equilibrium.

We achieve reductions in the absolute magnitude of g by 54-90% for bidentate cases and 7% for the monodentate case (Table S26). We note that the reductions in the metal-ligand projected gradient do not necessarily correspond to reductions in the RMS gradient, which considers contributions from all atoms. In the $\mathrm{Cr\,(bipy)_3}$ case, the RMS gradient is reduced by 30%, but it is unchanged or marginally higher in the other cases. This may be explained by considering the signs of the projected gradient, which show that the ANN universally reduces the metal-ligand bond length relative to original structure. This brings the bidentate ligands closer to the metal center and hence closer to each other, and we observe that the dominant contribution to the RMS gradient is from other atoms in the ligand structure. This could possibly be improved by training a similar ANN on the bite angles.

Text S5: Molecular descriptors for CSD compounds

The poor correlation ($R^2 = 0.1$) between the Tanimoto dissimilarity (for CSD and training ligands) and the prediction error can be understood by considering that the molecular fingerprint is insensitive to the arrangement of groups in the ligand, so two ligands might appear similar in the Tanimoto metric because they both contain certain groups, but this does not ensure that the same groups are coordinating to the metal center. The descriptors used in this work strongly suggest that the immediate metal environment determines behavior of the complex, and so this highlights a specific difficulty in translating established ideas from organic molecular similarity analysis to transition metal systems.

Text S6: Dissimilarity metrics for LS/HS bond length prediction

Using the same dissimilarity metrics that were employed to evaluate reliability of spin-state splitting, correlations between HS bond distance error and proximity to test data is smaller for both the HS bond distances ($R^2 = 0.0$, 0.1 and 0.2 for the Tanimoto similarity metric, Pearson, and Euclidean distances, respectively) and LS bond distances ($R^2 = 0$ for all metrics). However, we do observe that four of the five large (i.e., > 0.1 Å) HS bond distance errors have a minimum Euclidean distance greater than 1.0, supporting the use of this heuristic for evaluating prediction reliability. Bond length errors are generally smaller for LS states compared to HS states, with only two cases (tests 26 and 30) greater than 0.1 Å. We observe an overall correlation between the low spin bond distance prediction inaccuracy and poor splitting energy prediction, but bond lengths may still be well-predicted when spin-state splittings are not (e.g., $0.006 - -0.03$ Åerrors in LS bond distances for the cyclams).

Table S1: Ligand properties

Number	ID	Name	Denticity	Charge	Connection	$\max \delta\chi$	Bond Order	Truncated Kier
1	cl	chloride	1	1-	Cl	0	0	0
2	scn	thiocyanate	1	1-	S	0.03	2	2
3	pisc	t-butylphenyl isocyanide	1	0	C	-0.49	3	2.25
4	misc	Methyl isocyanide	1	0	C	-0.49	3	2
5	cn	cyanate	1	1-	C	-0.49	3	0
6	co	carbonyl	1	0	C	-0.89	3	0
7	ncs	isothiocyanate	1	1-	N	0.49	2	2
8	ammo	ammonia	1	0	N	0.84	1	0
9	bipy	2,2-bipyridine	2	0	N	0.49	2	4.297
10	phen	phenanthroline	2	0	N	0.49	2	3.868
11	en	ethylenediamine	2	0	N	0.84	1	3
12	porphyrin	porphyrin	4	2-	N	0.49	2	6.958
13	h2o	water	1	0	O	1.24	1	0
14	acac	acetylacetonate	2	0	O	0.89	2	3.10
15	tbuc	t-butyl catecholate	2	2-	O	0.89	1	2.52
16	ox	oxalate	2	2-	O	0.89	2	2.22

Number	ID	SMILES
1	cl	[Cl-]
2	scn	[S-]C#N
3	pisc	CC(C)(C)C1=CC=C(C=C1)[N+]#[C-]
4	misc	C[N+]#[C-]
5	cn	[C-]#N
6	co	CO
7	ncs	[N-]=C=S
9	bipy	C1ccnc(c1)c2ccccn2
10	phen	C1=CC2=CC=C3C=CC=NC3=C2N=C1
11	en	NCCN
12	porphyrin	[NH]1C2=CC3=NC(=CC4=CC=C([NH]4)C=C5C=CC(=N5)C=C1C=C2)C=C3
13	h2o	O
14	acac	CC(=O)CC(=O)C
15	tbuc	CC(C)(C)C1=CC(=C([O-])C=C1)[O-]
16	ox	[O-]C(=O)C([O-])=O

Table S2: Core homoleptic ligands

metal	oxidation	number of converged & included HFX values per ligand										
		acac	bipy	c2h3s	cn	co	en	h2o	ncs	nh3	ox	total
co	2	7	7	7	0	7	7	7	7	7	7	63
co	3	7	7	7	7	7	7	7	7	7	7	70
fe	2	7	0	7	7	7	7	7	7	7	7	63
fe	3	7	7	7	7	7	7	7	7	7	7	70
mn	2	7	7	7	0	7	7	7	7	7	5	61
mn	3	7	7	7	7	7	7	7	0	7	7	63
cr	2	7	7	7	0	7	7	7	0	7	7	56
cr	3	7	7	7	7	7	7	7	7	0	7	63
ni	2	7	7	7	5	7	7	7	7	7	7	68
total (core)		83 complexes, 577 HFX values, 1154 geometries										
total (additional)		111 complexes, 768 HFX values, 1536 geometries										
total (all)		194 complexes, 1345 HFX values, 2690 geometries										

Table S3: List of test structures from the CSD, CSD IDs, metal and oxidation state along with a short note for each and references to the original observations

number	CSD ID	Metal	Ox.	Con. Atom (Ax./Equit)	Note	Reference
1	CODZAW10	Co	2	C/N	dicyano-cobyrinate	[19]
2	DORLEB	Co	2	C/N	tetrapyrrole	[7]
3	KOCLET	Cr	3	Cl/O	tetrahydrofuran dichloride	[14]
4	KEHXEA	Co	3	Cl/N	phthalocyanine	[25]
5	ZUNSEI	Co	3	Cl/N	cylcam	[29]
6	ABZACO10	Co	2	O/O	benzoate	[5]
7	LUWPAU	Fe	2	N/N	phthalocyanine	[15]
8	TPYFEC04	Fe	2	Cl/N	tetrapyridine	[36]
9	TPPSFE10	Fe	3	S/N	substitued porphyrin	[21]
10	FUMJOO	Mn	2	O/O	large oxygen ligand	[8]
11	BUHKIA	Ni	2	O/O	sulfoxide, diphenyl-propandionate	[18]
12	SUMLET	Cr	3	C/N	cyclam	[28]
13	BOSDIX	Fe	3	O/O	bidentate oxygen	[37]
14	BMADFE10	Fe	3	S/N	cyclam	[1]
15	AHAVUB	Mn	2	N/O	dinitrobenzoate	[16]
16	DOCNFE	Fe	2	C/N	benzyldioximate	[35]
17	AFAROO	Mn	3	C/N	cyclam	[23]
18	AHDNIC	Ni	2	O/N	cyclam	[3]
19	BUPTAH	Fe	3	O/O	bidentate oxygen	[31]
20	DEDKII	Fe	2	O/N	nitrogen rings and oxygen	[11]
21	PUTHIX	Fe	2	N/N	bidentate nitrogen	[39]
22	AGIZEX	Fe	2	N/N	bidentate nitrogen	[26]
23	AKAGEY	Fe	3	N/N	large monodentate nitrogen	[34]
24	GUWYUS	Fe	3	N/N	monodentate nitrogen ligand	[20]
25	EGILOW	Fe	3	O/N	heavily substituted porphyrin	[30]
26	BINPET	Cr	3	O/N	cyclam, oxygen	[2]
27	JUSCIL	Fe	2	N/N	bidentate nitrogen	[12]
28	BULVAG	Mn	2	O/N	salen-like	[4]
29	FIXGID	Mn	3	O/N	cyclam	[24]
30	KUSKEQ	Ni	2	O/O	monodentate oxygen	[33]
31	AGUWEE	Mn	2	O/N	cyclic nitrogen	[17]
32	DMAZCO03	Co	2	N/N	imidazole	[38]
33	YUJCIQ	Cr	3	C/N	cyclam	[27]
34	ACALEW	Cr	3	N/N	monodenate nitrogen	[10]
35	AKAGAU	Cr	3	N/N	monodentate nitrogen	[34]

Table S4: Relaxed geometry optimization tolerances for some CSD structures .

number	spin	energy tolerance, au
HFX = 0.20		
2	LS	1.05×10^{-5}
2	HS	5×10^{-2}
1	LS	5×10^{-6}

Table S5: Excluded structures due to spin contamination

metal	ox	axlig	eqlig	aHF	metal	ox	axlig	eqlig	aHF
cr	3	nh3	nh3	all (7)	fe	2	tbuc	tbuc	0.30
mn	3	pisc	h2o	0.30	mn	2	ox	ox	0.25, 0.30
cr	3	tbuc	tbuc	0, 0.05					
total				5 complexes, 13 HFX values					

Table S6: Excluded structures due to geometric brekaup

metal	ox	axlig	eqlig	aHF	metal	ox	axlig	eqlig	aHF
co	3	nh3	co	0	ni	2	cn	cn	0,0.05
mn	3	ncs	ncs	all (7)	cr	2	ncs	ncs	all (7)
mn	2	cn	cn	all (7)	co	2	cn	cn	all (7)
cr	2	cn	cn	all (7)	cr	2	ncs	pisc	0
fe	3	co	scn	0,0.05	fe	2	ncs	pisc	0.25
ni	2	nh3	cn	all (7)	ni	2	scn	ox	all (7)
ni	2	h2o	h2o	all (7)					
total				13 complexes, 63 HFX values					

Table S7: List of input space descriptors and the normalization constants used in the ANNs. For a given variable x, the normalization is $\tilde{x} = \frac{x-c}{f}$.

name	unit	c	f
split energy	kcal/mol	-54.19	142.71
HFX sensitivity	kcal/mol.HFX	-174.20	161.58
ls min bond	Å	1.8146	0.6910
hs min bond	Å	1.8882	0.6956
oxidation state		2	1
a_{HF}		0	0.3
axlig charge		-2	2
eqlig charge		-2	2
axlig dent		1	1
eqlig dent		1	3
mdelen		-5.34	12.54
maxmd		-0.89	2.09
axlig bo		0	3
eqlig bo		0.00	3
axlig ki		0.00	4.29
eqlig ki		0.00	6.96

Table S8: Variable Selection for $\Delta E_{\text{HS-LS}}$: set **a**. Values are given for regularized and unregularized coefficients and MSE in $kcal^2/mol^2$

	unregularized	$\mathcal{L}_1$ regularization
(Intercept)	7.13	1.14
metalcr	-23.3	-17.3
metalfe	-8.43	-2.76
metalmn	-25.4	-19
metalni	-29.8	-23.9
ox	6.73	6
alpha	-69.4	-64.4
axligbipy	1.82	3.48
axligc2h3ns	29.2	24.4
axligcl	-9.54	-4.77
axligcn	11.7	12.5
axligco	8.79	4.07
axligen	-4.65	0
axligh2o	-10.3	-9.9
axligncs	-10.2	-4.6
axlignh3	4.86	0.21
axligox	-22	-0.42
axligphen	-1.86	0
axligpisc	1.23	0
axligporphyrin	0	0
axligscn	-1.19	0
axligtbuc	-8.8	0
eqligbipy	9.07	0
eqligc2h3n	-1.19	1.37×10^{-5}
eqligcl	-6.67	-2.43
eqligcn	8.06	10.2
eqligco	11.1	12.7
eqligen	10.1	0.284
eqligh2o	-4.94	-4.31
eqligncs	-3.36	-0.298
eqlignh3	-0.946	0
eqligox	9.04	0
eqligphen	15.8	6.99
eqligpisc	34.2	31.2
eqligporphyrin	35.3	13.2
eqligscn	-4.83	0
eqligtbuc	-4.26	-1.58
axlig charge	-9.92×10^{-2}	5.36
eqlig charge	-2.29	0
axlig dent	4.05	0
eqlig dent	-7.86	0
MSE	199	213

Table S9: Variable Selection for $\Delta E_{\text{HS-LS}}$: set **b**. Values are given for regularized and unregularized linear coefficients and MSE in kcal^2/mol^2

	unregularized	$\mathcal{L}_1$ regularization
(Intercept)	20.6	18.3
metalcr	-22.3	-17.9
metalfe	-7.03	-2.25
metalmn	-24	-19.1
metalni	-28.6	-23.6
ox	6.63	6.1
alpha	-69.5	-65
axlig charge	3.55	2
eqlig charge	-0.613	0.107
axlig dent	6.83	0.999
eqlig dent	-0.633	0
axlig connectCl	-12.9	-9.59
axlig connectN	-10.2	-6.14
axlig connectO	-17.1	-12.4
axlig connectS	-4.6	-2.39
eqlig connectCl	-23.1	-21.2
eqlig connectN	-18.2	-16.1
eqlig connectO	-23.6	-21.5
eqlig connectS	-21.6	-18.8
axlig natoms	-0.235	0
eqlig natoms	0.525	0.414
MSE	218	227

Table S10: Variable Selection for $\Delta E_{\text{HS-LS}}$: set **c**. Values are given for regularized and unregularized linear coefficients and MSE in kcal^2/mol^2

	unregularized	$\mathcal{L}_1$ regularization
(Intercept)	26.4	13.6
metalcr	-22.5	-18.6
metalfe	-7.1	-3.18
metalmn	-24	-20
metalni	-28.9	-24.7
ox	6.6	6.17
alpha	-69.4	-66.1
axlig charge	3.57	2.34
eqlig charge	-1.22	0.702
axlig dent	8.6	0
eqlig dent	-0.841	0
axlig connectCl	-17.3	-7.43
axlig connectN	-19	-0.818
axlig connectO	-28.9	-4.73
axlig connectS	-9.04	0
eqlig connectCl	-26	-16.8
eqlig connectN	-24.6	-5.78
eqlig connectO	-31.5	-8.28
eqlig connectS	-24.6	-14
axlig natoms	-0.299	0
eqlig natoms	0.568	0.399
$\Sigma\ \Delta\chi$	-0.203	-1.95
min$\Delta\chi$	7.08	0
max$\Delta\chi$	5.43	-1.33
MSE	216	230

Table S11: Variable Selection for $\Delta E_{\text{HS-LS}}$: set **d**. Values are given for regularized and unregularized linear coefficients and MSE in $\text{kcal}^2/\text{mol}^2$

	unregularized	$\mathcal{L}_1$ regularization
(Intercept)	26.1	14.8
metalcr	-22.5	-18.9
metalfe	-7.1	-3.49
metalmn	-24.1	-20.3
metalni	-28.8	-25.1
ox	6.58	6.2
alpha	-69.4	-66.4
axlig charge	3.69	2.39
eqlig charge	-1.3	0.595
axlig dent	7.92	0.115
eqlig dent	-0.48	0
axlig connectCl	-15.2	-7.78
axlig connectN	-15.2	-1.6
axlig connectO	-23.9	-5.75
axlig connectS	-7.03	0
eqlig connectCl	-27.3	-17.8
eqlig connectN	-26.9	-7.23
eqlig connectO	-34.5	-10.1
eqlig connectS	-25.8	-15.1
axlig natoms	-0.277	0
eqlig natoms	0.556	0.407
max$\Delta\chi$	-1.79	-1.47
$\Sigma\ \Delta\chi$	2	-1.68
MSE	216	228

Table S12: Variable selection for $\Delta E_{\text{HS-LS}}$: set **e**. Values are given for regularized and unregularized linear coefficients and MSE in kcal^2/mol^2

	unregularized	$\mathcal{L}_1$ regularization
(Intercept)	32.7	19.1
metalcr	-22.2	-19.8
metalfe	-7.72	-4.74
metalmn	-24.1	-21.5
metalni	-29.6	-26.4
ox	6.6	6.38
alpha	-69.3	-67.3
axlig charge	5.12	2.83
eqlig charge	-3.68	0
axlig dent	6.49	1.89
eqlig dent	0.769	0.283
axlig connectCl	-16.7	-10.8
axlig connectN	-13.6	-4.97
axlig connectO	-18.1	-8.77
axlig connectS	0.368	0
eqlig connectCl	-26.5	-19.7
eqlig connectN	-30.7	-14
eqlig connectO	-46.1	-20.4
eqlig connectS	-37.8	-21.2
axlig natoms	-4.3×10^{-2}	0
eqlig natoms	0.32	0.378
axlig bo	3.64	1.39
eqlig bo	-5.96	-1.78
$\Sigma\ \Delta\chi$	2.25	-0.591
max$\Delta\chi$	-2.41	-1.46
MSE	213	221

Table S13: Variable selection for $\Delta E_{\text{HS-LS}}$: set **f**. Values are given for regularized and unregularized linear coefficients and MSE in $kcal^2/mol^2$

	unregularized	$\mathcal{L}_1$ regularization
(Intercept)	27.8	17.8
metalcr	-22.4	-19.5
metalfe	-6.83	-3.71
metalmn	-24.3	-21.1
metalni	-29.9	-26
ox	6.69	6.27
alpha	-69.4	-66.9
axlig charge	2.38	2.11
eqlig charge	-0.265	0.992
axlig dent	11.3	2.18
eqlig dent	-2.95	0.846
axlig connectCl	-17.1	-9.14
axlig connectN	-14.4	-4.04
axlig connectO	-25.7	-9.6
axlig connectS	-1.94	-0.457
eqlig connectCl	-28.6	-21.5
eqlig connectN	-33.9	-14.6
eqlig connectO	-39.4	-17.7
eqlig connectS	-35.4	-22
axlig ki	-2.65	-0.343
eqlig ki	4	1.78
max$\Delta\chi$	-1.19	-0.721
Σ $\Delta\chi$	2.48	-0.868
MSE	218	227

Table S14: Variable selection for $\Delta E_{\text{HS-LS}}$: set **g**. Values are given for regularized and unregularized linear coefficients and MSE in $kcal^2/mol^2$

	unregularized	$\mathcal{L}_1$ regularization
(Intercept)	35.2	21.6
metalcr	-22.2	-19.6
metalfe	-7.5	-4.28
metalmn	-24.4	-21.5
metalni	-30.1	-26.5
ox	6.63	6.32
alpha	-69.3	-67.1
axlig charge	4.04	2.75
eqlig charge	-2.84	0
axlig dent	10.1	2.19
eqlig dent	0.186	2.29
axlig connectCl	-17.7	-10.4
axlig connectN	-13.9	-4.73
axlig connectO	-22	-8.81
axlig connectS	0.214	0
eqlig connectCl	-27.7	-21.3
eqlig connectN	-34	-16.3
eqlig connectO	-47.9	-23.7
eqlig connectS	-41.9	-25.8
axlig bo	2.05	1.24
eqlig bo	-5.52	-2.71
axlig ki	-1.66	0
eqlig ki	1.97	0.925
max$\Delta\chi$	-2.08	-1.09
Σ $\Delta\chi$	2.41	-0.622
MSE	215	223

Table S15: Variable selection for $\frac{\partial \Delta E_{H\text{-}L}}{\partial a_{HF}}$: set **a**. Values are given for regularized and unregularized coefficients and MSE in HF kcal^2/mol^{2}HFX^2

	unregularized	$\mathcal{L}_1$ regularization
(Intercept)	-167	-167
metalcr	21.7	21.2
metalfe	-26.3	-26.3
metalmn	-15.2	-15.2
metalni	14.9	14.2
ox	28.2	27.9
eqligbipy	-20.7	-16.6
eqligc2h3ns	-30.5	-30.1
eqligcl	-2.95	-0.898
eqligcn	-6.33	-4.87
eqligco	-18.4	-14.8
eqligen	-4.02	-1.31
eqligh2o	8.22	11.8
eqligncs	1.03	2.08
eqlignh3	4.88	7.82
eqligox	-0.272	-1.45
eqligphen	-1.28	0
eqligpisc	-31.6	-27.5
eqligporphyrin	8.4	1.34
eqligscn	6.26	6.82
eqligtbuc	10.8	7.53
axligbipy	15.5	10.2
axligc2h3ns	-1.39	-0.326
axligcl	6.15	0.329
axligcn	-9.2	-14
axligco	-21.8	-24.1
axligen	17.4	13.5
axligh2o	15	11.8
axligncs	7.92	2.13
axlignh3	4.47	2.07
axligox	11	0.491
axligphen	-10.2	-11.8
axligpisc	-16.3	-18.9
axligporphyrin	0	0
axligscn	2.97	-1.65
axligtbuc	8.32	0
eqlig charge	2.31	0
axlig charge	-1.12	-4.04
eqlig dent	-1.75	0
axlig dent	-8.15×10^{-2}	0
alpha	5.72	5.42
MSE	422	423

Table S16: Variable selection for $\frac{\partial \Delta \mathrm{E}_{\mathrm{H\text{-}L}}}{\partial a_{\mathrm{HF}}}$: set **b**. Values are given for regularized and unregularized linear coefficients and MSE in HF kcal^2/mol^{2}HFX^2

	unregularized	$\mathcal{L}_1$ regularization
(Intercept)	-189	-186
metalcr	21.9	21.7
metalfe	-26.9	-26.7
metalmn	-16.3	-16.1
metalni	13.8	13.3
ox	28.6	28.2
eqlig charge	-0.266	-0.359
axlig charge	-3.79	-3.49
eqlig dent	0.179	0
axlig dent	-7.42	-6.37
eqlig natoms	-0.47	-0.452
axlig natoms	2.13×10^{-2}	0
eqlig connectCl	10.5	9.61
eqlig connectN	17.6	17
eqlig connectO	19.3	18.9
eqlig connectS	20.1	19.2
axlig connectCl	18.6	18.1
axlig connectN	24.5	23.8
axlig connectO	29.7	28.8
axlig connectS	18.1	17.7
alpha	4.89	4.58
MSE	472	472

Table S17: Variable selection for $\frac{\partial \Delta \text{EH-L}}{\partial a_{\text{HF}}}$: set **c**. Values are given for regularized and unregularized linear coefficients and MSE in HF kcal^2/mol^{2}HFX^2

	unregularized	$\mathcal{L}_1$ regularization
(Intercept)	-170	-167
(Intercept)	0	0
metalcr	20.8	20.9
metalfe	-27.6	-27.2
metalmn	-17.1	-16.8
metalni	13.1	12.8
ox	28.6	28.4
eqlig charge	-2.06	-1.99
axlig charge	-3.98	-3.44
eqlig dent	2.38	0
axlig dent	0.717	0
eqlig natoms	-0.385	-0.325
axlig natoms	-0.144	-0.161
eqlig connectCl	-2.56	0.619
eqlig connectN	-9.37	-2.65
eqlig connectO	-13.9	-5.88
eqlig connectS	6.76	9.66
axlig connectCl	6.83	7.52
axlig connectN	-0.514	2.22
axlig connectO	-4.21	0
axlig connectS	5.29	6.36
alpha	4.21	3.69
$\Sigma\Delta\chi$	1.76	1.02
max$\Delta\chi$	14.4	13.1
min$\Delta\chi$	14.2	13
MSE	449	450

Table S18: Variable selection for $\frac{\partial \Delta E_{\text{H-L}}}{\partial a_{\text{HF}}}$: set **d**. Values are given for regularized and unregularized linear coefficients and MSE in HF kcal^2/mol^{2}HFX^2

	unregularized	$\mathcal{L}_1$ regularization
(Intercept)	-168	-166
metalcr	20.8	20.9
metalfe	-27.4	-27.2
metalmn	-17.2	-17
metalni	13.2	12.8
ox	28.6	28.4
eqlig charge	-2.24	-2.05
axlig charge	-3.61	-3.24
eqlig dent	0.574	-0.291
axlig dent	-1.65	-1.14
eqlig natoms	-0.388	-0.347
axlig natoms	-0.108	-0.147
eqlig connectCl	-4.31	0
eqlig connectN	-12.4	-6.75
eqlig connectO	-18.8	-11.6
eqlig connectS	5.36	8.2
axlig connectCl	10.9	10.6
axlig connectN	7.03	8.26
axlig connectO	5.61	7.47
axlig connectS	9.34	9.41
alpha	4.17	3.55
$\Sigma\Delta\chi$	5.86	4.97
max$\Delta\chi$	0.542	0.725
MSE	451	452

Table S19: Variable selection for $\frac{\partial \Delta E_{\text{H-L}}}{\partial a_{\text{HF}}}$: set **e**. Values are given for regularized and unregularized linear coefficients and MSE in HF kcal^2/mol^{2}HFX^2

	unregularized	$\mathcal{L}_1$ regularization
(Intercept)	-190	-187
metalcr	20.7	20.6
metalfe	-26.5	-26.4
metalmn	-16.9	-16.8
metalni	13.9	13.5
ox	28.6	28.3
eqlig charge	0.243	0
axlig charge	-4.29	-3.67
eqlig dent	0.362	-1.06
axlig dent	-3.11	-1.84
eqlig natoms	-0.252	-0.172
axlig natoms	-6.34$\times 10^{-2}$	-0.159
eqlig connectCl	-4.69	-0.473
eqlig connectN	-9.57	-3.89
eqlig connectO	-8.03	-0.743
eqlig connectS	16.6	19.5
axlig connectCl	12.8	12.3
axlig connectN	8.55	9.11
axlig connectO	7.62	8.08
axlig connectS	10.1	9.26
alpha	5.12	4.43
axlig bo	5×10^{-1}	0
eqlig bo	5.57	5.77
$\Sigma\Delta\chi$	5.62	4.81
max$\Delta\chi$	1.4	1.45
MSE	444	445

Table S20: Variable selection for $\frac{\partial \Delta \mathrm{E}_{\text{H-L}}}{\partial a_{\mathrm{HF}}}$: set **f**. Values are given for regularized and unregularized linear coefficients and MSE in HF kcal^2/mol^{2}HFX^2

	unregularized	$\mathcal{L}_1$ regularization
(Intercept)	-192	-122
metalcr	20.9	17.9
metalfe	-27.7	-21.6
metalmn	-16.8	-10.8
metalni	14.3	1.98
ox	28.5	19.8
eqlig charge	-1.58	-0.317
axlig charge	-3.9	-2.2
eqlig dent	9.74	0
axlig dent	-0.587	0
eqlig connectCl	-1.05	0
eqlig connectN	-0.537	0
eqlig connectO	-5.67	0
eqlig connectS	16.9	0
axlig connectCl	15.5	0
axlig connectN	13.8	0
axlig connectO	15.2	0
axlig connectS	11.4	0
n alpha	5.18	0
axlig ki	-0.177	0
eqlig ki	-4.11	0
max$\Delta\chi$	0.434	0
$\Sigma\Delta\chi$	3.91	4.27
MSE	453	553

Table S21: Variable selection for $\frac{\partial \Delta E_{\text{H-L}}}{\partial a_{\text{HF}}}$: set **g**. Values are given for regularized and unregularized linear coefficients and MSE in HF kcal^2/mol^{2}HFX^2

	unregularized	$\mathcal{L}_1$ regularization
(Intercept)	-192	-187
metalcr	20.9	20.8
metalfe	-27.7	-27.5
metalmn	-16.8	-16.6
metalni	14.3	13.8
ox	28.5	28.2
eqlig charge	-1.58	-1.68
axlig charge	-3.9	-3.66
eqlig dent	9.74	7.56
axlig dent	-0.587	0
eqlig connectCl	-1.05	0
eqlig connectN	-0.537	0
eqlig connectO	-5.67	-3.96
eqlig connectS	16.9	15.7
axlig connectCl	15.5	14.3
axlig connectN	13.8	13.3
axlig connectO	15.2	14.4
axlig connectS	11.4	11.3
alpha	5.18	4.64
axlig ki	-0.177	-0.364
eqlig ki	-4.11	-3.37
max$\Delta\chi$	0.434	0.522
$\Sigma\Delta\chi$	3.91	3.83
MSE	453	454

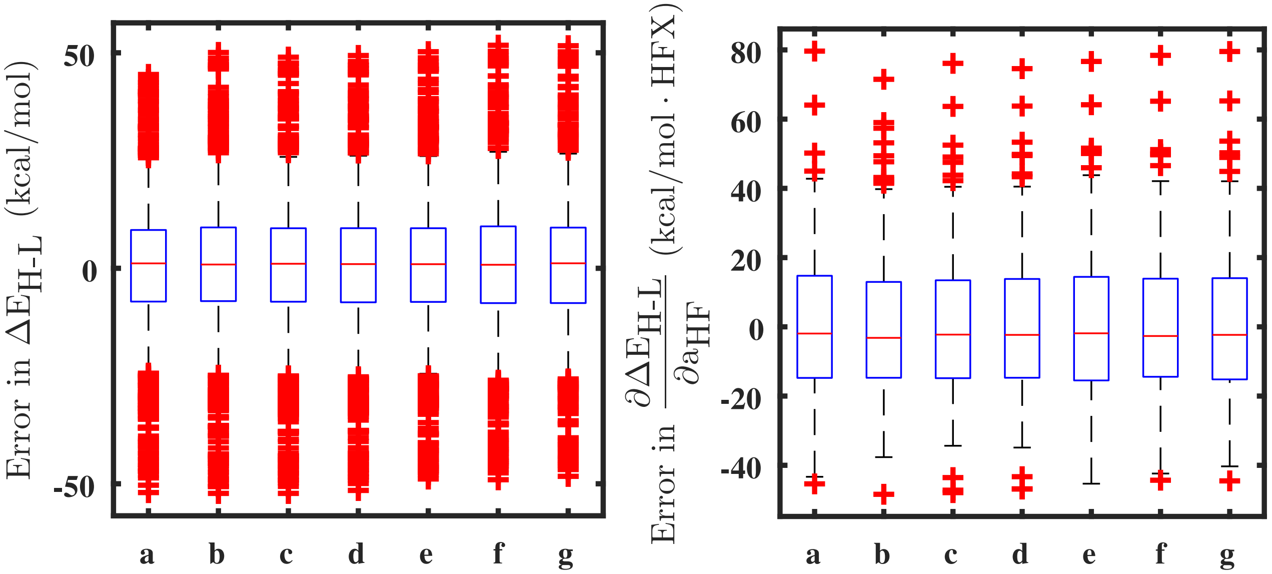

Figure S1: Comparison of errors for different descriptor sets for a regularized linear effects model predicting $\Delta E_{\text{H-L}}$ in kcal/mol (left) and $\frac{\partial \Delta E_{\text{H-L}}}{\partial a_{\text{HF}}}$ in kcal/molHFX (right). Set **a** includes the metal properties and full ligand identity and number of atoms. Set **b** replaces ligand identity with the identity of connection atom only, while set c adds information from the sum, maximum and minimum ligand $\delta\chi$ to set **b**. Set d is the same as set c but excludes the minimum $\delta\chi$. Set **e** adds in bond order information with an MSE, while set f replaces the ligand size metric with our truncated index. Set **g** represents our final set, and includes the same descriptors from **f** and adds bond order information.

Table S22: Optimal hyperparameter selection for KRR and SVR models found via a grid search and 10-fold cross-validation.Paremteres were selected usnig a cartesian grid search in $[10^{-7}, 10^{4}]$ for the regularization weights, $[10^{-4}, 10^{3}]$ for the exponenial kernel correlation length and $[0.1, 0.9]$ for ν.

	σ (kernel lengthscale)	λ/C (KRR/SVR) (regularization weight)	ν (SV fraction)
KRR (set **g**)	1	10^{-4}	
SVR (set **g**)	1	100	0.75
KRR (sorted Coulomb Matrix, B3LYP)			
KRR (sorted Coulomb Matrix, B3LYP)	316	0.01	

Figure S2: Binary ground state classification tree for heteroleptic compounds. M indicates metal identity, l ligand connection atom, o oxidation state, a the fraction of HF exchange and me the sum of $\delta\chi$ values across ligands. The first line in each leaf node is the percent of elements in that leaf that have the indicated ground state, and the second line indicates the percentage of the total heteroleptic population in each leaf node. Dashed blue arrows indicate yes, solid red arrows indicate no.

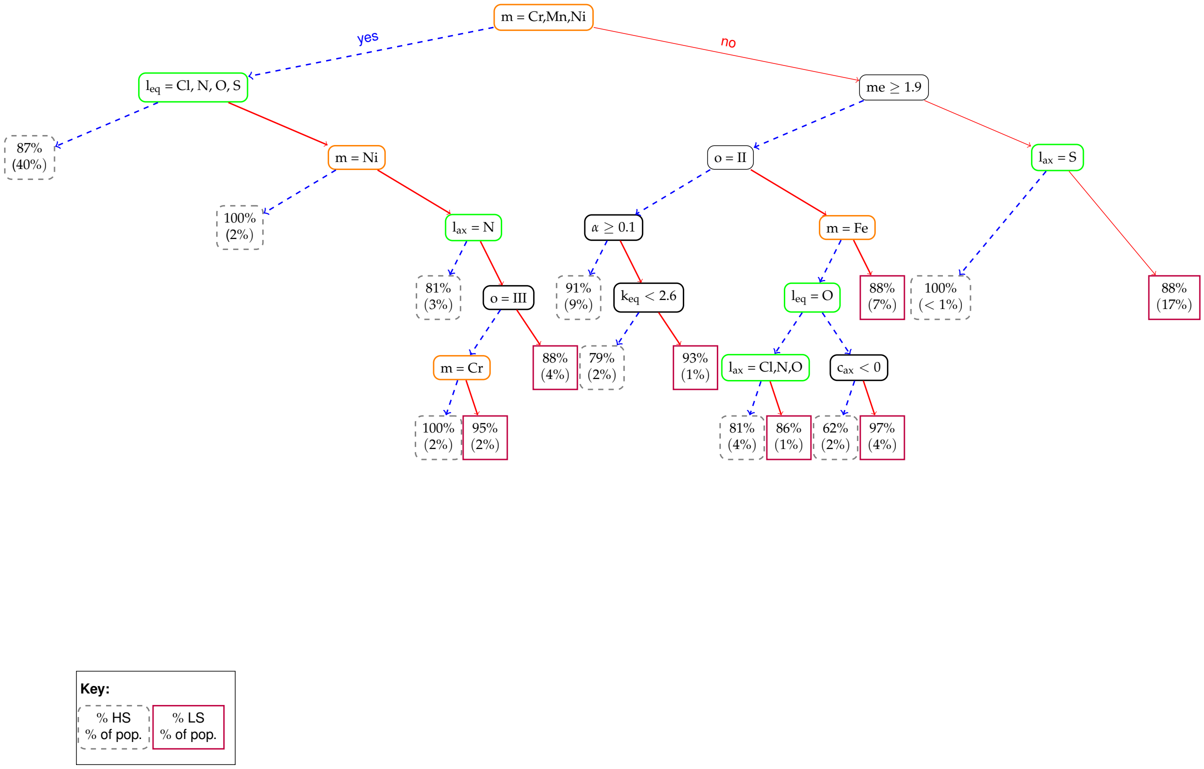

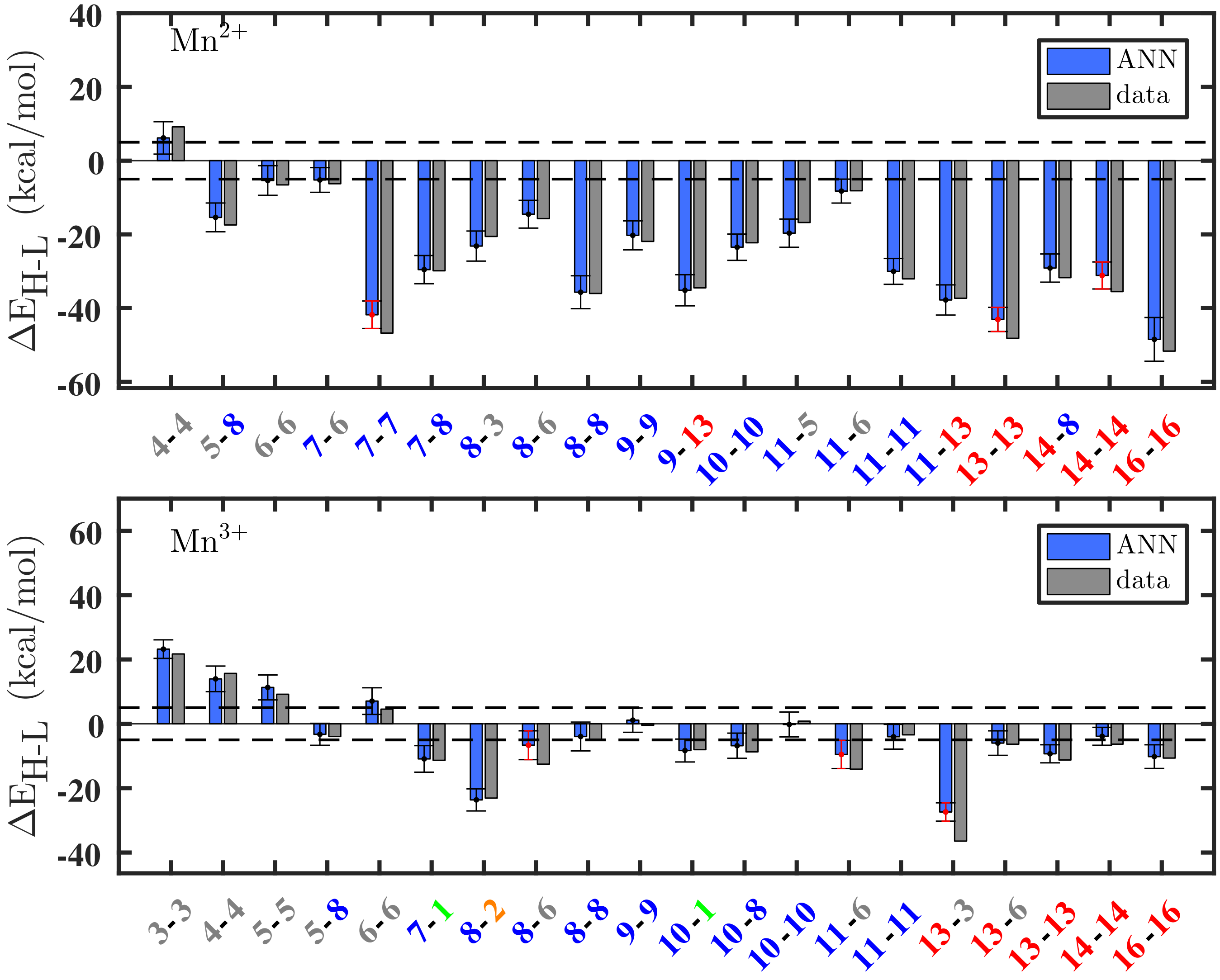

Figure S3: Model predictions of $\Delta E_{H\text{-}L}$ and data for Mn using an ANN. The ligands are described by two numbers indicating the equatorial first and then the axial, color coded by ligand identity (green for halogen, gray for carbon, blue for nitrogen, and red for oxygen). The error bars represent an estimated 1 standard deviation from the mean prediction.

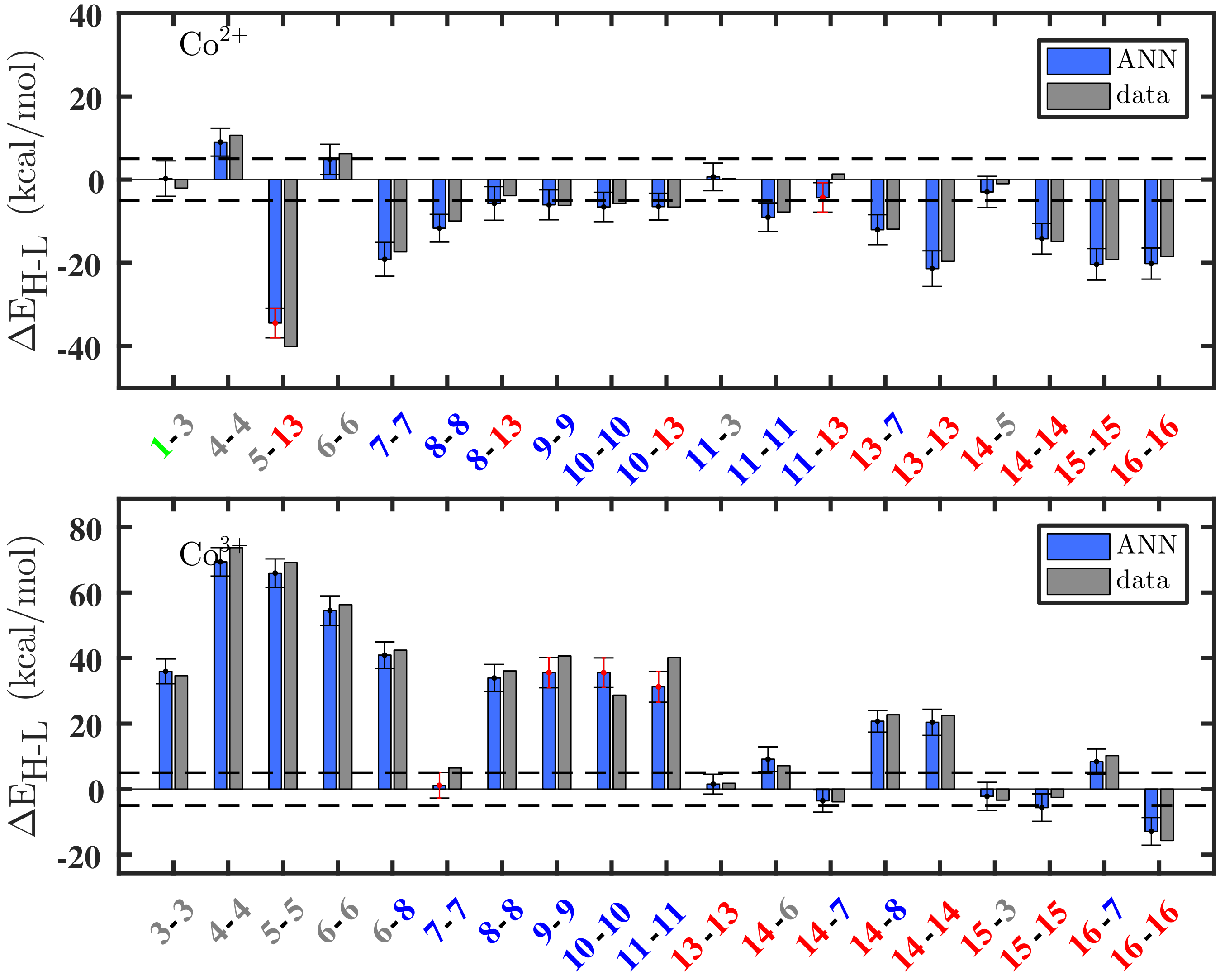

Figure S4: Model predictions of $\Delta E_{HS\text{-}LS}$ and data for Co using an ANN. The ligands are described by two numbers indicating the equatorial first and then the axial, color coded by ligand identity (green for halogen, gray for carbon, blue for nitrogen, and red for oxygen). The error bars represent an estimated 1 standard deviation from the mean prediction.

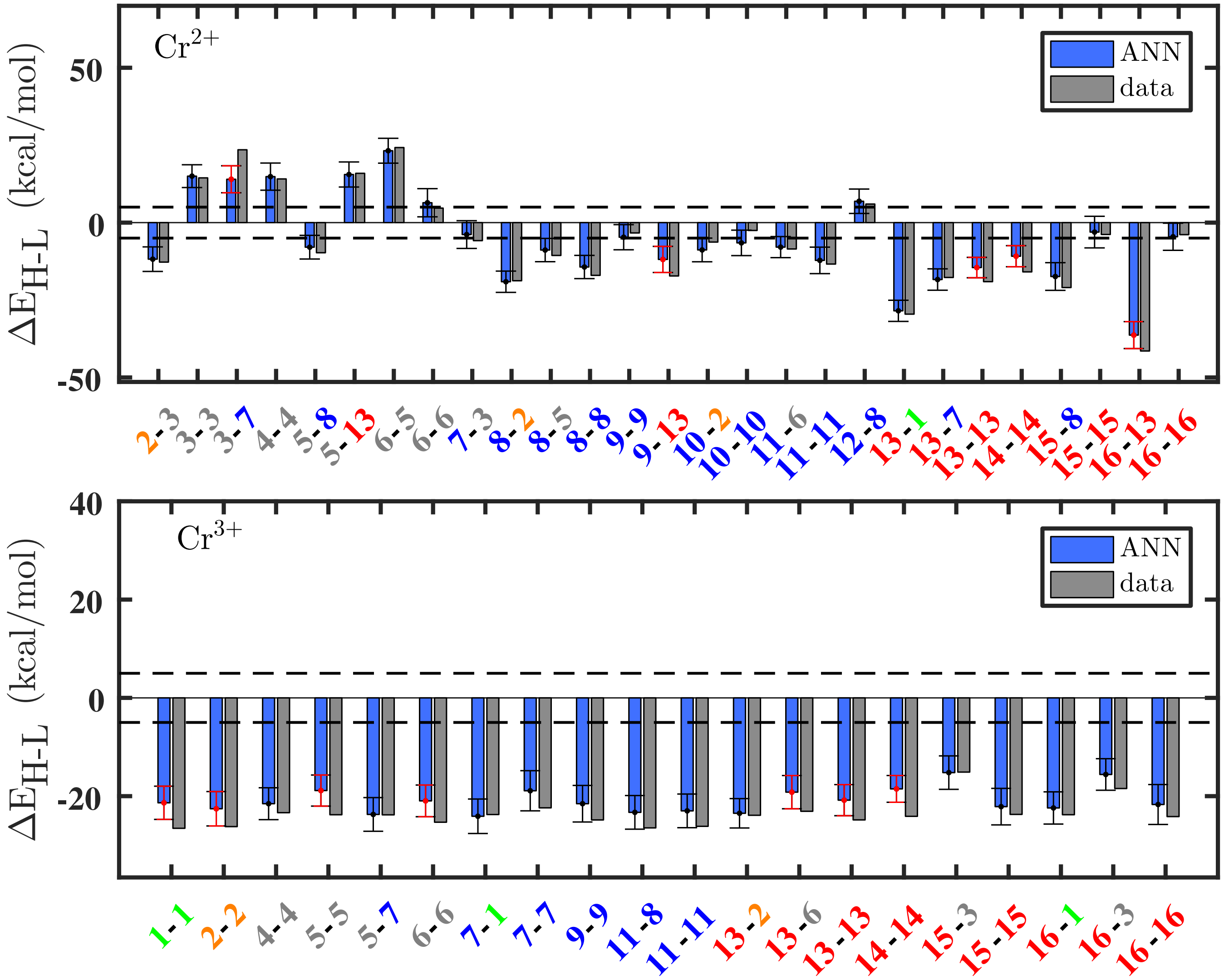

Figure S5: Model predictions of $\Delta E_{H\text{-}L}$ and data for Cr using an ANN. The ligands are described by two numbers indicating the equatorial first and then the axial, color coded by ligand identity (green for halogen, gray for carbon, blue for nitrogen, and red for oxygen). The error bars represent an estimated 1 standard deviation from the mean prediction.

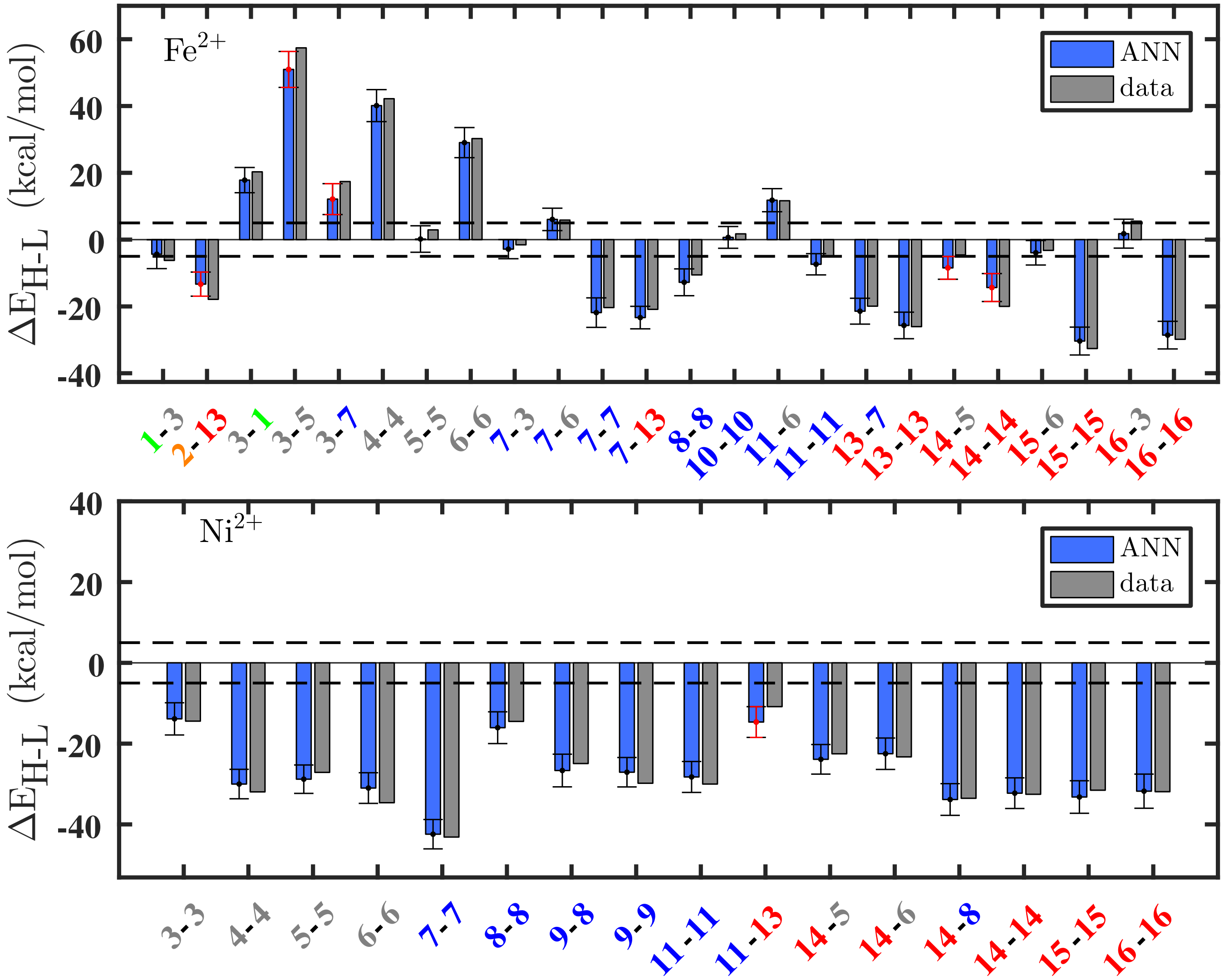

Figure S6: Model predictions of $\Delta E_{H\text{-}L}$ and data for Fe(II) (top) and Ni(II) (bottom) using an ANN. The ligands are described by two numbers indicating the equatorial first and then the axial, color coded by ligand identity (green for halogen, gray for carbon, blue for nitrogen, and red for oxygen). The error bars represent an estimated 1 standard deviation from the mean prediction.

Table S23: Splitting energy predictions and data in kcal/mol, compared for the full set of complexes. Each complex is named [metal oxidation axial ligand eq equatorial ligand]. For each complex, the first row has the data obtained from DFT using varying fractions of exact exchange (a_{HF}) and the second row has the ANN predictions

		a_{HF}						
name	type	0.00	0.05	0.1	0.15	0.20	0.25	0.30
cr 2 eq h2o ax ncs	data	-12.6	-14.5	-15.7	-16.7	-17.7	-18.6	-19.5
	pred	-8.37	-13.1	-15.4	-16.9	-18.4	-19.9	-21.8
cr 2 eq porphyrin ax nh3	data	13.8	11.9	9.98	8.13	5.99	5.55	5.32
	pred	11.9	11	10	8.58	6.9	5.31	3.81
cr 2 eq scn ax pisc	data	5.14	0.567	-4	-8.44	-12.7	-16.9	-20.8
	pred	7.04	2.99	-2.5	-7.76	-11.8	-15.1	-18.3
cr 2 eq ncs ax pisc	data	11.3	8.27	3.43	-1.33	-5.82	-10	-13.9
	pred	8.4	6.51	3.61	-0.0798	-3.86	-6.58	-8.45
cr 2 eq cn ax nh3	data	5.23	-1.62	-4.91	-7.48	-9.73	-10.4	-12.5
	pred	4.81	0.785	-2.55	-5.35	-7.93	-10.2	-12
cr 2 eq tbuc ax tbuc	data	7.45	4.37	1.46	-1.26	-3.83	-6.25	-8.56
	pred	4.1	3.19	1.94	-0.0318	-3.06	-6.91	-10.9
cr 2 eq phen ax scn	data	10.2	6.45	2.19	-2.07	-6.28	-10.4	-14.4
	pred	8.11	4.4	0.0103	-4.36	-8.82	-13.5	-18.4
cr 2 eq en ax co	data	9.12	4.19	-0.386	-4.62	-8.51	-12.1	-15.3
	pred	10.5	3.83	-1.79	-5.36	-7.91	-11.2	-13.9
cr 2 eq bipy ax bipy	data	10.2	7.27	3.64	0.0782	-3.33	-6.49	-9.29
	pred	9.12	3.51	-0.407	-2.82	-4.71	-6.48	-8.2
cr 2 eq en ax en	data	-7.2	-8.91	-10.5	-12	-13.4	-14.7	-16
	pred	-3.58	-6.9	-9.09	-10.8	-12.2	-13.2	-13.9
cr 2 eq co ax cn	data	36.5	39.7	34.4	29.3	24.2	19.3	14.6
	pred	36.4	34.4	31.7	27.9	23.2	18.4	14
cr 2 eq pisc ax pisc	data	33.2	28	23.2	18.7	14.4	10.5	6.85
	pred	29.7	26.7	23.1	18.8	15	12	9.41
cr 2 eq ox ax h2o	data	-29.3	-32.5	-35.5	-38.5	-41.4	-44.3	-47
	pred	-27.6	-30.2	-32.8	-34.9	-36.3	-37.5	-38.4
cr 2 eq h2o ax cl	data	-26.6	-27.4	-27.6	-28.3	-29.6	-30.2	-30.7
	pred	-23.8	-25.2	-26.4	-27.5	-28.5	-29.4	-30.3
cr 2 eq nh3 ax scn	data	-12	-14	-15.7	-17.3	-18.8	-20.1	-21.4
	pred	-11	-12.9	-14.7	-16.7	-19.1	-21.9	-24.9
cr 2 eq ox ax ox	data	8.53	5.44	2.32	-0.786	-3.89	-6.92	-9.9
	pred	4.47	2.89	0.835	-1.65	-4.57	-7.76	-10.8
cr 2 eq h2o ax h2o	data	-14.5	-11.8	-13.5	-15.1	-19.1	-20	-21
	pred	-8.05	-9.85	-11.4	-13	-14.5	-16.1	-18.2
cr 2 eq cn ax h2o	data	19.2	18.5	17.8	16.9	15.9	14.9	13.8
	pred	16.6	16.4	16.3	16	15.5	14.5	11.8
cr 2 eq pisc ax ncs	data	-	37.8	32.9	28.1	23.5	19.1	9.71
	pred		30.5	25.5	20.1	14	7.89	0.0285
cr 2 eq acac ax acac	data	3.66	-0.746	-4.83	-8.78	-15.9	-18.4	-20.4
	pred	1.43	0.00453	-2.61	-6.79	-10.8	-14.2	-17.6
cr 2 eq bipy ax h2o	data	-6.42	-9.51	-12.3	-14.9	-17.2	-19.3	-21.1
	pred	-5.37	-6.85	-8.14	-9.69	-11.9	-14.8	-17.7
cr 2 eq nh3 ax nh3	data	-10.2	-11.6	-13	-14.3	-17	-18.1	-17.8
	pred	-6.81	-9.52	-11.4	-12.8	-14.3	-16.2	-19.1
cr 2 eq co ax co	data	22.6	17.7	13.2	8.78	4.68	0.866	-2.66
	pred	21.1	17	13	9.44	6.42	3.68	0.934
cr 2 eq c2h3ns ax c2h3ns	data	31	26.5	22.2	18.1	14.1	10.4	6.8
	pred	31.3	28.4	24.5	19.9	14.9	9.81	4.93
cr 2 eq phen ax phen	data	12.2	8.25	4.51	0.864	-2.53	-5.63	-8.32
	pred	5.99	0.882	-2.44	-4.63	-6.54	-8.34	-10.1
cr 2 eq tbuc ax nh3	data	-11.8	-14.1	-16.5	-18.7	-21	-23.1	-25.2
	pred	-11.2	-12.5	-13.7	-15.2	-17.4	-20.1	-22.6
cr 2 eq nh3 ax cn	data	-2.14	-4.47	-6.65	-8.69	-10.6	-12.4	-15.2
	pred	0.612	-2.48	-5.21	-7.28	-8.86	-10.9	-14
cr 3 eq c2h3ns ax c2h3ns	data	-18.7	-20	-21.2	-22.3	-23.4	-24.3	-25.3
	pred	-15.5	-17.7	-19.1	-20.3	-21.5	-22.5	-23.1
cr 3 eq acac ax acac	data	-20	-21.2	-22.2	-23.2	-24.1	-24.9	-25.8
	pred	-10.1	-14.4	-16.8	-17.8	-18.5	-19.2	-20
cr 3 eq h2o ax scn	data	-19.2	-20.5	-21.6	-22.8	-23.9	-25.5	-26.5
	pred	-17.3	-20.2	-21.7	-22.7	-23.5	-24	-24.4
cr 3 eq en ax nh3	data	-23.9	-24.6	-25.2	-25.8	-26.5	-27.1	-27.7
	pred	-20.4	-21.7	-22.4	-22.9	-23.3	-23.7	-24.2
cr 3 eq scn ax scn	data	-21.4	-22.8	-24	-25.2	-26.2	-27.2	-28.1
	pred	-17.3	-19.3	-20.3	-21.3	-22.6	-24.4	-26.4
cr 3 eq en ax en	data	-23.2	-23.9	-24.7	-25.4	-26.1	-26.8	-27.5
	pred	-22.2	-22.5	-22.7	-22.9	-23	-23.2	-23.4
cr 3 eq cn ax cn	data	-19.5	-20.7	-21.8	-22.8	-23.8	-24.7	-25.6
	pred	-13.8	-15.1	-16.3	-17.4	-18.9	-20.5	-22
cr 3 eq cn ax ncs	data	-19.2	-20.5	-21.7	-22.8	-23.8	-24.8	-25.7
	pred	-13.6	-17	-19.8	-21.8	-23.7	-26.3	-28.5
cr 3 eq tbuc ax tbuc	data	-	-	-21.9	-22.8	-23.7	-24.6	-25.4
	pred			-21.3	-21.8	-22.1	-22.6	-23.4
cr 3 eq ncs ax ncs	data	-17.2	-18.6	-20	-21.2	-22.4	-23.5	-24.5
	pred	-14.6	-15.5	-16.5	-17.6	-18.9	-20.2	-21.4
cr 3 eq ox ax ox	data	-20.7	-21.6	-22.5	-23.3	-24.2	-24.9	-25.7
	pred	-18	-19.2	-20.3	-21.2	-21.7	-22	-22.5
cr 3 eq cl ax cl	data	-12.5	-13.9	-15.3	-25.7	-26.5	-27.4	-28.1
	pred	-12.7	-14	-15.8	-18.5	-21.4	-23.4	-24.7
cr 3 eq co ax co	data	-21.5	-22.6	-23.5	-24.4	-25.3	-26.1	-26.9

		a_{HF}						
name	type	0.00	0.05	0.1	0.15	0.20	0.25	0.30
	pred	-16.7	-18.6	-19.6	-20.4	-21	-21.5	-21.9
cr 3 eq h2o ax co	data	-18.2	-19.6	-20.8	-22	-23.1	-24.1	-25.1
	pred	-15.6	-16.8	-17.7	-18.5	-19.2	-19.8	-20.3
cr 3 eq bipy ax bipy	data	-21.4	-22.4	-23.3	-24.1	-24.8	-25.6	-26.3
	pred	-15.9	-18.7	-20.2	-21	-21.5	-21.9	-22.2
cr 3 eq ncs ax cl	data	-18.8	-20.5	-21.6	-22.7	-23.7	-24.7	-25.7
	pred	-19.7	-21.3	-22.6	-23.5	-24.1	-24.5	-24.9
cr 3 eq tbuc ax pisc	data	-5.9	-9.35	-12.8	-15.9	-15.1	-20.3	-21.9
	pred	-8.48	-10.2	-12	-13.7	-15.2	-16.6	-17.9
cr 3 eq ox ax cl	data	-20.4	-21.3	-22.2	-23	-23.8	-24.5	-25.3
	pred	-16.9	-18.4	-19.9	-21.4	-22.4	-23.1	-23.5
cr 3 eq h2o ax h2o	data	-21.2	-22.1	-23.1	-24	-24.8	-25.7	-26.5
	pred	-17.9	-18.7	-19.4	-20.1	-20.8	-21.5	-22.1
cr 3 eq ox ax pisc	data	-12.4	-12.1	-14.4	-16.9	-18.4	-19.8	-21
	pred	-7.49	-10.1	-12.1	-13.8	-15.6	-17	-18.2
mn 2 eq acac ax nh3	data	-4.7	-12.8	-20.1	-26.6	-31.7	-36.3	-40.3
	pred	-5.43	-11.8	-19.4	-24.8	-29.1	-32.9	-37.1
mn 2 eq nh3 ax nh3	data	-21.4	-25.4	-28.9	-32.6	-36	-39.3	-42.5
	pred	-16.6	-21.8	-27.2	-31.9	-35.7	-38.9	-40.9
mn 2 eq co ax co	data	29.5	19.7	10.5	1.68	-6.57	-14.3	-21.4
	pred	24	18.8	11.8	2.64	-5.38	-10.2	-14.2
mn 2 eq cn ax nh3	data	-3.47	0.938	-5.6	-11.7	-17.4	-22.7	-27.6
	pred	0.733	-3	-6.64	-11.1	-15.4	-19.2	-23.5
mn 2 eq en ax en	data	-15.7	-20.1	-24.3	-28.3	-32.1	-38.2	-39
	pred	-15.5	-20.1	-23.9	-27.2	-30	-32.7	-35.3
mn 2 eq acac ax acac	data	-10.1	-18.6	-25	-30.6	-35.5	-39.9	-43.9
	pred	-12.7	-16.9	-21.5	-26.4	-31.1	-34.8	-37.8
mn 2 eq en ax cn	data	3.26	-2.16	-7.31	-12.2	-16.8	-22.7	-28.6
	pred	8.53	1.06	-6.33	-13	-19.7	-25.8	-29.6
mn 2 eq bipy ax h2o	data	-14.6	-20.3	-25.5	-30.2	-34.5	-38.5	-42.1
	pred	-15.6	-21.3	-26.9	-31.7	-35.2	-37.4	-39.1
mn 2 eq h2o ax h2o	data	-35.2	-38.8	-42.1	-45.4	-48.2	-50.8	-53.3
	pred	-30.8	-34.3	-37.4	-40.4	-43.1	-45	-46.2
mn 2 eq ncs ax nh3	data	-12.4	-18	-23.1	-25.4	-29.9	-33.9	-37.8
	pred	-13.1	-20	-24	-26.7	-29.6	-32.4	-35.1
mn 2 eq bipy ax bipy	data	4.7	-2.95	-9.9	-16.2	-21.9	-27	-31.6
	pred	0.523	-3.75	-9.54	-15.7	-20.3	-24.3	-28.1
mn 2 eq ox ax ox	data	-33.9	-40.1	-45	-49.1	-51.7	-	-
	pred	-35.4	-41.7	-45.4	-47.3	-48.5		
mn 2 eq c2h3ns ax c2h3ns	data	43.7	34.5	25.6	17.2	9.17	1.6	-5.51
	pred	39.5	32.2	25.4	16.5	6.17	-0.402	-5.2
mn 2 eq ncs ax co	data	27.7	18.7	9.92	1.59	-6.26	-13.6	-20.5
	pred	21.9	17.4	9.61	1.72	-5.24	-12.3	-16.9
mn 2 eq ncs ax ncs	data	-25.9	-32	-37.4	-42.4	-46.8	-50.8	-54.2
	pred	-24	-30	-35.1	-38.9	-41.8	-44.5	-47.1
mn 2 eq phen ax phen	data	3.97	-3.6	-10.5	-16.7	-22.3	-27.3	-31.8
	pred	-1.38	-7.37	-14.1	-19.2	-23.5	-27	-29.9
mn 2 eq nh3 ax co	data	15.5	7.04	-1.01	-8.61	-15.7	-22.4	-28.5
	pred	12.8	6.28	0.325	-7.14	-14.5	-20	-23.8
mn 2 eq en ax h2o	data	-23.8	-27.4	-30.8	-34.2	-37.3	-40.3	-43.2
	pred	-20.4	-25.3	-30.6	-35	-37.8	-39.7	-41.4
mn 2 eq en ax co	data	24.8	15.9	7.43	-0.592	-8.16	-15.3	-21.9
	pred	21.2	15.3	6.96	-1.33	-8.26	-14.6	-20.8
mn 2 eq nh3 ax pisc	data	4.37	-2.39	-8.82	-14.9	-20.6	-25.8	-30.6
	pred	2.92	-2.32	-8.51	-15.5	-23.2	-28.4	-32.7
mn 3 eq en ax co	data	1.61	-3.86	-6.11	-9.5	-14.1	-15.5	-18.2
	pred	2.03	-3.08	-6.09	-8.05	-9.53	-10.8	-12
mn 3 eq phen ax cl	data	1.51	-1.15	-3.57	-5.85	-8.03	-10.6	-12.2
	pred	-3.49	-4.15	-5.02	-6.44	-8.3	-9.99	-11.3
mn 3 eq phen ax phen	data	10	7.49	5.17	2.92	0.823	-1.16	-3.08
	pred	7.47	5.32	3.61	1.77	-0.2	-2.13	-3.99
mn 3 eq cn ax nh3	data	5.43	3.11	0.654	-1.68	-3.95	-6.12	-8.21
	pred	7.77	3.6	0.791	-1.48	-3.27	-4.56	-5.58
mn 3 eq pisc ax pisc	data	41.1	35.9	30.8	26	21.7	17.7	14.1
	pred	37.4	34.9	30.6	25.7	23.2	21.5	19.4
mn 3 eq ncs ax cl	data	-1.42	-4.14	-6.69	-9.08	-11.4	-13.5	-15.6
	pred	-3.51	-4.94	-6.59	-8.58	-10.9	-13	-14.6
mn 3 eq h2o ax co	data	2.61	0.268	-2.03	-4.21	-6.34	-8.39	-10.4
	pred	5.39	2.42	-0.819	-3.66	-5.99	-7.96	-9.77
mn 3 eq ox ax ox	data	-3.68	-5.71	-7.44	-9.07	-10.6	-12.2	-13.7
	pred	-3.51	-4.11	-5.14	-7.28	-10.2	-13.4	-17.1
mn 3 eq co ax co	data	17.1	13.7	10.4	7.39	4.55	1.84	-0.719
	pred	17.3	14.8	12.1	9.32	7.06	5.26	3.76
mn 3 eq acac ax acac	data	2.92	0.269	-2.31	-4.3	-6.33	-8.23	-10.1
	pred	2.49	0.145	-1.63	-2.81	-3.89	-5.14	-6.8
mn 3 eq nh3 ax nh3	data	2.3	0.504	-1.46	-3.21	-4.95	-6.66	-8.38
	pred	2.89	1.01	-0.523	-2.06	-3.95	-6.09	-8.09
mn 3 eq nh3 ax co	data	0.0138	-3.5	-6.74	-9.76	-12.5	-15.2	-17.6
	pred	4.27	1.18	-1.99	-4.59	-6.65	-8.49	-10.1
mn 3 eq cn ax cn	data	22	18.5	15.2	12.1	9.18	9.18	6.52
	pred	21.8	19.3	16.6	13.9	11.3	9.12	7.06
mn 3 eq nh3 ax scn	data	-16.5	-18.1	-19.6	-21.3	-23.1	-24.9	-26.7
	pred	-14.4	-17	-19.6	-21.7	-23.6	-25.9	-28.9
mn 3 eq bipy ax bipy	data	8.54	6.05	3.71	1.55	-0.452	-2.38	-4.22
	pred	10	6.76	4.65	2.89	1.13	-0.754	-2.82
mn 3 eq h2o ax pisc	data	-8	-14.7	-21.2	-31.5	-36.5	-41	-
	pred	-6.24	-13.3	-20.7	-25.2	-27.4	-28.6	
mn 3 eq en ax en	data	3.7	1.92	0.149	-1.72	-3.42	-5.11	-6.75
	pred	3.89	1.54	-1.02	-2.83	-4.02	-4.94	-5.86

					a_{HF}			
name	type	0.00	0.05	0.1	0.15	0.20	0.25	0.30
mn 3 eq phen ax nh3	data	-1.37	-3.2	-5.05	-6.89	-8.72	-10.5	-12.3
	pred	1.99	-1.46	-3.93	-5.6	-6.81	-7.76	-8.61
mn 3 eq h2o ax h2o	data	-5.06	-6.61	-8.16	-9.71	-11.3	-12.8	-14.3
	pred	-4.55	-5.59	-6.52	-7.69	-9.32	-11.4	-13.7
mn 3 eq c2h3ns ax c2h3ns	data	28.9	25.3	21.9	18.7	15.7	12.8	10.1
	pred	26.7	22.2	19.4	16.8	14	10.8	7.61
fe 2 eq co ax co	data	66.7	57.1	47.8	38.8	30.3	22.2	14.5
	pred	60.4	53.8	45.4	37.5	29	21.2	15.4
fe 2 eq acac ax acac	data	8.06	2.18	-3.18	-8.1	-20	-23.5	-23.7
	pred	3.11	-0.278	-4.38	-9.21	-14.3	-19.4	-24.9
fe 2 eq tbuc ax co	data	14.6	10.3	5.78	1.88	-3.22	-8.37	-4.03
	pred	16.7	11	5.37	0.76	-3.93	-8.83	-13.9
fe 2 eq cn ax cn	data	36.6	27.5	18.8	10.5	2.92	-4.09	-10.5
	pred	36.7	26.4	16.7	7.32	0.181	-2.82	-4.33
fe 2 eq tbuc ax tbuc	data	-17.6	-22.2	-26	-29.5	-32.6	-35.6	-
	pred	-18.9	-24.4	-27.8	-29.5	-30.4	-30.9	
fe 2 eq h2o ax ncs	data	-9.37	-12.1	-14.7	-17.3	-19.9	-22.5	-25
	pred	-13.8	-15.4	-16.6	-18.4	-21.4	-24.7	-27.5
fe 2 eq en ax en	data	10.5	6.41	2.41	-1.39	-5.03	-8.5	-11.8
	pred	9.48	3.75	-0.159	-3.37	-7.38	-10.3	-13.3
fe 2 eq cl ax pisc	data	15.9	10.6	5.14	-0.496	-6.23	-13.4	-18.8
	pred	16.7	12.7	8.18	2.66	-4.36	-10.8	-15
fe 2 eq ncs ax h2o	data	-6.22	-9.95	-13.6	-17.6	-20.9	-23.9	-26.8
	pred	-7.85	-11.6	-15.7	-20.1	-23.3	-26.2	-29
fe 2 eq pisc ax cl	data	47.2	40.6	33.8	27	20.3	13.8	7.51
	pred	44.6	36.1	30.1	24.6	17.8	12	7.84
fe 2 eq phen ax phen	data	27.5	21	15.1	9.58	1.73	-2.67	-7.04
	pred	18.4	13.9	9.73	5.14	0.668	-3.22	-7.89
fe 2 eq scn ax h2o	data	-5.53	-8.55	-11.6	-14.8	-17.9	-17.3	-20.5
	pred	-6.56	-8.09	-9.57	-11.2	-13.3	-16	-18.6
fe 2 eq nh3 ax nh3	data	3.93	0.0674	-3.64	-7.18	-10.6	-13.8	-16.5
	pred	2.23	-2.84	-6.52	-10	-12.8	-14.5	-15.9
fe 2 eq c2h3ns ax c2h3ns	data	75.1	66.6	58.2	50.2	42.2	34.4	27.2
	pred	69.8	64.8	57.8	49.3	40.1	32.8	26.3
fe 2 eq ox ax pisc	data	28.8	22.9	17	11.3	5.53	-0.106	-8.43
	pred	22.4	19.4	15	8.85	1.78	-4.02	-9.1
fe 2 eq pisc ax cn	data	81.2	73.2	65.8	57.4	57.4	41	33.2
	pred	69.1	67	64.7	60.3	51	38.9	30.4
fe 2 eq acac ax cn	data	16.2	9.91	4.21	-1.67	-4.58	-11.6	-14.9
	pred	12.8	7.53	1.76	-3.31	-8.46	-13.4	-16.2
fe 2 eq ox ax ox	data	-13.7	-18.6	-22.7	-26.4	-29.8	-33	-35.9
	pred	-18.5	-21.9	-25.2	-27.4	-28.6	-29.2	-29.7
fe 2 eq h2o ax h2o	data	-14	-16.9	-20.1	-23.1	-26.1	-28.9	-31.5
	pred	-11.4	-16.5	-21.1	-23.8	-25.7	-27.4	-29.1
fe 2 eq ncs ax pisc	data	20.3	15	9.12	3.37	-1.55	-	-4.39
	pred	14.4	10.6	6.23	1.36	-2.84		-9.89
fe 2 eq en ax co	data	41.2	33.5	25.9	18.6	11.6	5.18	-0.805
	pred	35.4	29.8	23	16.7	11.8	6.1	-0.0024
fe 2 eq pisc ax ncs	data	47.6	33.4	27.6	21.5	17.4	11.3	5.4
	pred	33.2	29.3	23.3	17.5	12.1	6.57	2.24
fe 2 eq ncs ax co	data	28.3	23.2	17.6	11.8	5.85	0.102	-5.36
	pred	29.9	22.1	14.8	10.1	6.06	1.72	-2.7
fe 2 eq ncs ax ncs	data	-1.72	-6.89	-11.7	-16.2	-20.3	-24.2	-27.7
	pred	-5.55	-9.9	-14.1	-18	-21.9	-25.7	-28.3
fe 3 eq tbuc ax tbuc	data	-7.95	-11.1	-14.1	-17.2	-20.2	-23.2	-26.3
	pred	-7.56	-10.7	-13.3	-15.9	-18.4	-20.7	-22.9
fe 3 eq cn ax cn	data	57.9	51.8	46	40.3	34.8	29.4	24.2
	pred	54.6	49.7	45.7	41.7	36.3	28.9	19.5
fe 3 eq acac ax acac	data	8.94	5.47	2.02	-1.45	-4.97	-8.55	-12.2
	pred	6.45	3.35	0.96	-0.856	-2.91	-5.33	-7.55
fe 3 eq porphyrin ax h2o	data	18.7	15.3	12.1	8.76	-1.43	3.11	0.871
	pred	19.3	14.8	9.89	4.73	0.907	-1.44	-3.15
fe 3 eq h2o ax cn	data	14.7	11.6	8.5	5.36	2.1	2.78	-0.841
	pred	13.5	9.08	6.51	4.84	3.29	1.46	-0.922
fe 3 eq porphyrin ax cn	data	44.8	39.8	35	31.7	27	22.4	17
	pred	38.8	36.8	34	30.1	25.5	20.6	16.2
fe 3 eq co ax co	data	43.4	37.2	31.2	25.5	20	14.7	9.5
	pred	45.5	36.8	30.4	24.7	19.3	15.1	12.2
fe 3 eq ox ax ox	data	2.6	-0.574	-3.78	-7.03	-10.3	-13.7	-17.1
	pred	2.68	-0.701	-4.73	-8.16	-11.4	-14.6	-17.8
fe 3 eq pisc ax h2o	data	28.9	24.6	17.1	15	9.63	2.92	-1.87
	pred	30.3	24.9	19.3	14.2	8.24	3.5	0.317
fe 3 eq acac ax cl	data	1.11	-7.09	-10.2	-16	-16.2	-15	-18.2
	pred	-6.49	-7.3	-8.33	-9.96	-12	-14.2	-16.3
fe 3 eq ox ax ncs	data	8.73	4.93	1.26	-2.36	-5.81	-9.44	-13.1
	pred	3.56	2.05	0.0278	-2.94	-6.08	-9.38	-13.4
fe 3 eq en ax en	data	22.7	19.6	16.3	10.2	9.6	6.1	2.56
	pred	18.8	15.4	12.2	9.55	6.96	4.6	2.45
fe 3 eq pisc ax pisc	data	65.5	61.4	54.9	49.5	40.8	34.4	26.1
	pred	59.1	56.7	52.9	46.3	38.7	31.9	27.3
fe 3 eq bipy ax co	data	29.7	23.9	16.7	10.1	3.55	-2.55	-16.9
	pred	29.9	24.7	19	11.9	4.95	-2.21	-8.63
fe 3 eq pisc ax ncs	data	41.5	42.5	32.3	26.5	27.2	20.4	13.9
	pred	38.4	32.2	27.8	24.8	22.1	18.2	12.9
fe 3 eq ncs ax cl	data	1.61	-1.64	-10.5	-13.5	-16.5	-19.5	-22.7
	pred	-0.111	-3.99	-8.3	-12.5	-15.8	-18.3	-20.4
fe 3 eq phen ax phen	data	28.6	25.2	21.7	14.7	11	7.17	3.21
	pred	24.8	22.9	19.7	15.5	11.3	7.49	4.24

					a_{HF}			
name	type	0.00	0.05	0.1	0.15	0.20	0.25	0.30
fe 3 eq h2o ax h2o	data	-15.6	-18	-21	-23.4	-26	-28.7	-31.6
	pred	-9.04	-13.4	-17	-19.1	-20.6	-22.3	-24.4
fe 3 eq cl ax cl	data	-13.9	-16.6	-26.3	-22.4	-25.5	-28.7	-38.6
	pred	-9.3	-13.4	-17.3	-21.9	-26.6	-30.2	-32.4
fe 3 eq pisc ax cl	data	39.1	34.1	35.7	30.7	25.8	21.1	16.5
	pred	38	34.8	31.8	29.2	26.4	22.3	17.4
fe 3 eq c2h3ns ax c2h3ns	data	60.6	54.7	48.9	43.2	37.7	32.3	27
	pred	62.1	55.4	47.4	41.9	36.9	31.7	27.6
fe 3 eq nh3 ax scn	data	20.7	17.2	13.9	10.7	7.65	4.53	1.13
	pred	13.2	11.6	9.86	7.93	5.56	2.32	-2.12
fe 3 eq acac ax ncs	data	-6.13	-9.36	-12.4	-15.4	-18.3	-21.1	-23.9
	pred	-5.44	-9.91	-12.7	-14.3	-15.4	-16.1	-16.7
fe 3 eq co ax scn	data	-	-	-8.39	-12.8	-17.3	-21.9	-26.4
	pred			-10.9	-15.3	-18.3	-21	-23.7
fe 3 eq bipy ax bipy	data	29.7	29.4	25.3	21.1	16.9	12.7	8.53
	pred	25.3	23.9	21.4	17.7	13.7	9.78	5.89
fe 3 eq ncs ax cn	data	19.9	16	12.2	8.44	4.74	1.02	-2.73
	pred	20.2	15.9	12.1	8.41	4.56	1.19	-1.98
fe 3 eq ncs ax ncs	data	0.0285	-3.24	-6.41	-9.52	-12.6	-15.7	-18.8
	pred	-2.02	-6.27	-9.38	-11.6	-13.2	-14.5	-15.9
fe 3 eq nh3 ax nh3	data	21	17.2	13.4	9.59	5.8	2.03	-1.73
	pred	19.2	15.6	12.3	8.96	5.6	1.78	-3.37
fe 3 eq cl ax pisc	data	20.2	15.1	10.3	5.6	4.97	0.215	-4.42
	pred	19.1	15.6	10.9	5.82	2.28	0.0921	-1.74
co 2 eq ox ax ox	data	-9.29	-12.1	-14.4	-16.5	-18.5	-20.3	-22
	pred	-13.1	-14.5	-16.3	-18.3	-20.2	-21.9	-23.4
co 2 eq en ax en	data	3.16	0.284	-2.48	-5.19	-7.81	-10.4	-12.7
	pred	1.72	-1.62	-4.23	-6.52	-9.06	-12.3	-16.1
co 2 eq tbuc ax tbuc	data	-11.5	-13.7	-15.6	-17.5	-19.2	-20.8	-22.4
	pred	-13.8	-15.4	-17.1	-18.8	-20.4	-22	-23.5
co 2 eq cl ax pisc	data	11.5	8.82	5.5	1.8	-2.05	-5.91	-9.65
	pred	11	8.74	6.3	3.46	0.241	-3.54	-7.66
co 2 eq cn ax h2o	data	-30.3	-32.2	-34.6	-37.3	-40.1	-43	-46
	pred	-24	-28.8	-32	-33.7	-34.5	-34.7	-34.8
co 2 eq en ax pisc	data	16.4	12.5	8.23	4.11	0.192	-3.49	-6.93
	pred	8.45	7.32	5.99	4.04	0.65	-3.56	-6.03
co 2 eq phen ax h2o	data	4.62	1.07	-2.3	-5.49	-6.62	-9.37	-14.7
	pred	3.97	1.33	-1.55	-4.37	-6.51	-7.96	-9.16
co 2 eq phen ax phen	data	8.28	4.41	0.79	-2.61	-5.77	-8.74	-11.5
	pred	5.96	1.86	-1.39	-4.16	-6.6	-9.13	-12.5
co 2 eq h2o ax ncs	data	-4.76	-6.41	-8.51	-10.2	-11.9	-13.5	-15
	pred	-7.4	-8.68	-9.82	-10.9	-12	-13.6	-16.5
co 2 eq c2h3ns ax c2h3ns	data	33.5	25.4	20.3	15.4	10.6	6.15	1.91
	pred	32.7	25.4	19	13.6	9.01	4.8	1.71
co 2 eq nh3 ax nh3	data	0.576	-2.17	-4.44	-7.45	-9.97	-12.3	-14.4
	pred	0.629	-1.49	-4.75	-8.69	-11.7	-13.4	-14.4
co 2 eq acac ax cn	data	12.5	8.82	5.29	1.95	-0.987	-3.12	-6.87
	pred	11.1	7.41	3.23	-0.215	-2.97	-5.27	-7.41
co 2 eq nh3 ax h2o	data	7.28	4.48	1.66	-1.14	-3.86	-6.53	-9.11
	pred	1.72	-0.547	-2.54	-4.16	-5.73	-7.47	-9.44
co 2 eq acac ax acac	data	-3.04	-6.6	-9.73	-12.5	-14.9	-13.2	-15.4
	pred	-7.52	-10	-11.9	-13.3	-14.2	-15.1	-16
co 2 eq ncs ax ncs	data	-8.26	-10.8	-13.2	-15.4	-17.4	-19.2	-20.9
	pred	-11.3	-14.7	-17	-18.2	-19.2	-20.5	-22.6
co 2 eq bipy ax bipy	data	8.05	4.07	0.383	-3.04	-6.23	-9.03	-11.7
	pred	7.28	2.9	-0.524	-3.46	-6.07	-8.66	-11.9
co 2 eq co ax co	data	28.1	22.3	16.6	11.3	6.23	1.48	-5.44
	pred	25.3	19.6	14.4	9.27	4.86	1.67	-1.13
co 2 eq en ax h2o	data	12.8	9.86	6.97	4.13	1.33	-1.41	-4.11
	pred	7.53	4.81	1.61	-1.61	-4.29	-6.41	-8.35
co 2 eq h2o ax h2o	data	-11.8	-14.2	-16.2	-18.1	-19.7	-21.2	-22.6
	pred	-10.9	-12.9	-15.3	-18.2	-21.4	-23.8	-25.3
co 3 eq co ax nh3	data	-	57.3	52.4	47.5	42.4	37.5	32.8
	pred		52.4	49.3	45.6	40.9	36.2	32.4
co 3 eq acac ax nh3	data	29.4	30.2	27.7	25.2	22.7	20.2	17.8
	pred	29.8	28	25.5	22.9	20.7	18.6	15.8
co 3 eq tbuc ax pisc	data	11.8	9.19	6.23	-0.016	-3.36	-6.88	-11.7
	pred	10.2	8.16	5.98	2.95	-2.19	-9.67	-16.3
co 3 eq nh3 ax nh3	data	48.2	45	42.3	39.2	36.1	33	30
	pred	46.9	42.9	39.9	37.1	33.9	30.6	26.6
co 3 eq ox ax ox	data	-4.67	-7.87	-11.2	-14.6	-15.7	-21.6	-26
	pred	0.565	-1.88	-5.04	-8.91	-12.9	-16.7	-20.1
co 3 eq en ax en	data	49.6	47.7	45.4	42.8	40.1	37.3	34.4
	pred	46.7	43.9	40	35.4	31.2	28	24.9
co 3 eq acac ax acac	data	31.9	29.5	27.2	24.8	22.5	20.2	17.5
	pred	29.3	27.4	25.4	23.1	20.4	16.7	10.5
co 3 eq phen ax phen	data	43.2	40.5	36.5	32.6	28.7	24.7	20.9
	pred	48	45.7	42.7	39	35.5	32.6	30.1
co 3 eq cn ax cn	data	88.5	83.6	78.7	73.8	69.1	64.5	60
	pred	74.6	74	72.7	70.2	65.9	59.8	51.1
co 3 eq c2h3ns ax c2h3ns	data	90.7	86.9	82.7	78.2	73.7	69.1	64.6
	pred	74.8	74.6	74.2	72.8	69.4	63.6	56.7
co 3 eq co ax co	data	77	71.6	66.3	61.2	56.3	51.6	47
	pred	71.8	68.8	64	59	54.5	49.9	45.1
co 3 eq tbuc ax tbuc	data	2.61	1.38	0.259	-0.943	-2.55	-4.74	-7.18
	pred	5.13	3.38	1.48	-1.26	-5.64	-10.7	-15.6
co 3 eq h2o ax h2o	data	6.22	5.28	4.26	3.09	1.79	0.265	-1.44
	pred	11.5	8.36	5.78	3.39	1.51	0.0404	-1.79
co 3 eq ncs ax ncs	data	8.87	8.94	8.84	8.02	6.47	4.09	2.08

		a_{HF}						
name	type	0.00	0.05	0.1	0.15	0.20	0.25	0.30
	pred	15.6	12.6	9.55	5.42	1.15	-1.67	-4.04
co 3 eq pisc ax pisc	data	47.2	46.9	43.3	39.1	34.6	30.1	25.6
	pred	46.2	44.3	42.2	39.4	35.9	32.1	28.2
co 3 eq bipy ax bipy	data	54.4	51.8	48.7	44.7	40.6	36.6	32.7
	pred	48.2	45.8	42.8	39.2	35.5	32.4	29.8
co 3 eq acac ax ncs	data	6.48	4.32	2.33	-0.259	-3.87	-7.95	-12.2
	pred	7.31	4.81	2.25	-0.448	-3.55	-6.93	-10.2
co 3 eq ox ax ncs	data	20.5	19.2	19.4	14.8	10.2	5.84	1.61
	pred	21	18.3	15	11.5	8.37	5.4	2.52
co 3 eq acac ax co	data	23.7	19.6	15.8	11.5	7.16	2.7	-2.3
	pred	18.4	16.5	14.1	11.6	9.14	6.06	2.15
ni 2 eq tbuc ax tbuc	data	-19.1	-22.3	-25.4	-28.5	-31.5	-34.4	-37.3
	pred	-18.2	-21.8	-26.8	-30.8	-33.2	-34.9	-36.3
ni 2 eq bipy ax nh3	data	-13.2	-16.4	-19.2	-22	-24.9	-27.8	-30.7
	pred	-15.4	-18.3	-21	-23.7	-26.7	-29.6	-32
ni 2 eq en ax en	data	-18.8	-21.6	-24.3	-27.2	-30	-32.8	-35.6
	pred	-24.7	-25.2	-25.7	-26.6	-28.3	-30.6	-33
ni 2 eq co ax co	data	-21	-24.4	-27.8	-31.3	-34.6	-37.9	-41.1
	pred	-19.4	-22.8	-25.3	-28	-31	-34.3	-37.4
ni 2 eq nh3 ax nh3	data	0.552	-3.22	-6.79	-10.7	-14.5	-18.2	-21
	pred	-4.75	-6.97	-10	-13.4	-16.1	-18.5	-21.3
ni 2 eq acac ax cn	data	-10.3	-13.4	-16.5	-19.5	-22.5	-25.4	-28.2
	pred	-9.03	-12.4	-16.8	-21.2	-23.9	-25.2	-26.4
ni 2 eq pisc ax pisc	data	2.09	-1.92	-6.39	-10.6	-14.4	-18.2	-21.9
	pred	-2.05	-4.82	-7.77	-10.8	-13.9	-16.8	-19.8
ni 2 eq en ax h2o	data	1.41	-1.64	-4.68	-7.78	-10.8	-13.9	-17
	pred	3.16	-1.42	-6.98	-11.8	-14.7	-16.6	-18.7
ni 2 eq bipy ax bipy	data	-17.1	-20.3	-23.5	-26.6	-29.8	-32.8	-35.8
	pred	-23.4	-24.3	-25	-25.8	-27.1	-28.9	-31.2
ni 2 eq ncs ax ncs	data	-32	-34.9	-37.8	-40.5	-43.1	-45.6	-47.9
	pred	-30.9	-33.4	-36	-39.6	-42.4	-43.9	-44.6
ni 2 eq cn ax cn	data	-	-	-20.4	-23.8	-27.1	-30.4	-33.5
	pred			-20.5	-25.5	-28.8	-31.3	-33.8
ni 2 eq acac ax co	data	-13.3	-15.4	-17.8	-20.5	-23.3	-26.1	-28.8
	pred	-12.6	-14.9	-17.3	-20	-22.5	-24.7	-26.7
ni 2 eq c2h3ns ax c2h3ns	data	-19.5	-22.5	-25.6	-28.8	-32	-35.1	-38.1
	pred	-18.1	-22.1	-25.4	-28.1	-30	-32	-34.6
ni 2 eq acac ax nh3	data	-23.5	-25.9	-28.4	-31	-33.5	-36.1	-38.5
	pred	-22.4	-26.5	-29.5	-31.9	-33.8	-35.4	-36.7
ni 2 eq acac ax acac	data	-23.3	-26.8	-25.2	-28.9	-32.5	-35.9	-39.2
	pred	-23.4	-25	-26.9	-29.3	-32.3	-35	-37.2
ni 2 eq ox ax ox	data	-17.5	-21.2	-24.8	-28.6	-31.9	-35.1	-38.2
	pred	-17.9	-20.6	-24	-28.2	-31.8	-33.9	-35.3

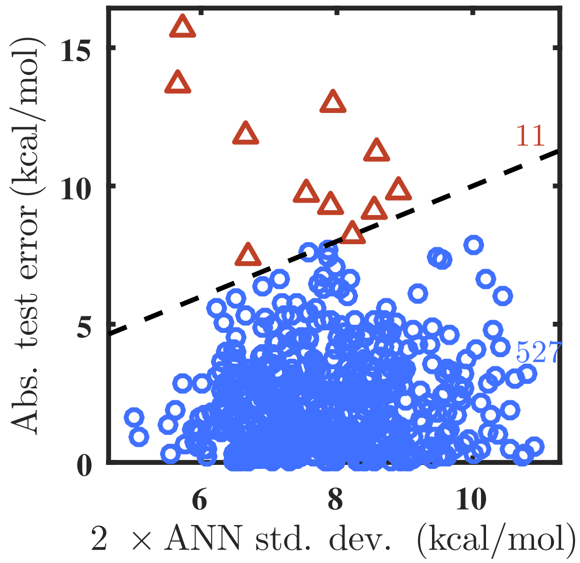

Figure S7: Parity plot for 2 standard deviation from the mean prediction and absolute prediction error for test case $\Delta E_{H\text{-}L}$ prediction using ANN. All units are kcal/mol. The black line is y=x.

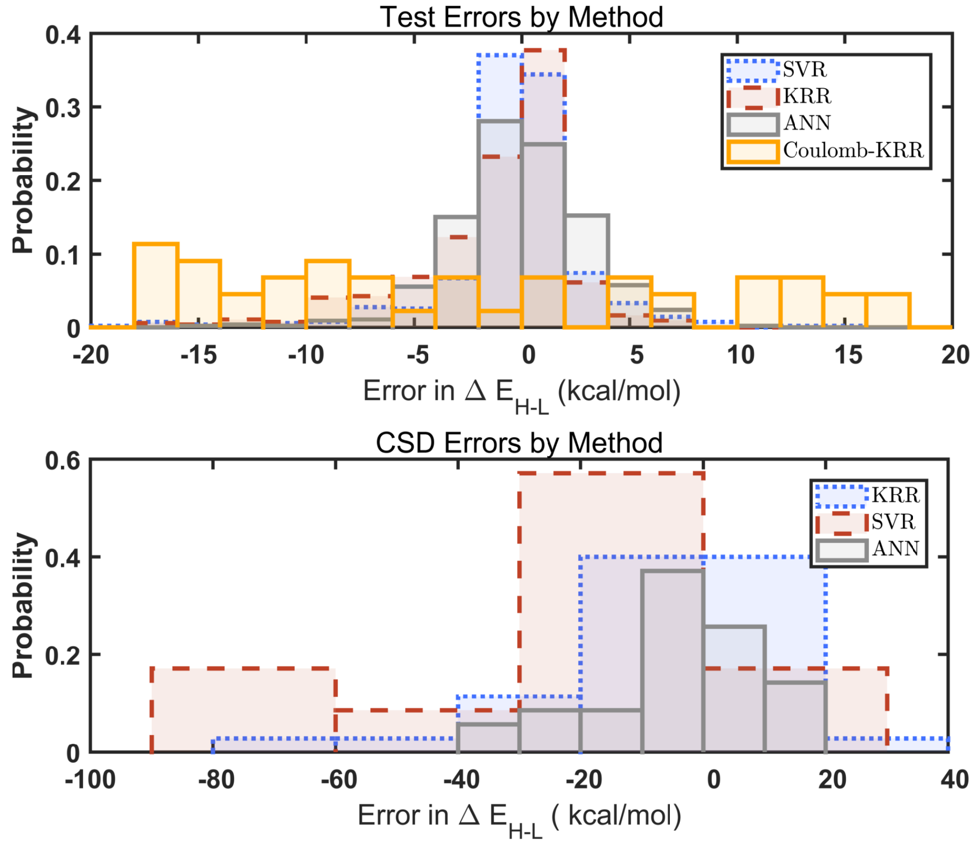

Figure S8: Normalized error histogram for HF = 0.2 (B3LYP) test data (top) and CSD structures (bottom), comparing ANN, KRR and SVR models using descriptor set **g**, as well as a KRR model using the Coulomb matrix descriptor (trained on B3LYP data only.)

Table S24: RMS prediction errors for $\Delta E_{H\text{-}L}$ on test data using a deep ANN in kcal/mol for test data divided by metal and oxidation state. The number of test cases is indicated in parentheses

Species	RMS Test error	Min. Abs. Test Error	Max. Abs. Test Error
Cr(II)	3.3 (86)	0.03	11.2
Cr(III)	2.5 (50)	0.06	6.3
Mn(II)	2.8 (57)	0.01	7.5
Mn(III)	2.1 (60)	0.04	5.6
Fe(II)	3.7 (60)	0.07	13.0
Fe(III)	3.0 (71)	0.09	7.9
Co(II)	2.8 (58)	0.07	11.8
Co(III)	4.5 (57)	0.02	15.7
Ni(II)	2.5 (39)	0.09	5.8

Table S25: Average HF exchange sensitivity values, in kcal/mol.HFX, for homoleptic compounds with C, N and O ligands grouped by metal, oxidation state and ligand connecting atom

Metal	Oxidation state	Ligand connection		
		C	N	O
Cr	II	-82	-55	-59
	III	-20	-20	-18
Mn	II	-167	-92	-87
	III	-58	-44	-32
Fe	II	-164	-76	-59
	III	-118	-74	-63
Co	II	-106	-48	-45
	III	-98	-53	-46
Ni	II	-65	-57	-52

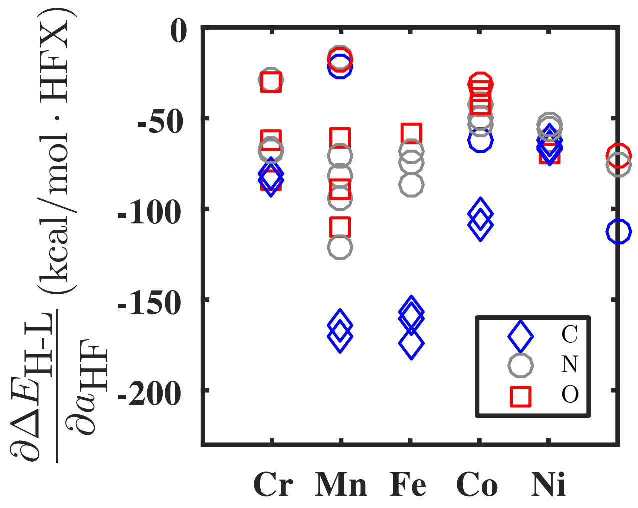

Figure S9: Scatter plot of HFX sensitivity in kcal/mol · HFX for homoleptic M(II) complexes, colored by connection atom, for homoleptic (II) complexes with C (gray), N (blue) and O (square) ligands.

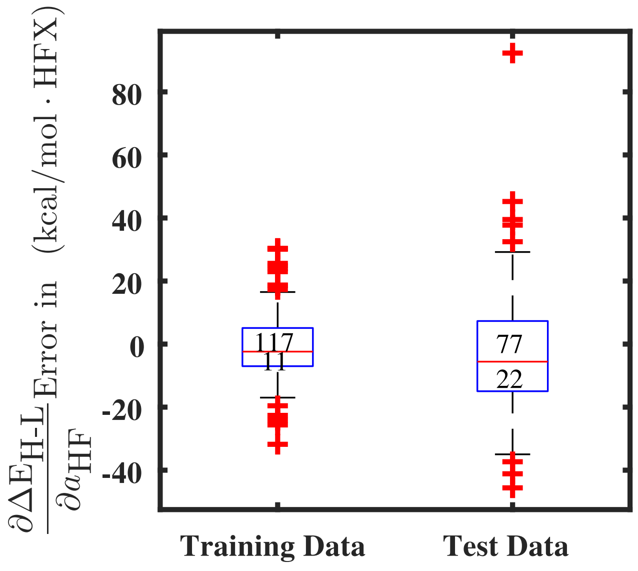

Figure S10: Error boxplot for regression of $\frac{\partial \Delta \text{E}_{\text{H-L}}}{\partial a_{\text{HF}}}$ using an ANN, showing training and test data comparison. The top number indicates the number of trials, while the bottom indicates the RMSE.

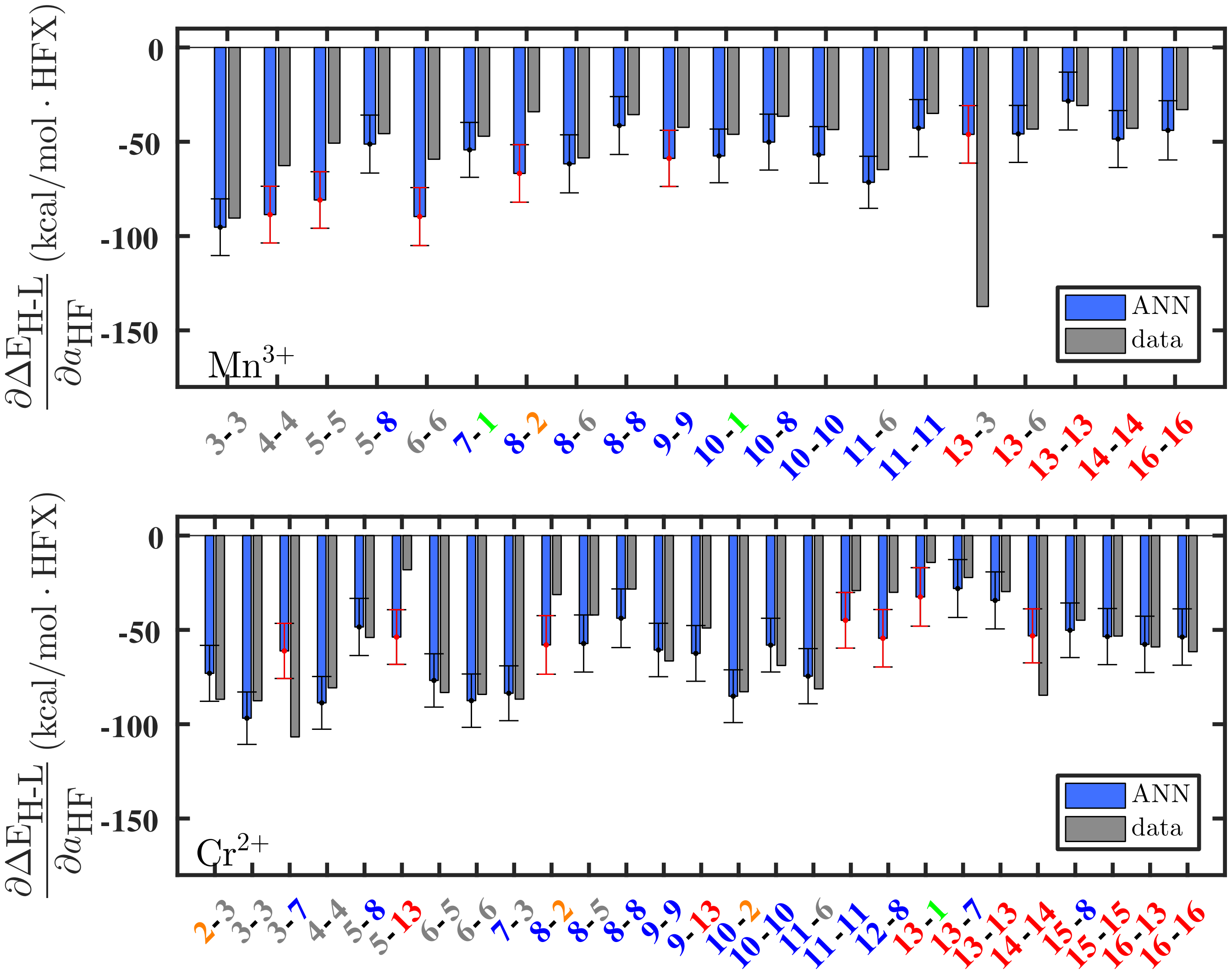

Figure S11: Model predictions of $\frac{\partial \Delta E_{\text{H-L}}}{\partial a_{\text{HF}}}$ and data for Co using an ANN. The ligands are described by two numbers indicating the equatorial first and then the axial, color coded by ligand identity (green for halogen, gray for carbon, blue for nitrogen, and red for oxygen). The error bars represent an estimated 1 standard deviation from the mean prediction.

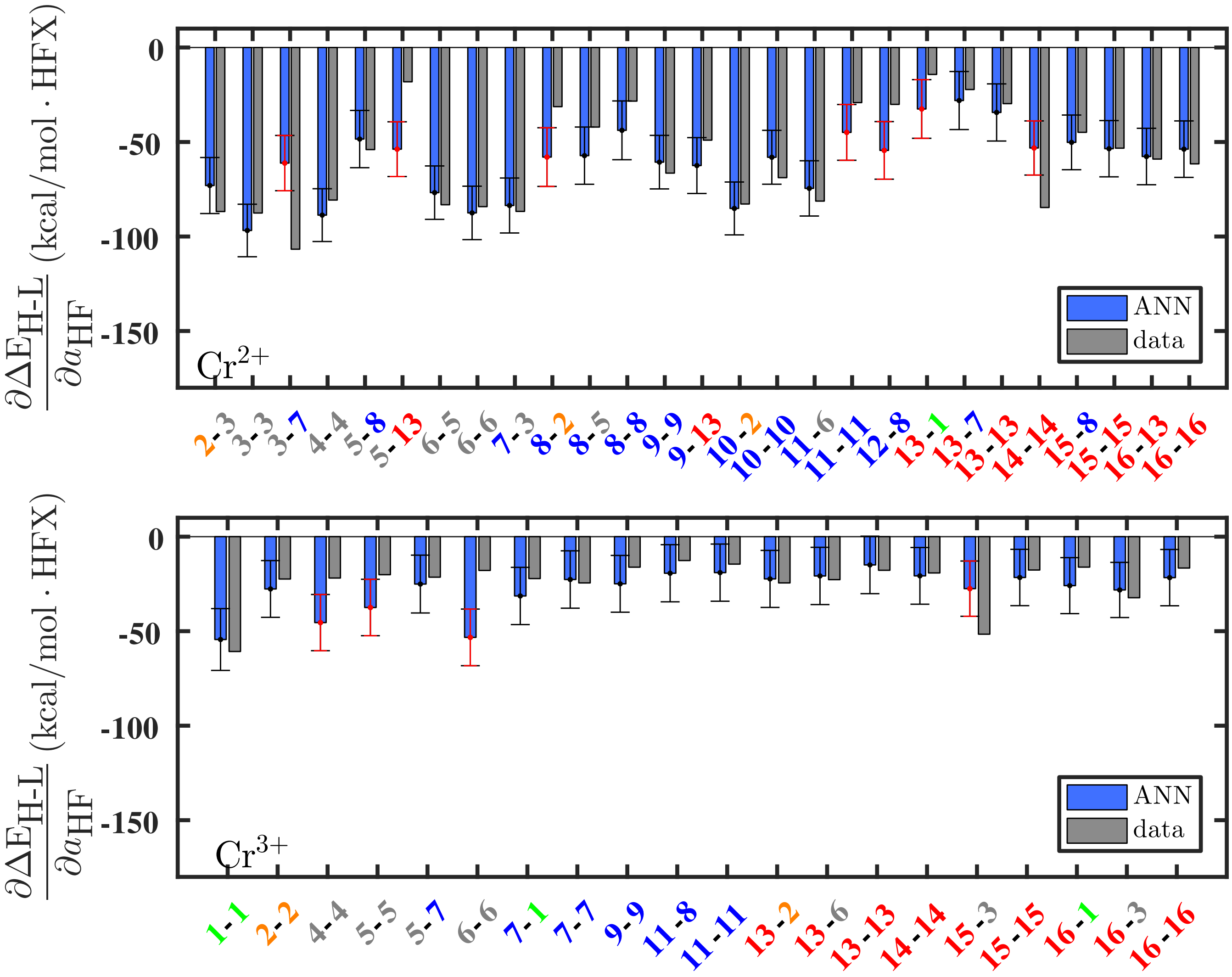

Figure S12: Model predictions of $\frac{\partial \Delta E_{\text{H-L}}}{\partial a_{\text{HF}}}$ and data for Cr using an ANN. The ligands are described by two numbers indicating the equatorial first and then the axial, color coded by ligand identity (green for halogen, gray for carbon, blue for nitrogen, and red for oxygen). The error bars represent an estimated 1 standard deviation from the mean prediction.

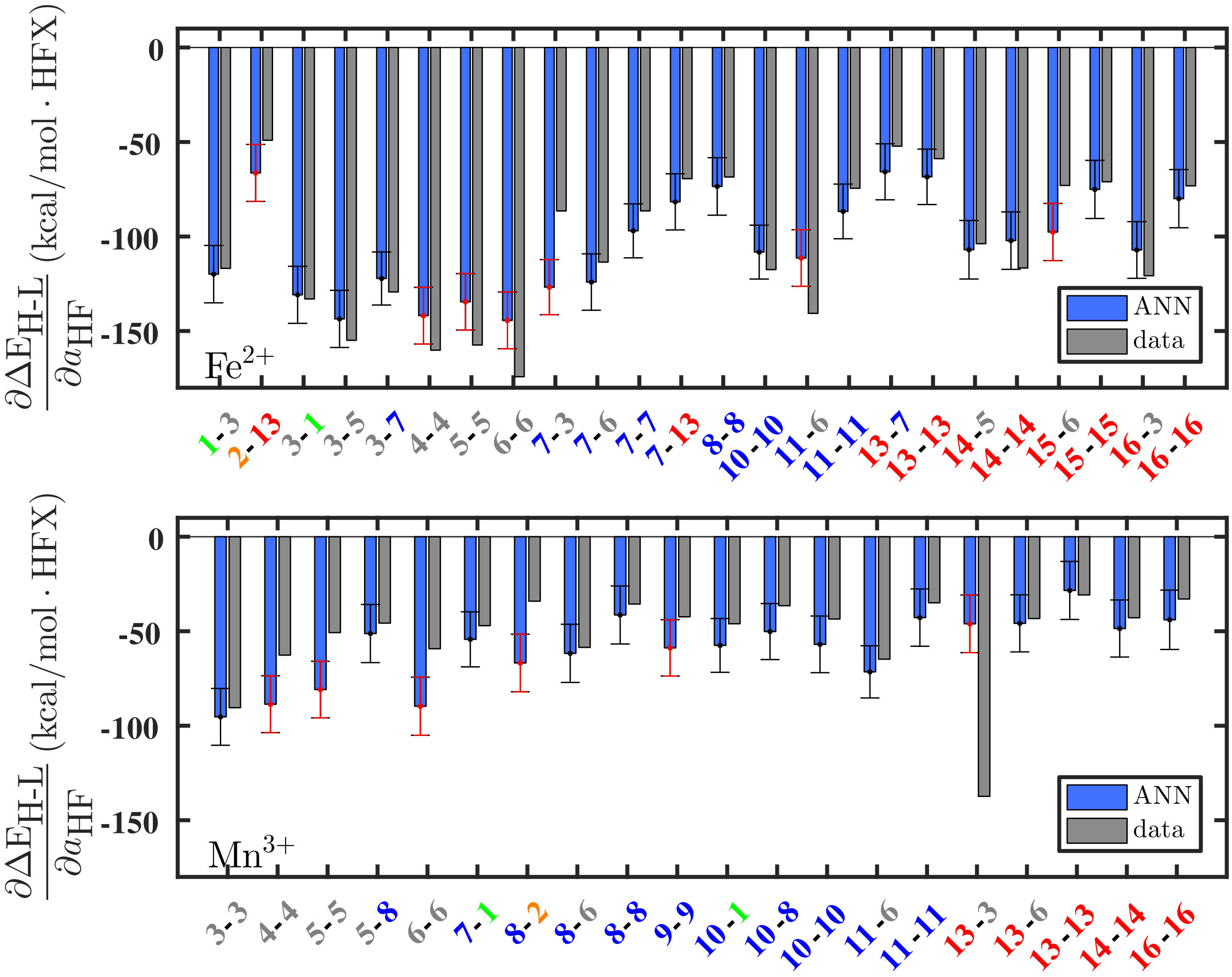

Figure S13: Model predictions of $\frac{\partial \Delta E_{\text{H-L}}}{\partial a_{\text{HF}}}$ and data for low-spin Fe(II) (top) and Ni(II) (bottom) using an ANN. The ligands are described by two numbers indicating the equatorial first and then the axial, color coded by ligand identity (green for halogen, gray for carbon, blue for nitrogen, and red for oxygen). The error bars represent an estimated 1 standard deviation from the mean prediction.

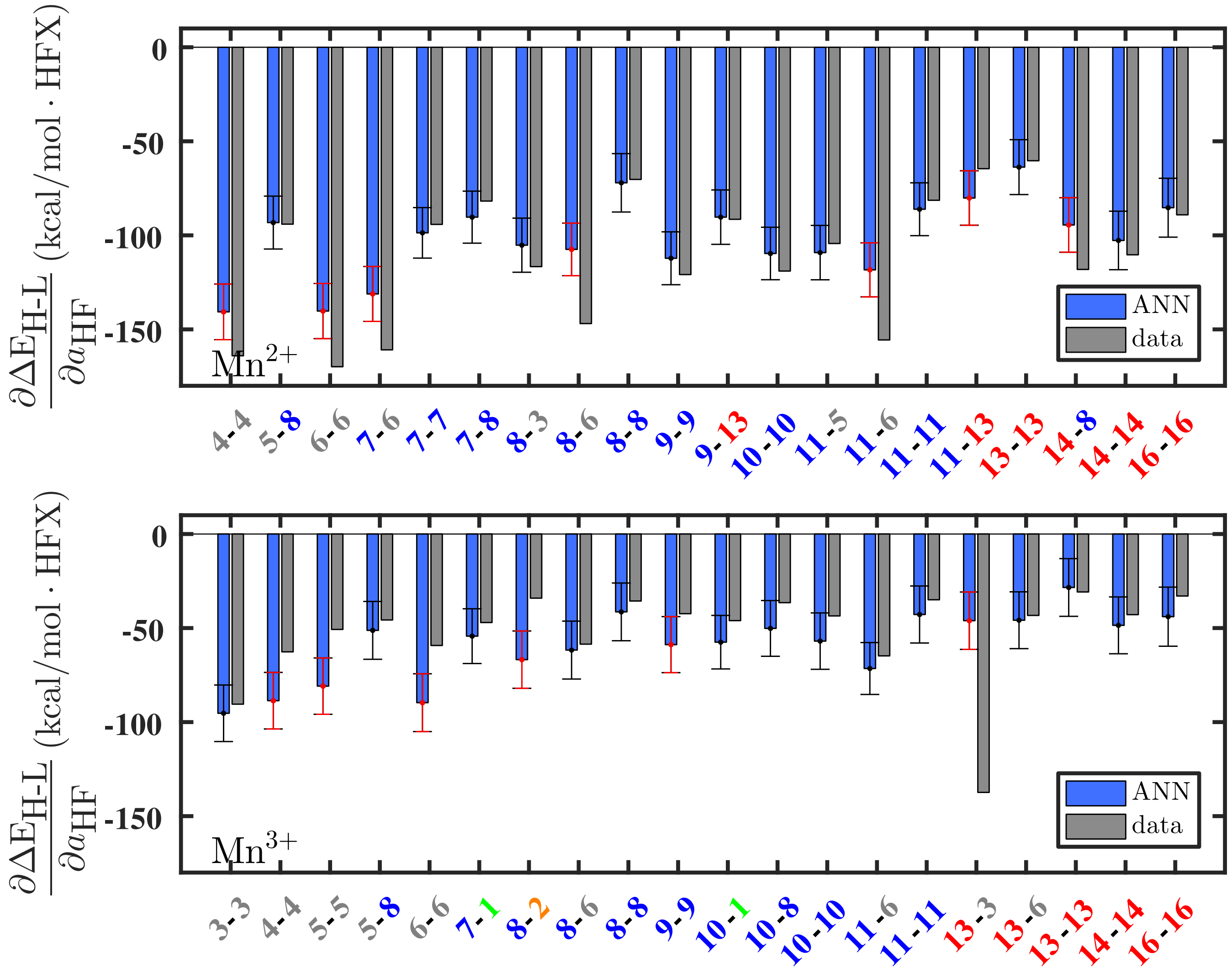

Figure S14: Model predictions of $\frac{\partial \Delta E_{\text{H-L}}}{\partial a_{\text{HF}}}$ and data for Mn using an ANN. The ligands are described by two numbers indicating the equatorial first and then the axial, color coded by ligand identity (green for halogen, gray for carbon, blue for nitrogen, and red for oxygen). The error bars represent an estimated 1 standard deviation from the mean prediction.

Table S26: HFX sensitivity predictions and data compared in kcal/mol·HFX, compared for the full set of complexes. Each complex is named [metal oxidation axial ligand eq equatorial ligand]. For each complex, the first row has the data obtained from DFT and the second row has the ANN predictions

name	type	HFX sensitivity
cr 2 eq h2o ax ncs	data	-22.2
	pred	-28.1
cr 2 eq porphyrin ax nh3	data	-30.1
	pred	-54.4
cr 2 eq scn ax pisc	data	-86.8
	pred	-73
cr 2 eq ncs ax pisc	data	-86.7
	pred	-83.6
cr 2 eq cn ax nh3	data	-54
	pred	-48.4
cr 2 eq tbuc ax tbuc	data	-53.3
	pred	-53.5
cr 2 eq phen ax scn	data	-82.8
	pred	-85.2
cr 2 eq en ax co	data	-81.3
	pred	-74.5
cr 2 eq bipy ax bipy	data	-66.5
	pred	-60.7
cr 2 eq en ax en	data	-29.2
	pred	-44.9
cr 2 eq co ax cn	data	-83.2
	pred	-76.8
cr 2 eq pisc ax pisc	data	-87.6
	pred	-96.8
cr 2 eq ox ax h2o	data	-59
	pred	-57.7
cr 2 eq h2o ax cl	data	-14.3
	pred	-32.5
cr 2 eq nh3 ax scn	data	-31.3
	pred	-58
cr 2 eq ox ax ox	data	-61.6
	pred	-53.8
cr 2 eq h2o ax h2o	data	-29.7
	pred	-34.4
cr 2 eq cn ax h2o	data	-18.1
	pred	-53.8
cr 2 eq pisc ax ncs	data	-107
	pred	-61.2
cr 2 eq acac ax acac	data	-84.7
	pred	-53.2
cr 2 eq bipy ax h2o	data	-49
	pred	-62.5
cr 2 eq nh3 ax nh3	data	-28.4
	pred	-43.8
cr 2 eq co ax co	data	-84.2
	pred	-87.5
cr 2 eq c2h3ns ax c2h3ns	data	-80.7
	pred	-88.7
cr 2 eq phen ax phen	data	-68.9
	pred	-58.1
cr 2 eq tbuc ax nh3	data	-44.9
	pred	-50.2
cr 2 eq nh3 ax cn	data	-42.1
	pred	-57.2
cr 3 eq c2h3ns ax c2h3ns	data	-21.9
	pred	-45.5
cr 3 eq acac ax acac	data	-19.2
	pred	-20.7
cr 3 eq h2o ax scn	data	-24.5
	pred	-22.3
cr 3 eq en ax nh3	data	-12.6
	pred	-19.3
cr 3 eq scn ax scn	data	-22.4
	pred	-27.7
cr 3 eq en ax en	data	-14.5
	pred	-19
cr 3 eq cn ax cn	data	-20.1
	pred	-37.5
cr 3 eq cn ax ncs	data	-21.4
	pred	-25.1
cr 3 eq tbuc ax tbuc	data	-17.6
	pred	-21.6
cr 3 eq ncs ax ncs	data	-24.5
	pred	-22.7
cr 3 eq ox ax ox	data	-16.6
	pred	-21.7
cr 3 eq cl ax cl	data	-60.7
	pred	-54.4
cr 3 eq co ax co	data	-17.9

	pred	-53.3
cr 3 eq h2o ax co	data	-22.7
	pred	-20.8
cr 3 eq bipy ax bipy	data	-16.1
	pred	-24.9
cr 3 eq ncs ax cl	data	-22.2
	pred	-31.4
cr 3 eq tbuc ax pisc	data	-51.6
	pred	-27.5
cr 3 eq ox ax cl	data	-16.1
	pred	-25.9
cr 3 eq h2o ax h2o	data	-17.8
	pred	-14.9
cr 3 eq ox ax pisc	data	-32.3
	pred	-28.2
mn 2 eq acac ax nh3	data	-118
	pred	-94.5
mn 2 eq nh3 ax nh3	data	-70.3
	pred	-72.1
mn 2 eq co ax co	data	-170
	pred	-140
mn 2 eq cn ax nh3	data	-94.1
	pred	-93.2
mn 2 eq en ax en	data	-81.4
	pred	-86.1
mn 2 eq acac ax acac	data	-110
	pred	-103
mn 2 eq en ax cn	data	-104
	pred	-109
mn 2 eq bipy ax h2o	data	-91.5
	pred	-90.3
mn 2 eq h2o ax h2o	data	-60.3
	pred	-63.7
mn 2 eq ncs ax nh3	data	-81.8
	pred	-90.3
mn 2 eq bipy ax bipy	data	-121
	pred	-112
mn 2 eq ox ax ox	data	-89.1
	pred	-85.3
mn 2 eq c2h3ns ax c2h3ns	data	-164
	pred	-141
mn 2 eq ncs ax co	data	-161
	pred	-131
mn 2 eq ncs ax ncs	data	-94.2
	pred	-98.7
mn 2 eq phen ax phen	data	-119
	pred	-110
mn 2 eq nh3 ax co	data	-147
	pred	-107
mn 2 eq en ax h2o	data	-64.6
	pred	-80.2
mn 2 eq en ax co	data	-156
	pred	-118
mn 2 eq nh3 ax pisc	data	-117
	pred	-105
mn 3 eq en ax co	data	-64.8
	pred	-71.5
mn 3 eq phen ax cl	data	-46.1
	pred	-57.5
mn 3 eq phen ax phen	data	-43.5
	pred	-56.9
mn 3 eq cn ax nh3	data	-45.7
	pred	-51.2
mn 3 eq pisc ax pisc	data	-90.5
	pred	-95.3
mn 3 eq ncs ax cl	data	-47.1
	pred	-54.3
mn 3 eq h2o ax co	data	-43.3
	pred	-45.8
mn 3 eq ox ax ox	data	-33
	pred	-43.9
mn 3 eq co ax co	data	-59.3
	pred	-89.7
mn 3 eq acac ax acac	data	-42.9
	pred	-48.6
mn 3 eq nh3 ax nh3	data	-35.6
	pred	-41.4
mn 3 eq nh3 ax co	data	-58.5
	pred	-61.7
mn 3 eq cn ax cn	data	-50.7
	pred	-80.9
mn 3 eq nh3 ax scn	data	-34.1
	pred	-66.8
mn 3 eq bipy ax bipy	data	-42.4
	pred	-58.8
mn 3 eq h2o ax pisc	data	-137
	pred	-46.1
mn 3 eq en ax en	data	-35
	pred	-42.8
mn 3 eq phen ax nh3	data	-36.5

	pred	-50.2
mn 3 eq h2o ax h2o	data	-30.8
	pred	-28.4
mn 3 eq c2h3ns ax c2h3ns	data	-62.7
	pred	-88.6
fe 2 eq co ax co	data	-174
	pred	-144
fe 2 eq acac ax acac	data	-117
	pred	-102
fe 2 eq tbuc ax co	data	-73
	pred	-97.6
fe 2 eq cn ax cn	data	-157
	pred	-135
fe 2 eq tbuc ax tbuc	data	-71
	pred	-75.1
fe 2 eq h2o ax ncs	data	-52.2
	pred	-65.8
fe 2 eq en ax en	data	-74.5
	pred	-86.7
fe 2 eq cl ax pisc	data	-117
	pred	-120
fe 2 eq ncs ax h2o	data	-69.4
	pred	-81.7
fe 2 eq pisc ax cl	data	-133
	pred	-131
fe 2 eq phen ax phen	data	-118
	pred	-108
fe 2 eq scn ax h2o	data	-49.1
	pred	-66.4
fe 2 eq nh3 ax nh3	data	-68.5
	pred	-73.5
fe 2 eq c2h3ns ax c2h3ns	data	-160
	pred	-142
fe 2 eq ox ax pisc	data	-121
	pred	-107
fe 2 eq pisc ax cn	data	-155
	pred	-144
fe 2 eq acac ax cn	data	-104
	pred	-107
fe 2 eq ox ax ox	data	-73.2
	pred	-80
fe 2 eq h2o ax h2o	data	-58.8
	pred	-68.4
fe 2 eq ncs ax pisc	data	-86.5
	pred	-127
fe 2 eq en ax co	data	-141
	pred	-111
fe 2 eq pisc ax ncs	data	-129
	pred	-122
fe 2 eq ncs ax co	data	-114
	pred	-124
fe 2 eq ncs ax ncs	data	-86.4
	pred	-97
fe 3 eq tbuc ax tbuc	data	-61
	pred	-61.7
fe 3 eq cn ax cn	data	-112
	pred	-107
fe 3 eq acac ax acac	data	-70.3
	pred	-72.4
fe 3 eq porphyrin ax h2o	data	-65.3
	pred	-70.1
fe 3 eq h2o ax cn	data	-50.6
	pred	-56.7
fe 3 eq porphyrin ax cn	data	-90.3
	pred	-94.1
fe 3 eq co ax co	data	-113
	pred	-113
fe 3 eq ox ax ox	data	-65.7
	pred	-63.8
fe 3 eq pisc ax h2o	data	-102
	pred	-86
fe 3 eq acac ax cl	data	-57
	pred	-62.9
fe 3 eq ox ax ncs	data	-72.3
	pred	-64.6
fe 3 eq en ax en	data	-67.1
	pred	-62.2
fe 3 eq pisc ax pisc	data	-133
	pred	-122
fe 3 eq bipy ax co	data	-147
	pred	-122
fe 3 eq pisc ax ncs	data	-94.5
	pred	-88.3
fe 3 eq ncs ax cl	data	-81.9
	pred	-79
fe 3 eq phen ax phen	data	-87.8
	pred	-82.6
fe 3 eq h2o ax h2o	data	-53.2
	pred	-42.6
fe 3 eq cl ax cl	data	-69.6

	pred	-69.1
fe 3 eq pisc ax cl	data	-73.9
	pred	-94.9
fe 3 eq c2h3ns ax c2h3ns	data	-112
	pred	-117
fe 3 eq nh3 ax scn	data	-64.4
	pred	-80.2
fe 3 eq acac ax ncs	data	-58.9
	pred	-61.1
fe 3 eq co ax scn	data	-90.1
	pred	-102
fe 3 eq bipy ax bipy	data	-75.2
	pred	-85.7
fe 3 eq ncs ax cn	data	-75.3
	pred	-108
fe 3 eq ncs ax ncs	data	-62.5
	pred	-75.4
fe 3 eq nh3 ax nh3	data	-75.9
	pred	-57.8
fe 3 eq cl ax pisc	data	-77.8
	pred	-84.6
co 2 eq ox ax ox	data	-41.8
	pred	-52.4
co 2 eq en ax en	data	-53
	pred	-56.2
co 2 eq tbuc ax tbuc	data	-36
	pred	-47.9
co 2 eq cl ax pisc	data	-71.9
	pred	-76.1
co 2 eq cn ax h2o	data	-52.9
	pred	-57
co 2 eq en ax pisc	data	-78.6
	pred	-82.4
co 2 eq phen ax h2o	data	-59.4
	pred	-63.5
co 2 eq phen ax phen	data	-65.8
	pred	-74.3
co 2 eq h2o ax ncs	data	-34.6
	pred	-40.7
co 2 eq c2h3ns ax c2h3ns	data	-102
	pred	-102
co 2 eq nh3 ax nh3	data	-50.4
	pred	-53.5
co 2 eq acac ax cn	data	-63.1
	pred	-85.3
co 2 eq nh3 ax h2o	data	-54.8
	pred	-42.4
co 2 eq acac ax acac	data	-39.6
	pred	-66.9
co 2 eq ncs ax ncs	data	-41.9
	pred	-66.7
co 2 eq bipy ax bipy	data	-65.8
	pred	-76.1
co 2 eq co ax co	data	-109
	pred	-103
co 2 eq en ax h2o	data	-56.3
	pred	-50.9
co 2 eq h2o ax h2o	data	-35.7
	pred	-35.7
co 3 eq co ax nh3	data	-98.4
	pred	-79.1
co 3 eq acac ax nh3	data	-42.8
	pred	-52.4
co 3 eq tbuc ax pisc	data	-80.1
	pred	-74.4
co 3 eq nh3 ax nh3	data	-60.4
	pred	-57.6
co 3 eq ox ax ox	data	-68.6
	pred	-53.1
co 3 eq en ax en	data	-51.3
	pred	-58.1
co 3 eq acac ax acac	data	-47.5
	pred	-55.8
co 3 eq phen ax phen	data	-76
	pred	-72.5
co 3 eq cn ax cn	data	-95.3
	pred	-89
co 3 eq c2h3ns ax c2h3ns	data	-87.7
	pred	-92.7
co 3 eq co ax co	data	-100
	pred	-94.2
co 3 eq tbuc ax tbuc	data	-31.7
	pred	-48.7
co 3 eq h2o ax h2o	data	-25.3
	pred	-36.8
co 3 eq ncs ax ncs	data	-23.2
	pred	-67.6
co 3 eq pisc ax pisc	data	-76.4
	pred	-94.8
co 3 eq bipy ax bipy	data	-74

	pred	-73.2
co 3 eq acac ax ncs	data	-61.9
	pred	-54.8
co 3 eq ox ax ncs	data	-66
	pred	-50.5
co 3 eq acac ax co	data	-86
	pred	-75.8
ni 2 eq tbuc ax tbuc	data	-60.6
	pred	-59.8
ni 2 eq bipy ax nh3	data	-57.9
	pred	-59.3
ni 2 eq en ax en	data	-56.1
	pred	-54.8
ni 2 eq co ax co	data	-67.2
	pred	-79.3
ni 2 eq nh3 ax nh3	data	-73.1
	pred	-55.2
ni 2 eq acac ax cn	data	-60
	pred	-60.4
ni 2 eq pisc ax pisc	data	-80.4
	pred	-86.1
ni 2 eq en ax h2o	data	-61.4
	pred	-57.2
ni 2 eq bipy ax bipy	data	-62.7
	pred	-60.7
ni 2 eq ncs ax ncs	data	-53.2
	pred	-59.5
ni 2 eq cn ax cn	data	-65.6
	pred	-69.8
ni 2 eq acac ax co	data	-52.4
	pred	-63.1
ni 2 eq c2h3ns ax c2h3ns	data	-62.1
	pred	-76.6
ni 2 eq acac ax nh3	data	-50.4
	pred	-51.1
ni 2 eq acac ax acac	data	-52.4
	pred	-55
ni 2 eq ox ax ox	data	-69.2
	pred	-59.5

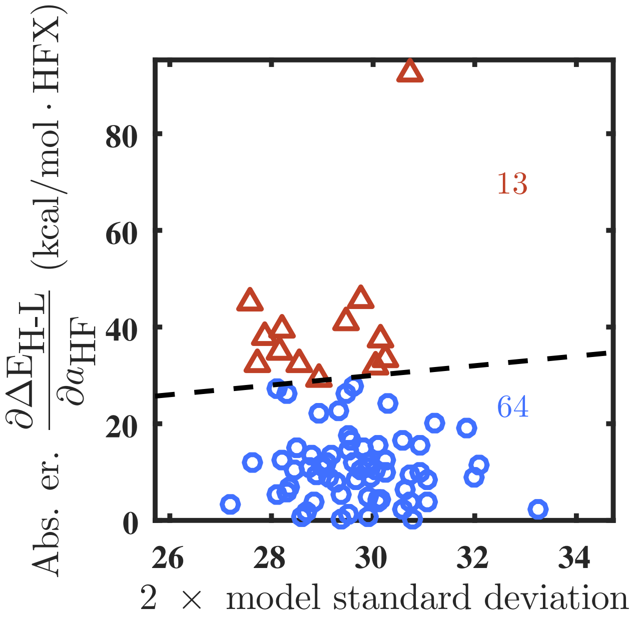

Figure S15: Parity plot for 2 standard deviation from the mean prediction and absolute prediction error for test case $\frac{\partial \Delta E_{H\text{-}L}}{\partial a_{HF}}$ prediction using ANN. All units are kcal/mol.HFX. The black line is y=x.

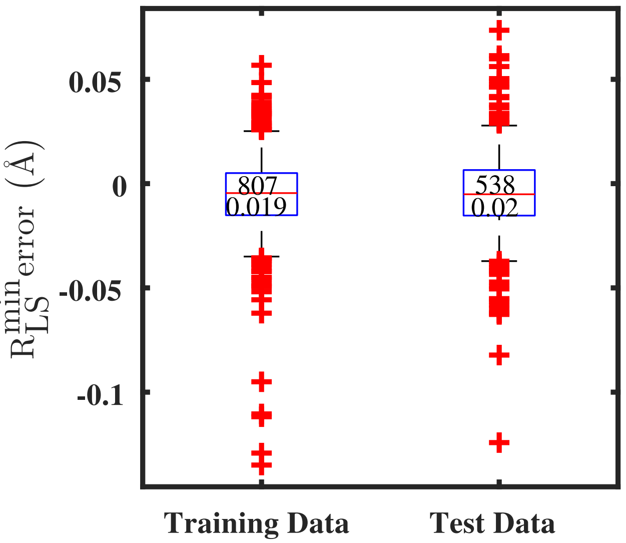

Figure S16: Error boxplot for regression of R_{LS}^{min} using an ANN, showing training and test data comparison. The top number indicates the number of trials, while the bottom indicates the RMSE.

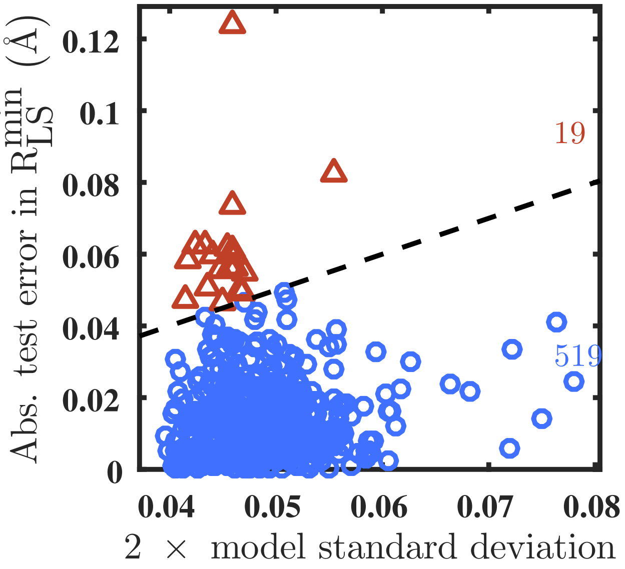

Figure S17: Parity plot for 2 standard deviation from the mean prediction and absolute prediction error for test case R_{LS}^{min} prediction using ANN. All units are Å. The black line is y=x.

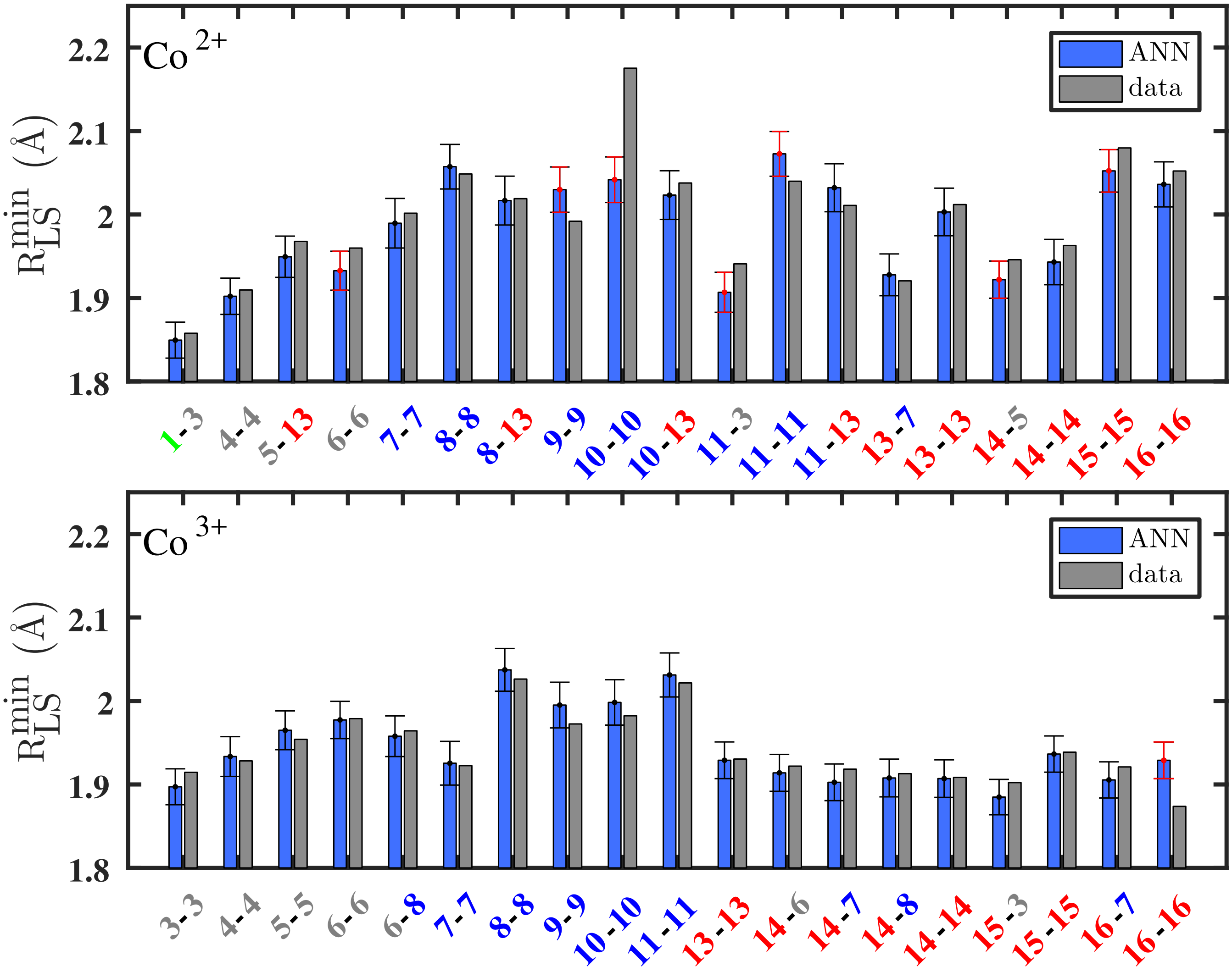

Figure S18: Model predictions of R_{LS}^{min} and data for low-spin Co using an ANN. The ligands are described by two numbers indicating the equatorial first and then the axial, color coded by ligand identity (green for halogen, gray for carbon, blue for nitrogen, and red for oxygen). The error bars represent an estimated 1 standard deviation from the mean prediction.

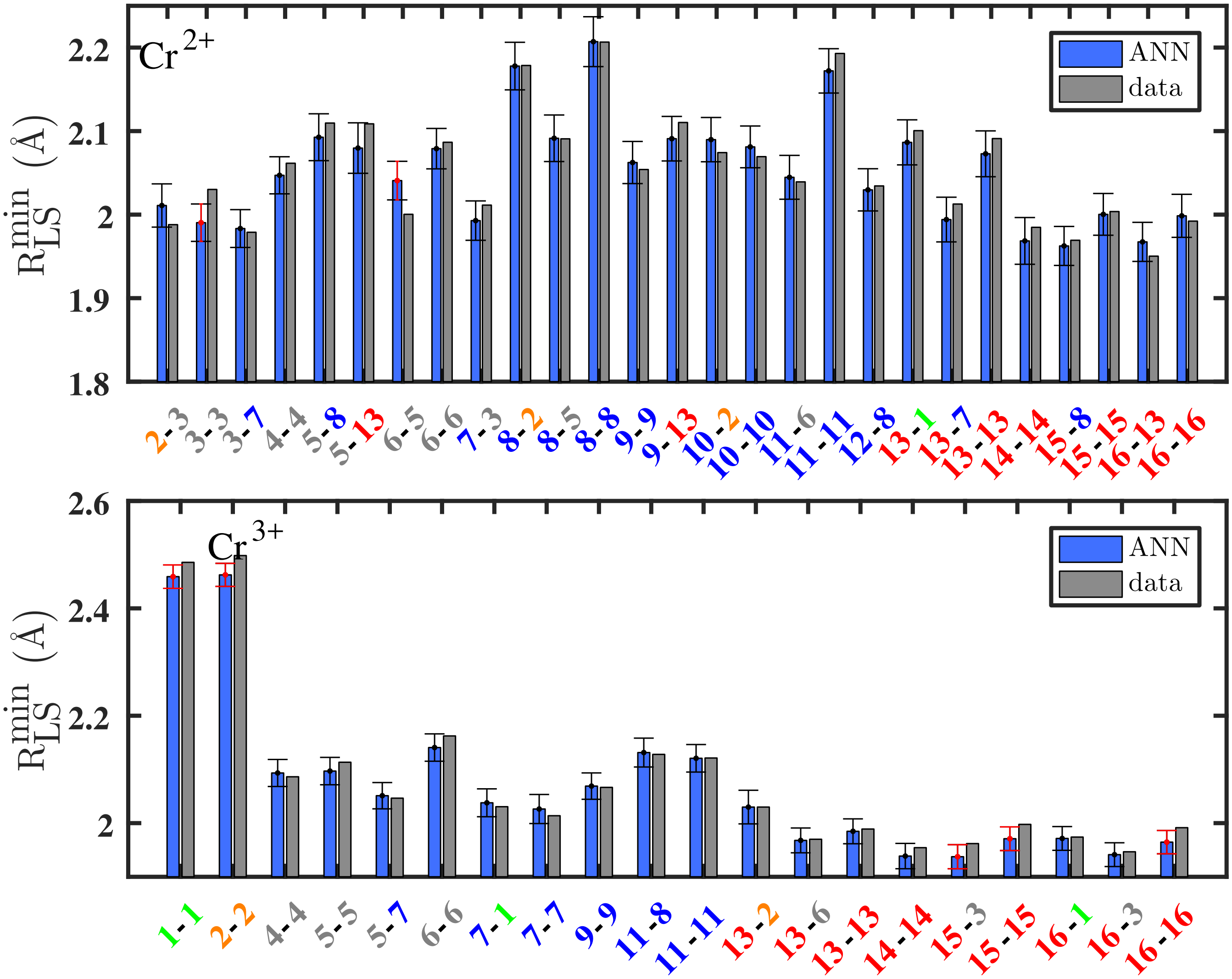

Figure S19: Model predictions of R_{LS}^{min} and data for low-spin Cr using an ANN. The ligands are described by two numbers indicating the equatorial first and then the axial, color coded by ligand identity (green for halogen, gray for carbon, blue for nitrogen, and red for oxygen). The error bars represent an estimated 1 standard deviation from the mean prediction.

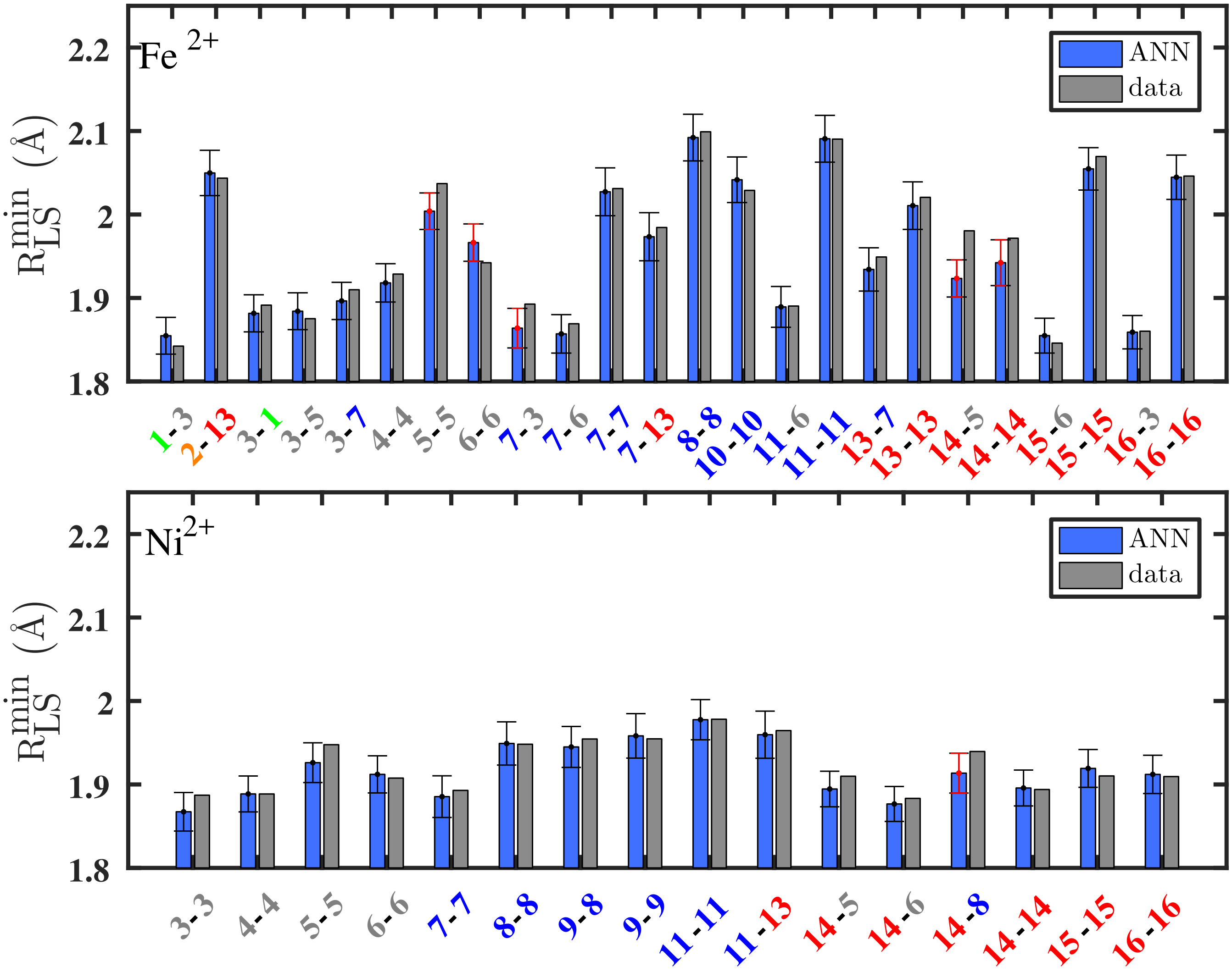

Figure S20: Model predictions of R_{LS}^{min} and data for low-spin Fe(II) (top) and Ni(II) (bottom) using an ANN. The ligands are described by two numbers indicating the equatorial first and then the axial, color coded by ligand identity (green for halogen, gray for carbon, blue for nitrogen, and red for oxygen). The error bars represent an estimated 1 standard deviation from the mean prediction.

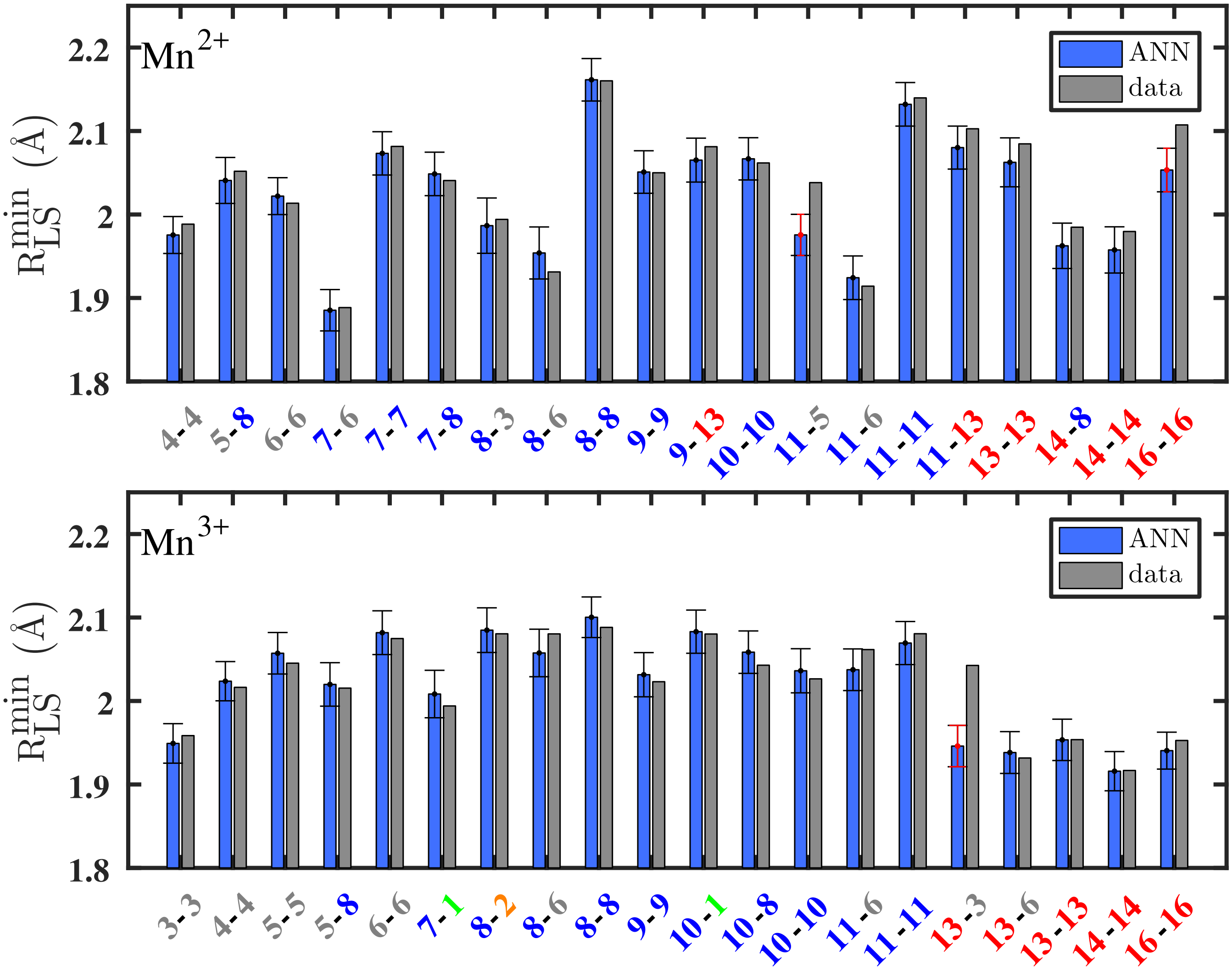

Figure S21: Model predictions of R_{LS}^{min} and data for low-spin Mn using an ANN. The ligands are described by two numbers indicating the equatorial first and then the axial, color coded by ligand identity (green for halogen, gray for carbon, blue for nitrogen, and red for oxygen). The error bars represent an estimated 1 standard deviation from the mean prediction.

Table S27: LS bond distance predictions and data in Å, compared for the full set of complexes. Each complex is named [metal oxidation axial ligand eq equatorial ligand]. For each complex, the first row has the data obtained from DFT using varying fractions of exact exchange (a_{HF}) and the second row has the ANN predictions

		a_{HF}						
	type	0	0.05	0.1	0.15	0.2	0.25	0.30
cr 2 eq h2o ax ncs	data	1.98	1.99	2	2.01	2.01	2.02	2.03
	pred	1.97	1.98	1.98	1.99	1.99	2	2.01
cr 2 eq porphyrin ax nh3	data	2.04	2.04	2.04	2.04	2.03	2.03	2.03
	pred	2.02	2.02	2.03	2.03	2.03	2.03	2.04
cr 2 eq scn ax pisc	data	1.96	1.97	1.97	1.98	1.99	2	2
	pred	1.97	1.98	1.99	2	2.01	2.03	2.04
cr 2 eq ncs ax pisc	data	1.99	2	2	2	2.01	2.02	2.03
	pred	1.96	1.97	1.98	1.99	1.99	2	2
cr 2 eq cn ax nh3	data	2.09	2.08	2.09	2.1	2.11	2.12	2.13
	pred	2.06	2.07	2.08	2.08	2.09	2.1	2.11
cr 2 eq tbuc ax tbuc	data	2.02	2.01	2.01	2.01	2	2	2
	pred	1.99	1.99	2	2	2	2	2.01
cr 2 eq phen ax scn	data	2.07	2.07	2.08	2.07	2.07	2.08	2.09
	pred	2.07	2.08	2.08	2.09	2.09	2.09	2.1
cr 2 eq en ax co	data	2	2	2.01	2.03	2.04	2.05	2.07
	pred	1.98	2	2.02	2.03	2.04	2.06	2.07
cr 2 eq bipy ax bipy	data	2.04	2.04	2.04	2.04	2.05	2.07	2.08
	pred	2.05	2.05	2.05	2.06	2.06	2.07	2.07
cr 2 eq en ax en	data	2.18	2.19	2.19	2.19	2.19	2.19	2.2
	pred	2.17	2.17	2.17	2.17	2.17	2.17	2.18
cr 2 eq co ax cn	data	1.97	1.98	1.99	1.99	2	2.01	2.02
	pred	2.02	2.03	2.03	2.04	2.04	2.04	2.05
cr 2 eq pisc ax pisc	data	1.99	2	2.01	2.02	2.03	2.04	2.05
	pred	1.96	1.97	1.98	1.98	1.99	2	2
cr 2 eq ox ax h2o	data	1.97	1.96	1.96	1.95	1.95	-	-
	pred	1.96	1.96	1.96	1.96	1.97		
cr 2 eq h2o ax cl	data	2.11	2.1	2.1	2.1	2.1	2.1	2.1
	pred	2.09	2.09	2.09	2.09	2.09	2.09	2.09
cr 2 eq nh3 ax scn	data	2.17	2.18	2.18	2.18	2.18	2.18	2.18
	pred	2.17	2.17	2.17	2.18	2.18	2.18	2.18
cr 2 eq ox ax ox	data	2.01	2	2	2	1.99	1.99	1.98
	pred	1.99	1.99	1.99	2	2	2	2
cr 2 eq h2o ax h2o	data	2.1	2.09	2.09	2.09	2.09	2.09	2.09
	pred	2.06	2.07	2.07	2.07	2.07	2.07	2.08
cr 2 eq cn ax h2o	data	2.08	2.09	2.1	2.1	2.11	2.11	2.12
	pred	2.06	2.06	2.07	2.07	2.08	2.09	2.09
cr 2 eq pisc ax ncs	data	-	1.96	1.97	1.97	1.98	1.98	1.99
	pred		1.97	1.97	1.98	1.98	1.99	2
cr 2 eq acac ax acac	data	1.95	1.95	1.95	1.96	1.98	2	2.01
	pred	1.95	1.95	1.96	1.96	1.97	1.98	1.98
cr 2 eq bipy ax h2o	data	2.08	2.09	2.09	2.09	2.11	2.12	2.12
	pred	2.06	2.07	2.08	2.08	2.09	2.1	2.1
cr 2 eq nh3 ax nh3	data	2.2	2.2	2.2	2.21	2.21	2.21	2.22
	pred	2.2	2.2	2.2	2.21	2.21	2.21	2.21
cr 2 eq co ax co	data	2.05	2.06	2.07	2.08	2.09	2.1	2.11
	pred	2.06	2.06	2.07	2.07	2.08	2.08	2.09
cr 2 eq c2h3ns ax c2h3ns	data	2.04	2.05	2.05	2.06	2.06	2.07	2.08
	pred	2.02	2.03	2.04	2.04	2.05	2.05	2.06
cr 2 eq phen ax phen	data	2.05	2.05	2.06	2.06	2.07	2.09	2.09
	pred	2.07	2.07	2.07	2.08	2.08	2.09	2.09
cr 2 eq tbuc ax nh3	data	1.98	1.98	1.98	1.97	1.97	1.97	1.96
	pred	1.95	1.96	1.96	1.96	1.96	1.97	1.97
cr 2 eq nh3 ax cn	data	2.05	2.06	2.07	2.08	2.09	2.1	2.1
	pred	2.04	2.05	2.07	2.08	2.09	2.1	2.11
cr 3 eq c2h3ns ax c2h3ns	data	2.07	2.07	2.08	2.08	2.09	2.09	2.09
	pred	2.07	2.07	2.08	2.09	2.09	2.1	2.11
cr 3 eq acac ax acac	data	1.96	1.96	1.96	1.96	1.95	1.95	1.96
	pred	1.92	1.93	1.93	1.93	1.94	1.94	1.95
cr 3 eq h2o ax scn	data	2.05	2.04	2.04	2.03	2.03	2.03	2.02
	pred	2.03	2.03	2.03	2.03	2.03	2.03	2.04
cr 3 eq en ax nh3	data	2.15	2.14	2.14	2.13	2.13	2.12	2.12
	pred	2.13	2.13	2.13	2.13	2.13	2.13	2.14
cr 3 eq scn ax scn	data	2.51	2.5	2.5	2.5	2.5	2.49	2.49
	pred	2.46	2.46	2.46	2.46	2.46	2.46	2.46
cr 3 eq en ax en	data	2.14	2.13	2.13	2.13	2.12	2.12	2.12
	pred	2.12	2.12	2.12	2.12	2.12	2.12	2.12
cr 3 eq cn ax cn	data	2.1	2.11	2.11	2.11	2.11	2.11	2.12
	pred	2.08	2.08	2.09	2.09	2.1	2.1	2.1
cr 3 eq cn ax ncs	data	2.03	2.03	2.04	2.04	2.05	-	-
	pred	2.04	2.04	2.04	2.05	2.05		
cr 3 eq tbuc ax tbuc	data	-	-	2.01	2	2	2	2
	pred			1.97	1.97	1.97	1.97	1.97
cr 3 eq ncs ax ncs	data	2.01	2.01	2.01	2.01	2.01	2.02	2.02
	pred	2.01	2.02	2.02	2.02	2.03	2.03	2.03
cr 3 eq ox ax ox	data	2	2	2	1.99	1.99	1.99	1.98
	pred	1.96	1.96	1.96	1.96	1.96	1.97	1.97
cr 3 eq cl ax cl	data	2.5	2.49	2.48	2.49	2.49	2.48	2.48
	pred	2.46	2.46	2.46	2.46	2.46	2.46	2.46

cr 3 eq co ax co	data	2.15	2.15	2.16	2.16	2.16	2.16	2.17
	pred	2.13	2.13	2.13	2.14	2.14	2.14	2.15
cr 3 eq h2o ax co	data	1.98	1.98	1.98	1.97	1.97	1.97	1.97
	pred	1.96	1.96	1.97	1.97	1.97	1.97	1.97
cr 3 eq bipy ax bipy	data	2.07	2.07	2.07	2.07	2.07	2.07	2.07
	pred	2.06	2.06	2.07	2.07	2.07	2.07	2.07
cr 3 eq ncs ax cl	data	2.02	2.02	2.03	2.03	2.03	2.03	2.03
	pred	2.02	2.02	2.03	2.03	2.04	2.04	2.05
cr 3 eq tbuc ax pisc	data	1.96	1.96	1.96	1.97	1.96	1.96	1.95
	pred	1.93	1.93	1.93	1.94	1.94	1.94	1.94
cr 3 eq ox ax cl	data	1.99	1.99	1.98	1.98	1.97	1.97	1.97
	pred	1.97	1.97	1.97	1.97	1.97	1.97	1.98
cr 3 eq h2o ax h2o	data	2	2	1.99	1.99	1.99	1.99	1.98
	pred	1.98	1.98	1.98	1.98	1.98	1.99	1.99
cr 3 eq ox ax pisc	data	1.95	1.95	1.95	1.95	1.95	1.94	1.94
	pred	1.93	1.94	1.94	1.94	1.94	1.94	1.94
mn 2 eq acac ax nh3	data	1.93	1.94	1.95	1.96	1.98	2.01	2.02
	pred	1.95	1.95	1.95	1.96	1.96	1.97	1.98
mn 2 eq nh3 ax nh3	data	2.15	2.15	2.16	2.16	2.16	2.16	2.16
	pred	2.13	2.14	2.15	2.16	2.16	2.17	2.17
mn 2 eq co ax co	data	1.98	1.98	1.99	2	2.01	2.03	2.04
	pred	1.99	2	2.01	2.01	2.02	2.03	2.04
mn 2 eq cn ax nh3	data	2.01	2.02	2.03	2.04	2.05	2.06	2.08
	pred	1.98	2	2.01	2.03	2.04	2.05	2.07
mn 2 eq en ax en	data	2.13	2.13	2.14	2.14	2.14	2.15	2.14
	pred	2.11	2.12	2.12	2.13	2.13	2.14	2.14
mn 2 eq acac ax acac	data	1.93	1.95	1.96	1.97	1.98	1.99	1.99
	pred	1.93	1.94	1.94	1.95	1.96	1.97	1.98
mn 2 eq en ax cn	data	2	2.01	2.02	2.03	2.04	2.04	2.04
	pred	1.94	1.95	1.95	1.96	1.98	1.99	2
mn 2 eq bipy ax h2o	data	2.04	2.05	2.07	2.07	2.08	2.09	2.09
	pred	2.03	2.04	2.05	2.06	2.07	2.07	2.08
mn 2 eq h2o ax h2o	data	2.06	2.06	2.06	2.07	2.08	2.09	2.09
	pred	2.04	2.05	2.05	2.06	2.06	2.07	2.07
mn 2 eq ncs ax nh3	data	1.99	2	2.02	2.03	2.04	2.05	2.06
	pred	1.99	2	2.02	2.03	2.05	2.06	2.08
mn 2 eq bipy ax bipy	data	2.02	2.03	2.03	2.05	2.05	2.06	2.06
	pred	2.02	2.03	2.04	2.04	2.05	2.06	2.07
mn 2 eq ox ax ox	data	2.03	2.05	2.08	2.09	2.11	-	-
	pred	2.03	2.04	2.05	2.05	2.05		
mn 2 eq c2h3ns ax c2h3ns	data	1.96	1.97	1.97	1.98	1.99	2	2.01
	pred	1.95	1.96	1.96	1.97	1.98	1.98	1.99
mn 2 eq ncs ax co	data	1.86	1.87	1.87	1.88	1.89	1.9	1.91
	pred	1.86	1.86	1.87	1.88	1.89	1.9	1.91
mn 2 eq ncs ax ncs	data	2.03	2.04	2.05	2.07	2.08	2.1	2.14
	pred	2.03	2.04	2.05	2.06	2.07	2.08	2.09
mn 2 eq phen ax phen	data	2.03	2.04	2.05	2.05	2.06	2.05	2.06
	pred	2.04	2.04	2.05	2.06	2.07	2.08	2.08
mn 2 eq nh3 ax co	data	1.89	1.9	1.91	1.92	1.93	1.95	1.96
	pred	1.89	1.9	1.91	1.93	1.95	1.98	2
mn 2 eq en ax h2o	data	2.09	2.09	2.09	2.1	2.1	2.1	2.1
	pred	2.05	2.06	2.07	2.08	2.08	2.08	2.09
mn 2 eq en ax co	data	1.88	1.88	1.89	1.9	1.91	1.93	1.94
	pred	1.9	1.9	1.91	1.91	1.92	1.94	1.95
mn 2 eq nh3 ax pisc	data	1.94	1.95	1.96	1.97	1.99	2.01	2.02
	pred	1.92	1.93	1.95	1.97	1.99	2	2.02
mn 3 eq en ax co	data	2.01	2.02	2.04	2.05	2.06	2.07	2.06
	pred	2	2.01	2.02	2.03	2.04	2.04	2.05
mn 3 eq phen ax cl	data	2.08	2.08	2.08	2.08	2.08	2.08	2.08
	pred	2.07	2.07	2.07	2.08	2.08	2.09	2.1
mn 3 eq phen ax phen	data	2.03	2.03	2.03	2.03	2.03	2.02	2.02
	pred	2.03	2.03	2.03	2.04	2.04	2.04	2.04
mn 3 eq cn ax nh3	data	2	2.01	2.01	2.01	2.02	2.02	2.02
	pred	2	2.01	2.01	2.02	2.02	2.02	2.03
mn 3 eq pisc ax pisc	data	1.92	1.93	1.94	1.95	1.96	1.97	1.98
	pred	1.92	1.93	1.94	1.94	1.95	1.95	1.96
mn 3 eq ncs ax cl	data	1.98	1.98	1.99	1.99	1.99	2	2
	pred	1.98	1.99	1.99	2	2.01	2.02	2.03
mn 3 eq h2o ax co	data	1.95	1.95	1.94	1.94	1.93	1.93	1.93
	pred	1.93	1.94	1.94	1.94	1.94	1.94	1.94
mn 3 eq ox ax ox	data	1.97	1.97	1.96	1.96	1.95	1.95	1.94
	pred	1.94	1.94	1.94	1.94	1.94	1.94	1.94
mn 3 eq co ax co	data	2.05	2.06	2.06	2.07	2.07	2.08	2.08
	pred	2.06	2.06	2.07	2.08	2.08	2.09	2.09
mn 3 eq acac ax acac	data	1.91	1.92	1.92	1.9	1.92	1.92	1.92
	pred	1.91	1.91	1.91	1.91	1.92	1.92	1.92
mn 3 eq nh3 ax nh3	data	2.11	2.1	2.1	2.09	2.09	2.08	2.08
	pred	2.1	2.1	2.1	2.1	2.1	2.1	2.1
mn 3 eq nh3 ax co	data	2.03	2.04	2.06	2.07	2.08	2.08	2.08
	pred	2.02	2.04	2.04	2.05	2.06	2.06	2.07
mn 3 eq cn ax cn	data	2.03	2.03	2.04	2.04	2.05	2.05	2.05
	pred	2.03	2.04	2.05	2.05	2.06	2.06	2.07
mn 3 eq nh3 ax scn	data	2.09	2.09	2.09	2.08	2.08	2.08	2.08
	pred	2.09	2.09	2.08	2.08	2.08	2.09	2.09
mn 3 eq bipy ax bipy	data	2.02	2.02	2.02	2.02	2.02	2.02	2.02
	pred	2.03	2.03	2.03	2.03	2.03	2.03	2.03
mn 3 eq h2o ax pisc	data	1.97	1.97	1.98	2.03	2.04	2.06	-
	pred	1.93	1.94	1.94	1.94	1.95	1.95	
mn 3 eq en ax en	data	2.08	2.08	2.08	2.08	2.08	2.08	2.07
	pred	2.07	2.07	2.07	2.07	2.07	2.07	2.07

mn 3 eq phen ax nh3	data	2.06	2.05	2.05	2.04	2.04	2.04	2.04
	pred	2.05	2.05	2.06	2.06	2.06	2.06	2.06
mn 3 eq h2o ax h2o	data	1.97	1.96	1.96	1.96	1.95	1.95	1.95
	pred	1.95	1.95	1.95	1.95	1.95	1.96	1.96
mn 3 eq c2h3ns ax c2h3ns	data	1.99	2	2.01	2.01	2.02	2.02	2.02
	pred	2	2.01	2.01	2.02	2.02	2.03	2.04
fe 2 eq co ax co	data	1.92	1.92	1.93	1.93	1.94	1.95	1.96
	pred	1.94	1.95	1.95	1.96	1.97	1.97	1.98
fe 2 eq acac ax acac	data	1.94	1.94	1.95	1.95	1.97	1.97	1.98
	pred	1.92	1.93	1.93	1.94	1.94	1.95	1.96
fe 2 eq tbuc ax co	data	1.82	1.82	1.83	1.84	1.85	1.86	1.87
	pred	1.85	1.85	1.85	1.85	1.85	1.86	1.86
fe 2 eq cn ax cn	data	2	2	2.01	2.02	2.04	2.05	2.07
	pred	1.96	1.97	1.98	1.99	2	2.01	2.02
fe 2 eq tbuc ax tbuc	data	2.04	2.05	2.06	2.06	2.07	2.07	-
	pred	2.04	2.04	2.05	2.05	2.05	2.06	
fe 2 eq h2o ax ncs	data	1.93	1.93	1.94	1.94	1.95	1.95	1.96
	pred	1.91	1.92	1.92	1.93	1.93	1.94	1.95
fe 2 eq en ax en	data	2.08	2.08	2.09	2.09	2.09	2.09	2.09
	pred	2.06	2.07	2.08	2.09	2.09	2.1	2.1
fe 2 eq cl ax pisc	data	1.81	1.82	1.82	1.83	1.84	1.86	1.87
	pred	1.85	1.85	1.85	1.85	1.85	1.86	1.86
fe 2 eq ncs ax h2o	data	1.93	1.94	1.95	1.98	1.98	1.99	2
	pred	1.95	1.95	1.96	1.96	1.97	1.98	1.99
fe 2 eq pisc ax cl	data	1.87	1.87	1.88	1.88	1.89	1.9	1.91
	pred	1.86	1.86	1.87	1.87	1.88	1.89	1.9
fe 2 eq phen ax phen	data	2.01	2.01	2.02	2.02	2.03	2.03	2.04
	pred	2.01	2.02	2.03	2.03	2.04	2.05	2.06
fe 2 eq scn ax h2o	data	2.05	2.05	2.04	2.04	2.04	2.04	2.04
	pred	2.04	2.04	2.05	2.05	2.05	2.05	2.05
fe 2 eq nh3 ax nh3	data	2.09	2.09	2.09	2.1	2.1	2.1	2.1
	pred	2.05	2.06	2.08	2.08	2.09	2.1	2.1
fe 2 eq c2h3ns ax c2h3ns	data	1.91	1.91	1.92	1.92	1.93	1.94	1.94
	pred	1.9	1.91	1.91	1.91	1.92	1.92	1.93
fe 2 eq ox ax pisc	data	1.83	1.83	1.84	1.85	1.86	1.87	1.89
	pred	1.85	1.85	1.85	1.86	1.86	1.86	1.87
fe 2 eq pisc ax cn	data	1.86	1.86	1.87	1.87	1.88	1.88	1.89
	pred	1.87	1.87	1.88	1.88	1.88	1.89	1.89
fe 2 eq acac ax cn	data	1.94	1.94	1.96	1.96	1.98	1.99	1.99
	pred	1.92	1.92	1.92	1.92	1.92	1.92	1.93
fe 2 eq ox ax ox	data	2.03	2.04	2.04	2.04	2.05	2.05	2.05
	pred	2.01	2.02	2.03	2.04	2.04	2.05	2.05
fe 2 eq h2o ax h2o	data	2.03	2.03	2.02	2.02	2.02	2.02	2.02
	pred	1.99	2	2	2.01	2.01	2.01	2.02
fe 2 eq ncs ax pisc	data	1.85	1.86	1.87	1.88	1.89	-	1.92
	pred	1.85	1.85	1.85	1.86	1.86		1.88
fe 2 eq en ax co	data	1.85	1.86	1.87	1.88	1.89	1.91	1.92
	pred	1.87	1.87	1.88	1.88	1.89	1.9	1.91
fe 2 eq pisc ax ncs	data	1.88	1.89	1.89	1.9	1.91	1.92	1.93
	pred	1.87	1.88	1.88	1.89	1.9	1.9	1.91
fe 2 eq ncs ax co	data	1.84	1.84	1.85	1.86	1.87	1.88	1.9
	pred	1.85	1.85	1.85	1.85	1.86	1.86	1.87
fe 2 eq ncs ax ncs	data	1.99	2	2.01	2.03	2.03	2.04	2.05
	pred	1.98	2	2.01	2.02	2.03	2.04	2.04
fe 3 eq tbuc ax tbuc	data	1.96	1.96	1.96	1.95	1.95	1.95	1.94
	pred	1.93	1.94	1.94	1.94	1.94	1.94	1.94
fe 3 eq cn ax cn	data	1.98	1.99	1.99	1.99	2	2	2
	pred	1.98	1.99	1.99	2	2	2.01	2.01
fe 3 eq acac ax acac	data	1.92	1.92	1.92	1.91	1.91	1.91	1.9
	pred	1.91	1.91	1.91	1.91	1.91	1.91	1.91
fe 3 eq porphyrin ax h2o	data	1.99	1.99	1.98	1.98	2.03	2.03	2.03
	pred	1.99	1.99	1.99	1.99	1.99	2	2
fe 3 eq h2o ax cn	data	1.94	1.94	1.95	1.95	1.95	1.95	1.95
	pred	1.92	1.92	1.92	1.92	1.93	1.93	1.93
fe 3 eq porphyrin ax cn	data	1.98	1.98	1.98	1.98	1.99	1.99	1.99
	pred	1.96	1.96	1.97	1.97	1.97	1.98	1.98
fe 3 eq co ax co	data	2.01	2.01	2.02	2.02	2.03	2.03	2.03
	pred	2	2	2.01	2.01	2.02	2.03	2.03
fe 3 eq ox ax ox	data	1.95	1.95	1.95	1.94	1.94	1.93	1.93
	pred	1.93	1.93	1.93	1.93	1.93	1.93	1.94
fe 3 eq pisc ax h2o	data	1.92	1.92	1.93	1.94	1.95	1.96	1.97
	pred	1.91	1.91	1.91	1.91	1.91	1.91	1.91
fe 3 eq acac ax cl	data	1.95	1.95	1.95	1.94	1.94	1.94	1.93
	pred	1.93	1.93	1.93	1.93	1.93	1.93	1.93
fe 3 eq ox ax ncs	data	1.95	1.95	1.94	1.94	1.93	1.92	1.92
	pred	1.9	1.9	1.9	1.9	1.91	1.91	1.91
fe 3 eq en ax en	data	2.07	2.07	2.06	2.05	2.05	2.05	2.05
	pred	2.05	2.05	2.05	2.05	2.05	2.05	2.05
fe 3 eq pisc ax pisc	data	1.91	1.91	1.91	1.92	1.92	1.92	1.94
	pred	1.9	1.9	1.9	1.91	1.91	1.91	1.91
fe 3 eq bipy ax co	data	1.89	1.9	1.9	1.9	1.92	1.93	1.95
	pred	1.89	1.9	1.91	1.92	1.92	1.93	1.94
fe 3 eq pisc ax ncs	data	1.9	1.9	1.9	1.89	1.89	1.89	1.88
	pred	1.91	1.91	1.91	1.91	1.92	1.92	1.92
fe 3 eq ncs ax cl	data	1.94	1.95	1.95	1.95	1.95	1.95	1.96
	pred	1.94	1.94	1.95	1.95	1.96	1.96	1.97
fe 3 eq phen ax phen	data	2.01	2.01	2.01	2.01	2	2	2
	pred	2.01	2.01	2.01	2.01	2.01	2.01	2.01
fe 3 eq h2o ax h2o	data	1.94	1.94	1.94	1.94	1.94	1.93	1.93
	pred	1.93	1.93	1.93	1.93	1.93	1.93	1.93

fe 3 eq cl ax cl	data	2.46	2.46	2.46	2.45	2.45	2.45	2.46
	pred	2.44	2.44	2.45	2.45	2.45	2.45	2.45
fe 3 eq pisc ax cl	data	1.92	1.93	1.93	1.94	1.94	1.95	1.96
	pred	1.9	1.91	1.91	1.92	1.92	1.93	1.94
fe 3 eq c2h3ns ax c2h3ns	data	1.95	1.96	1.96	1.96	1.97	1.97	1.98
	pred	1.94	1.95	1.95	1.95	1.96	1.96	1.96
fe 3 eq nh3 ax scn	data	2.06	2.06	2.05	2.05	2.05	-	-
	pred	2.05	2.05	2.05	2.05	2.05		
fe 3 eq acac ax ncs	data	1.88	1.88	1.88	1.88	1.88	1.88	1.89
	pred	1.9	1.9	1.9	1.9	1.9	1.9	1.9
fe 3 eq co ax scn	data	-	-	1.88	1.89	1.9	1.9	1.91
	pred			1.93	1.93	1.93	1.94	1.94
fe 3 eq bipy ax bipy	data	2	2	2	2	2	2	2
	pred	2	2	2	2	2	2	2
fe 3 eq ncs ax cn	data	1.94	1.95	1.95	1.95	1.96	1.96	1.96
	pred	1.91	1.92	1.92	1.93	1.93	1.93	1.94
fe 3 eq ncs ax ncs	data	1.94	1.94	1.94	1.94	1.94	1.94	1.94
	pred	1.94	1.94	1.94	1.94	1.95	1.95	1.95
fe 3 eq nh3 ax nh3	data	2.08	2.07	2.07	2.06	2.06	2.06	2.05
	pred	2.06	2.06	2.06	2.06	2.06	2.06	2.06
fe 3 eq cl ax pisc	data	1.86	1.87	1.88	1.89	1.9	1.92	1.93
	pred	1.87	1.88	1.89	1.9	1.9	1.91	1.91
co 2 eq ox ax ox	data	2.02	2.04	2.04	2.05	2.05	2.06	2.05
	pred	2.01	2.01	2.02	2.03	2.04	2.04	2.05
co 2 eq en ax en	data	2.04	2.04	2.04	2.04	2.04	2.05	2.06
	pred	2.04	2.05	2.06	2.07	2.07	2.08	2.08
co 2 eq tbuc ax tbuc	data	2.06	2.07	2.07	2.08	2.08	2.08	2.05
	pred	2.03	2.04	2.04	2.05	2.05	2.05	2.06
co 2 eq cl ax pisc	data	1.82	1.82	1.83	1.84	1.86	1.87	1.89
	pred	1.85	1.85	1.85	1.85	1.85	1.85	1.85
co 2 eq cn ax h2o	data	1.94	1.95	1.95	1.96	1.97	1.98	1.98
	pred	1.93	1.93	1.94	1.94	1.95	1.95	1.96
co 2 eq en ax pisc	data	1.89	1.9	1.91	1.93	1.94	1.96	1.97
	pred	1.89	1.89	1.9	1.9	1.91	1.91	1.92
co 2 eq phen ax h2o	data	2.02	2.02	2.03	2.03	2.04	2.05	2.06
	pred	2	2.01	2.01	2.02	2.02	2.03	2.03
co 2 eq phen ax phen	data	1.98	1.99	1.99	2.17	2.18	2.18	2.18
	pred	1.99	2	2.02	2.03	2.04	2.05	2.06
co 2 eq h2o ax ncs	data	1.92	1.92	1.91	1.92	1.92	1.93	1.93
	pred	1.91	1.92	1.92	1.92	1.93	1.93	1.93
co 2 eq c2h3ns ax c2h3ns	data	1.91	1.89	1.89	1.9	1.91	1.92	1.92
	pred	1.89	1.89	1.9	1.9	1.9	1.91	1.91
co 2 eq nh3 ax nh3	data	2.04	2.04	2.04	2.04	2.05	2.06	2.07
	pred	2.01	2.02	2.03	2.05	2.06	2.07	2.07
co 2 eq acac ax cn	data	1.93	1.93	1.94	1.94	1.95	1.95	1.96
	pred	1.91	1.91	1.92	1.92	1.92	1.92	1.93
co 2 eq nh3 ax h2o	data	2.02	2.02	2.02	2.02	2.02	2.02	2.02
	pred	2	2	2.01	2.01	2.02	2.02	2.03
co 2 eq acac ax acac	data	1.92	1.93	1.94	1.96	1.96	1.97	1.97
	pred	1.93	1.93	1.93	1.94	1.94	1.95	1.95
co 2 eq ncs ax ncs	data	1.93	1.96	1.97	1.99	2	2.01	2.02
	pred	1.92	1.94	1.96	1.97	1.99	2	2.02
co 2 eq bipy ax bipy	data	1.97	1.97	1.98	1.99	1.99	2	1.99
	pred	1.99	2	2.01	2.02	2.03	2.04	2.05
co 2 eq co ax co	data	1.92	1.93	1.94	1.95	1.96	1.97	1.95
	pred	1.91	1.92	1.92	1.93	1.93	1.94	1.95
co 2 eq en ax h2o	data	2.01	2.02	2.01	2.01	2.01	2.01	2.01
	pred	2.01	2.02	2.02	2.03	2.03	2.04	2.04
co 2 eq h2o ax h2o	data	2.01	2.01	2.01	2.01	2.01	2.01	2.01
	pred	1.99	1.99	2	2	2	2.01	2.01
co 3 eq co ax nh3	data	-	1.94	1.95	1.96	1.96	1.97	1.98
	pred		1.94	1.95	1.95	1.96	1.96	1.97
co 3 eq acac ax nh3	data	1.93	1.93	1.92	1.92	1.91	1.91	1.9
	pred	1.91	1.91	1.91	1.91	1.91	1.91	1.91
co 3 eq tbuc ax pisc	data	1.87	1.88	1.89	1.89	1.9	1.91	1.91
	pred	1.87	1.88	1.88	1.88	1.88	1.89	1.89
co 3 eq nh3 ax nh3	data	2.05	2.04	2.04	2.03	2.03	2.02	2.02
	pred	2.03	2.03	2.03	2.03	2.04	2.04	2.04
co 3 eq ox ax ox	data	1.87	1.88	1.88	1.87	1.87	1.87	1.86
	pred	1.92	1.92	1.92	1.93	1.93	1.93	1.93
co 3 eq en ax en	data	2.04	2.03	2.03	2.02	2.02	2.02	2.01
	pred	2.03	2.03	2.03	2.03	2.03	2.03	2.03
co 3 eq acac ax acac	data	1.93	1.93	1.92	1.92	1.91	1.9	1.9
	pred	1.91	1.91	1.91	1.91	1.91	1.91	1.91
co 3 eq phen ax phen	data	1.99	1.99	1.99	1.98	1.98	1.98	1.98
	pred	1.99	1.99	2	2	2	2	2
co 3 eq cn ax cn	data	1.95	1.95	1.95	1.95	1.95	1.96	1.96
	pred	1.94	1.95	1.95	1.96	1.96	1.97	1.98
co 3 eq c2h3ns ax c2h3ns	data	1.92	1.92	1.92	1.93	1.93	1.93	1.93
	pred	1.92	1.92	1.93	1.93	1.93	1.94	1.94
co 3 eq co ax co	data	1.97	1.97	1.97	1.97	1.98	1.98	1.98
	pred	1.96	1.96	1.97	1.97	1.98	1.98	1.99
co 3 eq tbuc ax tbuc	data	1.96	1.96	1.95	1.94	1.94	1.93	1.93
	pred	1.93	1.93	1.93	1.93	1.94	1.94	1.94
co 3 eq h2o ax h2o	data	1.96	1.95	1.95	1.94	1.93	1.92	1.92
	pred	1.93	1.93	1.93	1.93	1.93	1.93	1.93
co 3 eq ncs ax ncs	data	1.92	1.92	1.92	1.92	1.92	1.92	1.92
	pred	1.92	1.92	1.92	1.92	1.93	1.93	1.93
co 3 eq pisc ax pisc	data	1.91	1.91	1.91	1.91	1.91	1.91	1.92
	pred	1.88	1.89	1.89	1.89	1.9	1.9	1.9

co 3 eq bipy ax bipy	data	1.98	1.98	1.98	1.98	1.97	1.97	1.97
	pred	1.99	1.99	1.99	1.99	2	2	2
co 3 eq acac ax ncs	data	1.93	1.93	1.92	1.92	1.92	1.91	1.91
	pred	1.9	1.9	1.9	1.9	1.9	1.9	1.9
co 3 eq ox ax ncs	data	1.94	1.94	1.94	1.93	1.92	1.91	1.91
	pred	1.9	1.9	1.9	1.9	1.91	1.91	1.91
co 3 eq acac ax co	data	1.93	1.94	1.94	1.93	1.92	1.91	1.91
	pred	1.91	1.91	1.91	1.91	1.91	1.91	1.91
ni 2 eq tbuc ax tbuc	data	1.93	1.93	1.92	1.92	1.91	1.91	1.91
	pred	1.91	1.91	1.91	1.92	1.92	1.92	1.93
ni 2 eq bipy ax nh3	data	1.95	1.95	1.95	1.95	1.95	1.96	1.95
	pred	1.94	1.94	1.94	1.94	1.94	1.95	1.96
ni 2 eq en ax en	data	1.98	1.98	1.98	1.98	1.98	1.98	1.98
	pred	1.96	1.97	1.97	1.97	1.98	1.98	1.99
ni 2 eq co ax co	data	1.87	1.88	1.89	1.9	1.91	1.92	1.93
	pred	1.9	1.9	1.9	1.91	1.91	1.92	1.92
ni 2 eq nh3 ax nh3	data	1.95	1.95	1.95	1.95	1.95	1.95	1.95
	pred	1.93	1.94	1.94	1.94	1.95	1.96	1.96
ni 2 eq acac ax cn	data	1.9	1.9	1.9	1.91	1.91	1.91	1.92
	pred	1.89	1.89	1.89	1.89	1.89	1.9	1.9
ni 2 eq pisc ax pisc	data	1.87	1.87	1.88	1.88	1.89	1.89	1.9
	pred	1.86	1.86	1.86	1.87	1.87	1.87	1.87
ni 2 eq en ax h2o	data	1.97	1.97	1.97	1.97	1.96	1.96	1.96
	pred	1.96	1.96	1.96	1.96	1.96	1.96	1.96
ni 2 eq bipy ax bipy	data	1.94	1.95	1.95	1.95	1.95	1.96	1.96
	pred	1.94	1.94	1.95	1.95	1.96	1.96	1.97
ni 2 eq ncs ax ncs	data	1.88	1.88	1.88	1.89	1.89	1.9	1.9
	pred	1.87	1.87	1.87	1.88	1.89	1.89	1.9
ni 2 eq cn ax cn	data	-	-	1.94	1.94	1.95	1.96	1.96
	pred			1.92	1.92	1.93	1.93	1.94
ni 2 eq acac ax co	data	1.89	1.89	1.88	1.88	1.88	1.88	1.88
	pred	1.87	1.87	1.87	1.88	1.88	1.88	1.88
ni 2 eq c2h3ns ax c2h3ns	data	1.86	1.87	1.87	1.88	1.89	1.89	1.9
	pred	1.88	1.88	1.88	1.89	1.89	1.89	1.89
ni 2 eq acac ax nh3	data	1.95	1.94	1.94	1.94	1.94	1.94	1.94
	pred	1.91	1.91	1.91	1.91	1.91	1.91	1.91
ni 2 eq acac ax acac	data	1.89	1.89	1.89	1.89	1.89	1.89	1.9
	pred	1.9	1.9	1.9	1.9	1.9	1.9	1.9
ni 2 eq ox ax ox	data	1.93	1.92	1.92	1.91	1.91	1.91	1.91
	pred	1.91	1.91	1.91	1.91	1.91	1.91	1.92

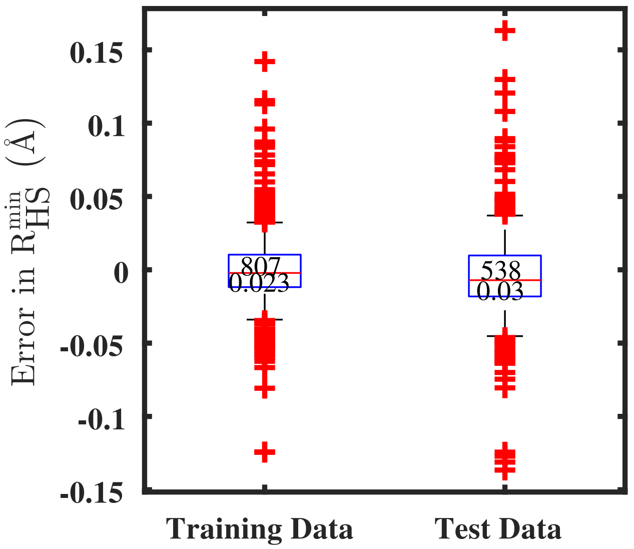

Figure S22: Error boxplot for regression of R_{HS}^{min} using an ANN, showing training and test data comparison. The top number indicates the number of trials, while the bottom indicates the RMSE.

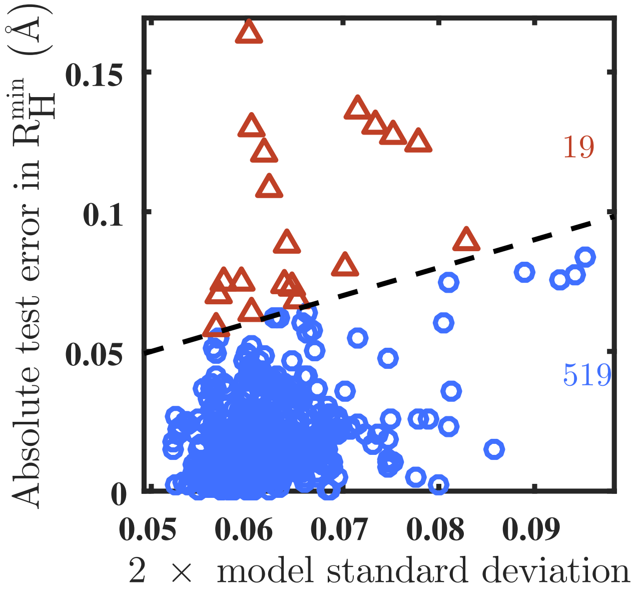

Figure S23: Parity plot for 2 standard deviation from the mean prediction and absolute prediction error for test case R_{HS}^{min} prediction using ANN. All units are Å. The black line is y=x.

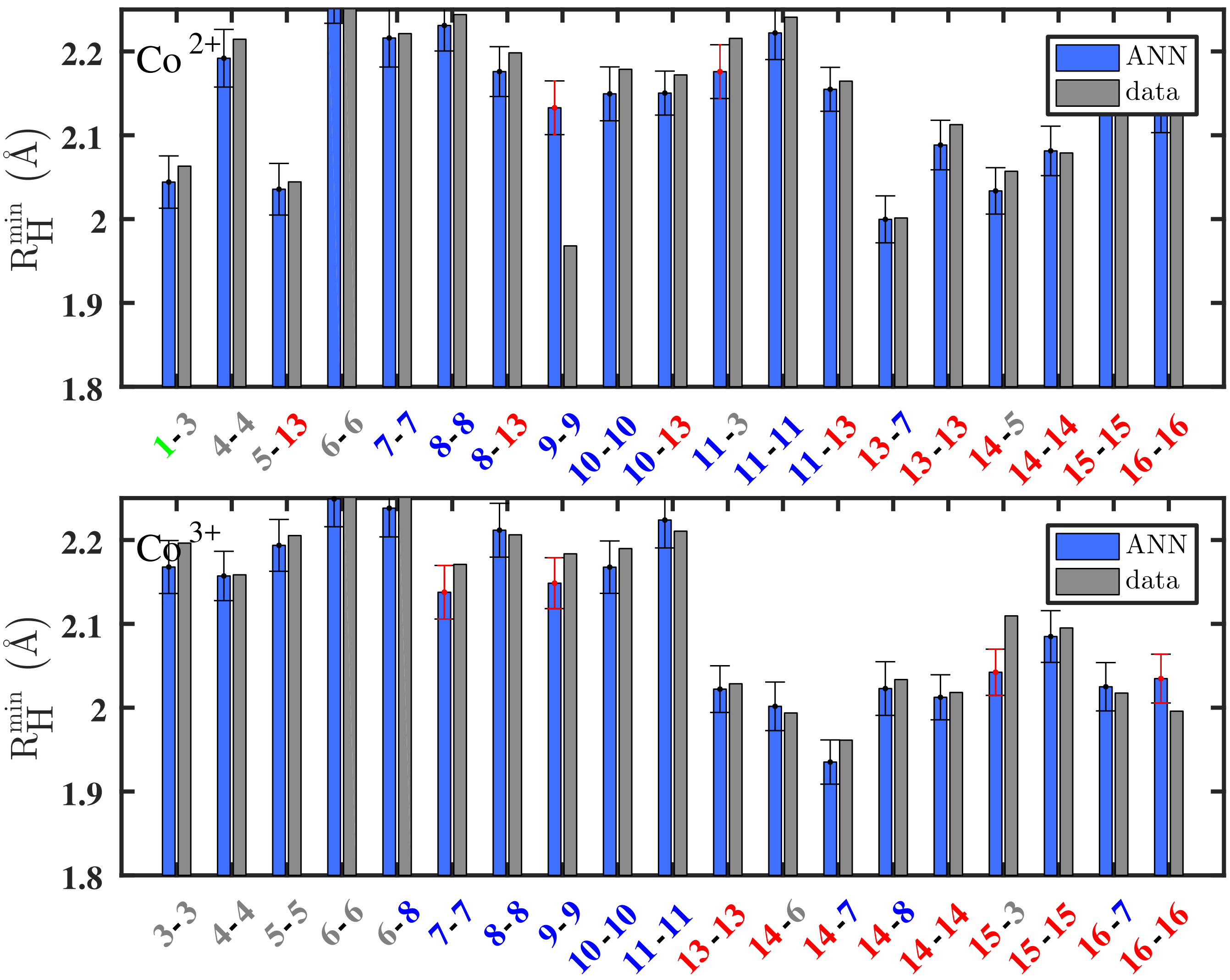

Figure S24: Model predictions of R^{min}_{HS} and data for high-spin Co using an ANN. The ligands are described by two numbers indicating the equatorial first and then the axial, color coded by ligand identity (green for halogen, gray for carbon, blue for nitrogen, and red for oxygen). The error bars represent an estimated 1 standard deviation from the mean prediction.

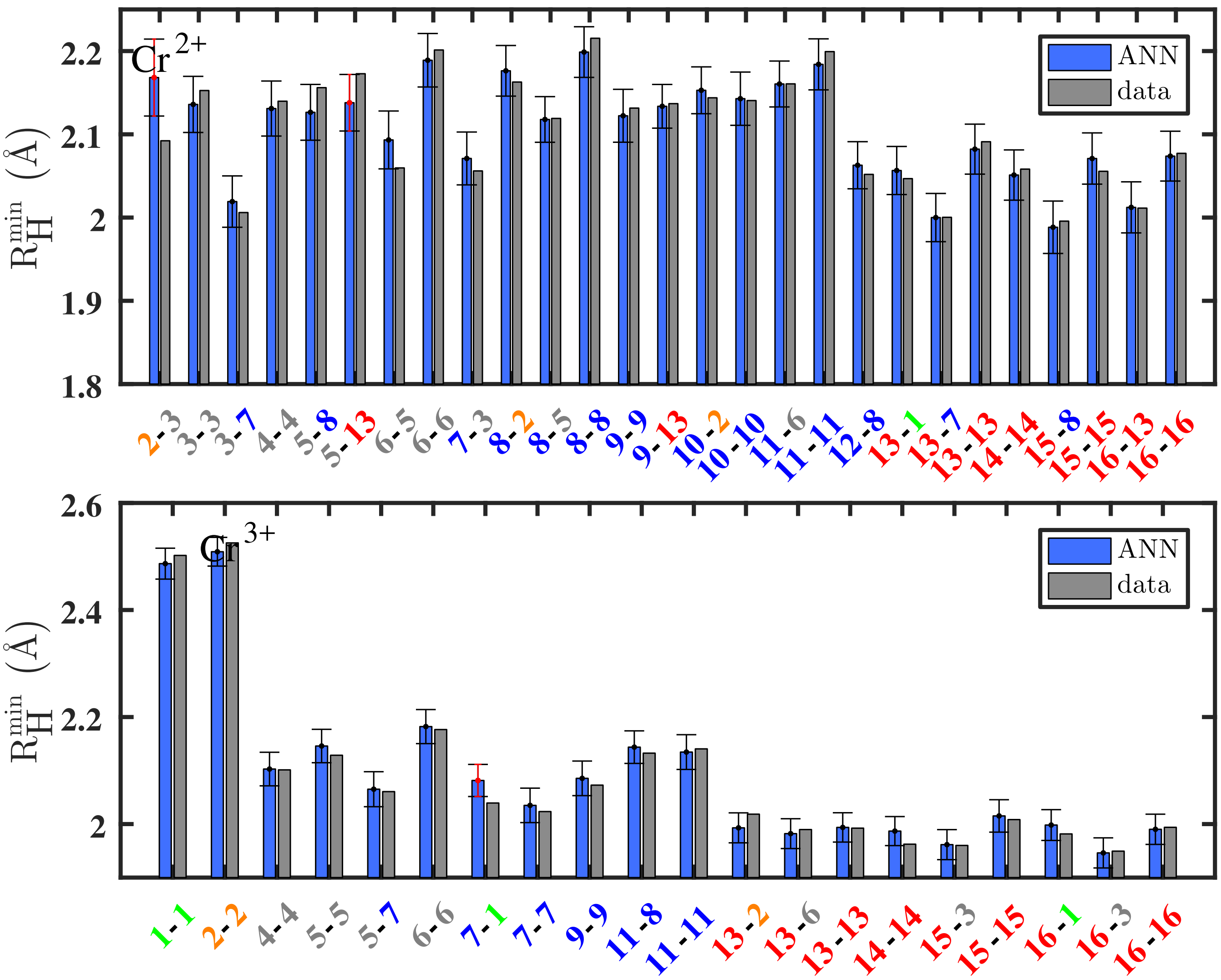

Figure S25: Model predictions of R^{min}_{HS} and data for high-spin Cr using an ANN. The ligands are described by two numbers indicating the equatorial first and then the axial, color coded by ligand identity (green for halogen, gray for carbon, blue for nitrogen, and red for oxygen). The error bars represent an estimated 1 standard deviation from the mean prediction.

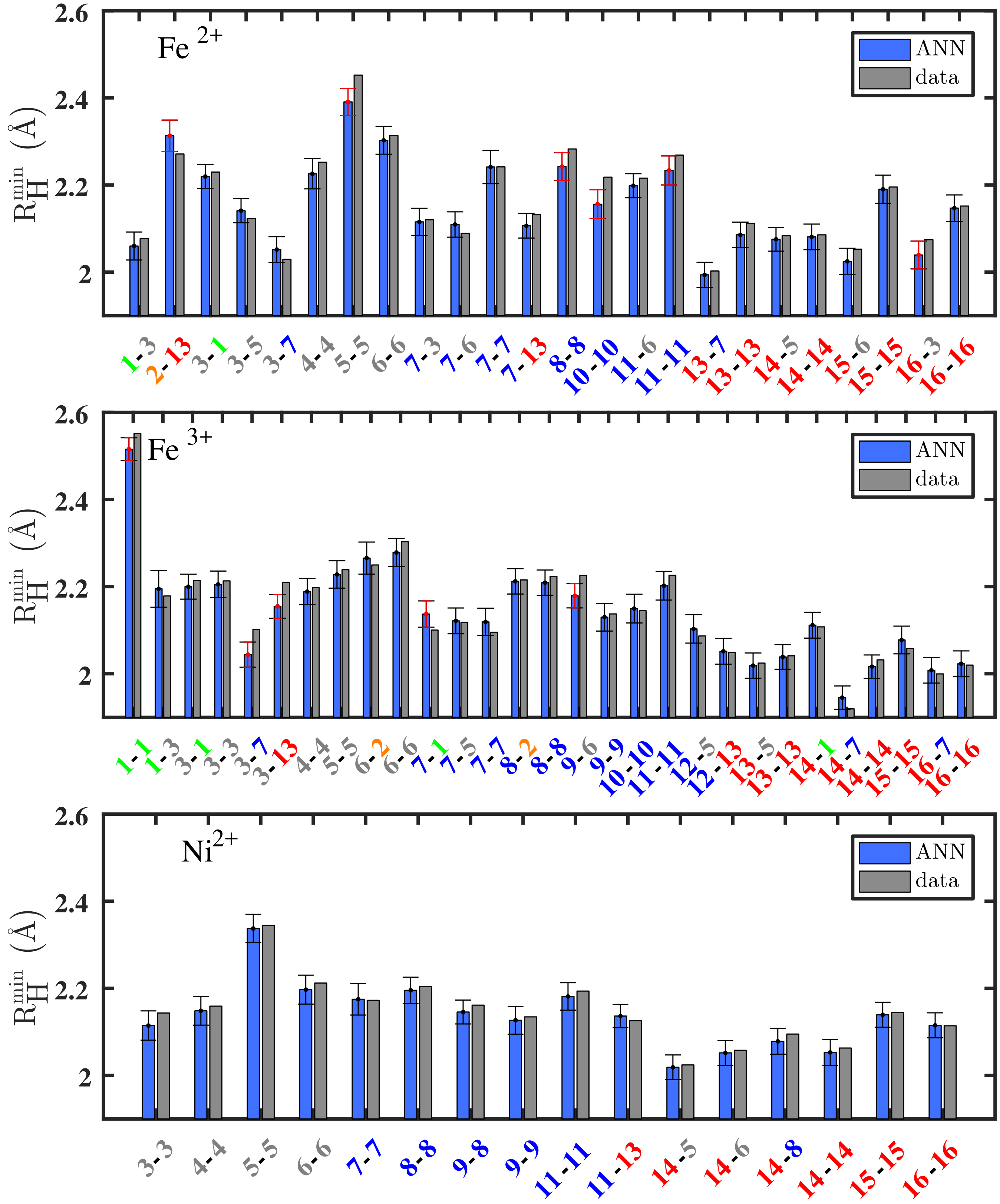

Figure S26: Model predictions of R_{HS}^{min} and data for high-spin Fe(II) (top), Fe(III) (middle) and Ni(II) (bottom) using an ANN. The ligands are described by two numbers indicating the equatorial first and then the axial, color coded by ligand identity (green for halogen, gray for carbon, blue for nitrogen, and red for oxygen). The error bars represent an estimated 1 standard deviation from the mean prediction.

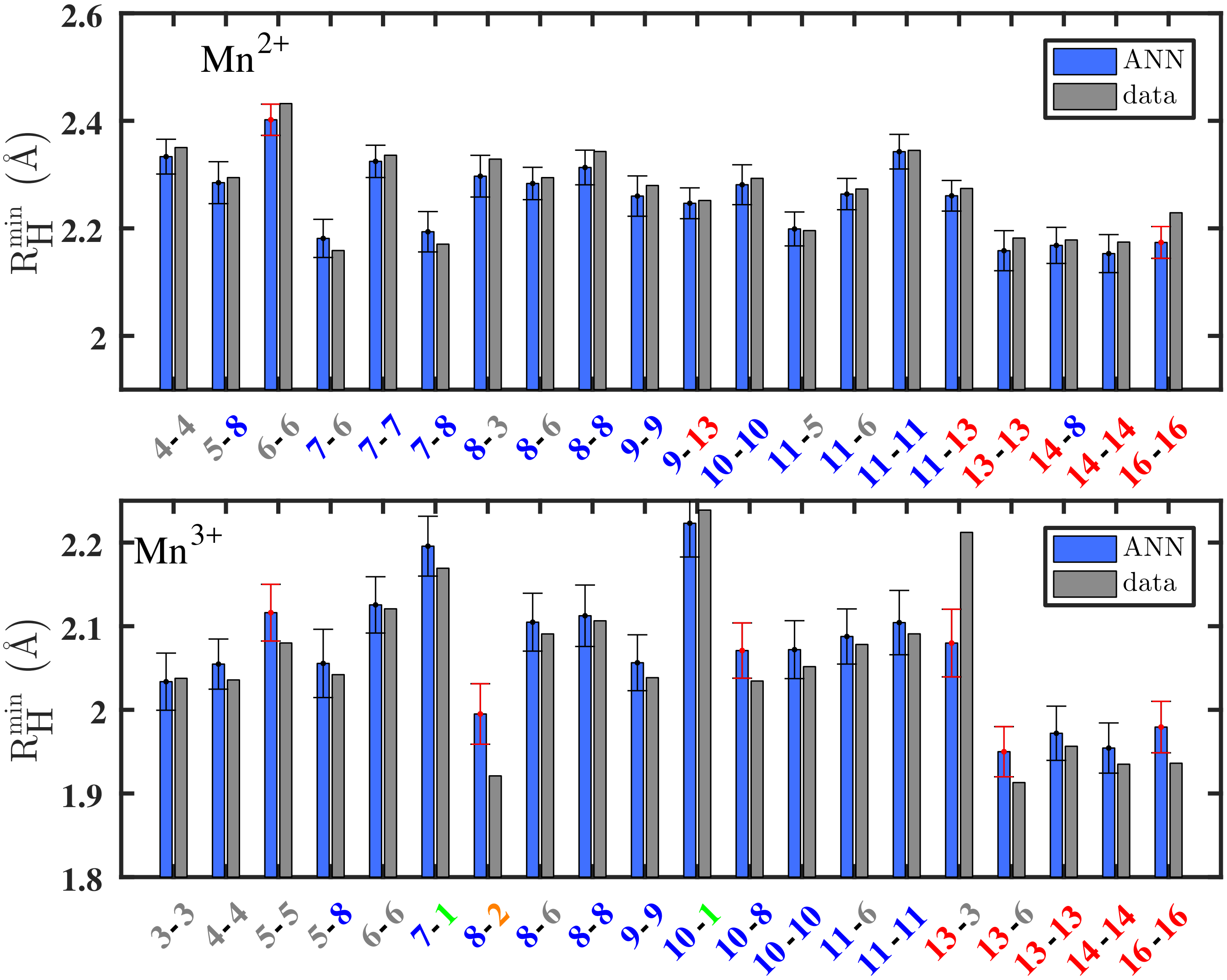

Figure S27: Model predictions of R_{HS}^{min} and data for high-spin Mn using an ANN. The ligands are described by two numbers indicating the equatorial first and then the axial, color coded by ligand identity (green for halogen, gray for carbon, blue for nitrogen, and red for oxygen). The error bars represent an estimated 1 standard deviation from the mean prediction.

Table S28: HS bond distance predictions and data in Å, compared for the full set of complexes. Each complex is named [metal oxidation axial ligand eq equatorial ligand]. For each complex, the first row has the data obtained from DFT using varying fractions of exact exchange (a_{HF}) and the second row has the ANN predictions

name		a_{HF}						
name	type	0	0.05	0.1	0.15	0.2	0.25	0.3
cr 2 eq h2o ax ncs	data	1.98	1.98	1.99	2	2	2	2.01
	pred	1.99	2	2	2	2	2	2
cr 2 eq porphyrin ax nh3	data	2.07	2.07	2.07	2.07	2.05	2.05	2.03
	pred	2.07	2.06	2.06	2.06	2.06	2.06	2.06
cr 2 eq scn ax pisc	data	2.04	2.05	2.07	2.08	2.09	2.1	2.11
	pred	2.12	2.13	2.14	2.15	2.17	2.19	2.21
cr 2 eq ncs ax pisc	data	2.02	2.04	2.04	2.05	2.06	2.06	2.07
	pred	2.06	2.06	2.06	2.07	2.07	2.07	2.08
cr 2 eq cn ax nh3	data	2.12	2.13	2.14	2.15	2.16	2.19	2.2
	pred	2.12	2.12	2.12	2.12	2.13	2.13	2.13
cr 2 eq tbuc ax tbuc	data	2.04	2.05	2.05	2.05	2.06	2.06	2.06
	pred	2.07	2.07	2.07	2.07	2.07	2.07	2.07
cr 2 eq phen ax scn	data	2.13	2.14	2.14	2.14	2.14	2.15	2.15
	pred	2.14	2.14	2.14	2.15	2.15	2.16	2.16
cr 2 eq en ax co	data	2.16	2.16	2.16	2.16	2.16	2.16	2.16
	pred	2.16	2.16	2.16	2.16	2.16	2.16	2.16
cr 2 eq bipy ax bipy	data	2.1	2.12	2.12	2.13	2.13	2.13	2.14
	pred	2.12	2.12	2.12	2.12	2.12	2.12	2.12
cr 2 eq en ax en	data	2.2	2.2	2.2	2.2	2.2	2.2	2.2
	pred	2.19	2.19	2.19	2.19	2.18	2.18	2.18
cr 2 eq co ax cn	data	2.02	2.04	2.05	2.05	2.06	2.06	2.07
	pred	2.07	2.07	2.08	2.08	2.09	2.11	2.12
cr 2 eq pisc ax pisc	data	2.11	2.12	2.13	2.14	2.15	2.16	2.17
	pred	2.11	2.12	2.12	2.13	2.14	2.14	2.15
cr 2 eq ox ax h2o	data	2.01	2.01	2.01	2.01	2.01	2.01	2.01
	pred	2	2	2.01	2.01	2.01	2.02	2.02
cr 2 eq h2o ax cl	data	2.05	2.05	2.05	2.05	2.05	2.05	2.05
	pred	2.06	2.06	2.06	2.06	2.06	2.06	2.06
cr 2 eq nh3 ax scn	data	2.16	2.16	2.16	2.16	2.16	2.16	2.16
	pred	2.17	2.17	2.17	2.18	2.18	2.18	2.18
cr 2 eq ox ax ox	data	2.07	2.07	2.07	2.08	2.08	2.08	2.08
	pred	2.07	2.07	2.07	2.07	2.07	2.08	2.08
cr 2 eq h2o ax h2o	data	2.09	2.09	2.09	2.09	2.09	2.09	2.09
	pred	2.08	2.08	2.08	2.08	2.08	2.08	2.08
cr 2 eq cn ax h2o	data	2.14	2.15	2.16	2.17	2.17	2.18	2.19
	pred	2.12	2.13	2.13	2.13	2.14	2.14	2.14
cr 2 eq pisc ax ncs	data	-	1.99	2	2	2.01	2.01	2.01
	pred		2.01	2.01	2.02	2.02	2.02	2.02
cr 2 eq acac ax acac	data	2.04	2.05	2.05	2.06	2.06	2.06	2.06
	pred	2.05	2.05	2.05	2.05	2.05	2.05	2.05
cr 2 eq bipy ax h2o	data	2.12	2.13	2.13	2.13	2.14	2.14	2.14
	pred	2.14	2.14	2.13	2.13	2.13	2.13	2.13
cr 2 eq nh3 ax nh3	data	2.21	2.21	2.21	2.21	2.22	2.21	2.21
	pred	2.2	2.2	2.2	2.2	2.2	2.2	2.2
cr 2 eq co ax co	data	2.15	2.16	2.19	2.19	2.2	2.21	2.22
	pred	2.16	2.17	2.18	2.18	2.19	2.2	2.2
cr 2 eq c2h3ns ax c2h3ns	data	2.1	2.11	2.13	2.14	2.14	2.15	2.16
	pred	2.11	2.11	2.12	2.12	2.13	2.14	2.14
cr 2 eq phen ax phen	data	2.12	2.13	2.13	2.14	2.14	2.14	2.15
	pred	2.14	2.14	2.14	2.14	2.14	2.14	2.15
cr 2 eq tbuc ax nh3	data	1.99	1.99	2	1.99	2	1.99	2
	pred	1.99	1.99	1.99	1.99	1.99	1.99	1.99
cr 2 eq nh3 ax cn	data	2.09	2.1	2.11	2.11	2.12	2.13	2.13
	pred	2.12	2.12	2.12	2.12	2.12	2.12	2.12
cr 3 eq c2h3ns ax c2h3ns	data	2.09	2.09	2.09	2.1	2.1	2.1	2.11
	pred	2.09	2.09	2.1	2.1	2.1	2.11	2.11
cr 3 eq acac ax acac	data	1.97	1.97	1.97	1.96	1.96	1.96	1.97
	pred	1.99	1.99	1.99	1.99	1.99	1.99	1.98
cr 3 eq h2o ax scn	data	2.04	2.03	2.03	2.02	2.02	2.02	2.01
	pred	2	2	2	2	1.99	1.99	1.99
cr 3 eq en ax nh3	data	2.15	2.15	2.14	2.14	2.13	2.13	2.13
	pred	2.15	2.15	2.15	2.15	2.14	2.14	2.14
cr 3 eq scn ax scn	data	2.53	2.53	2.53	2.52	2.53	2.52	2.52
	pred	2.51	2.51	2.51	2.51	2.51	2.51	2.51
cr 3 eq en ax en	data	2.16	2.15	2.15	2.14	2.14	2.13	2.13
	pred	2.15	2.15	2.14	2.14	2.13	2.13	2.13
cr 3 eq cn ax cn	data	2.12	2.12	2.12	2.13	2.13	2.13	2.13
	pred	2.14	2.14	2.15	2.15	2.15	2.14	2.14
cr 3 eq cn ax ncs	data	2.05	2.05	2.06	2.06	2.06	2.06	2.07
	pred	2.04	2.05	2.05	2.06	2.07	2.07	2.08
cr 3 eq tbuc ax tbuc	data	-	-	2.02	2.01	2.01	2	2
	pred			2.02	2.02	2.02	2.01	2.01
cr 3 eq ncs ax ncs	data	2.02	2.02	2.02	2.02	2.02	2.02	2.02
	pred	2.03	2.03	2.03	2.03	2.03	2.04	2.04
cr 3 eq ox ax ox	data	2.01	2.01	2	2	1.99	1.99	1.99
	pred	2	2	1.99	1.99	1.99	1.99	1.99
cr 3 eq cl ax cl	data	2.52	2.52	2.51	2.51	2.5	2.5	2.49
	pred	2.47	2.48	2.48	2.48	2.49	2.49	2.49

cr 3 eq co ax co	data	2.17	2.17	2.17	2.18	2.18	2.18	2.18
	pred	2.18	2.18	2.18	2.18	2.18	2.18	2.18
cr 3 eq h2o ax co	data	2	2	2	1.99	1.99	1.99	1.99
	pred	2	1.99	1.99	1.98	1.98	1.98	1.98
cr 3 eq bipy ax bipy	data	2.08	2.08	2.08	2.08	2.07	2.07	2.07
	pred	2.09	2.09	2.09	2.09	2.09	2.08	2.08
cr 3 eq ncs ax cl	data	2.03	2.03	2.04	2.04	2.04	2.04	2.04
	pred	2.06	2.06	2.07	2.07	2.08	2.09	2.1
cr 3 eq tbuc ax pisc	data	1.98	1.98	1.97	1.97	1.96	1.95	1.95
	pred	1.97	1.96	1.96	1.96	1.96	1.96	1.96
cr 3 eq ox ax cl	data	2	1.99	1.99	1.99	1.98	1.98	1.97
	pred	1.99	1.99	2	2	2	2	2
cr 3 eq h2o ax h2o	data	2	2	2	2	1.99	1.99	1.99
	pred	2	2	2	2	1.99	1.99	1.99
cr 3 eq ox ax pisc	data	1.97	1.96	1.96	1.95	1.95	1.95	1.94
	pred	1.95	1.95	1.95	1.95	1.95	1.95	1.94
mn 2 eq acac ax nh3	data	2.17	2.17	2.17	2.17	2.18	2.18	2.18
	pred	2.16	2.16	2.17	2.17	2.17	2.17	2.17
mn 2 eq nh3 ax nh3	data	2.34	2.34	2.35	2.34	2.34	2.34	2.34
	pred	2.32	2.32	2.32	2.31	2.31	2.31	2.31
mn 2 eq co ax co	data	2.41	2.42	2.43	2.43	2.43	2.43	2.43
	pred	2.38	2.39	2.39	2.4	2.4	2.41	2.41
mn 2 eq cn ax nh3	data	2.17	2.27	2.28	2.29	2.29	2.3	2.31
	pred	2.26	2.26	2.27	2.28	2.28	2.29	2.3
mn 2 eq en ax en	data	2.35	2.35	2.35	2.35	2.35	2.34	2.34
	pred	2.35	2.35	2.35	2.35	2.34	2.34	2.33
mn 2 eq acac ax acac	data	2.18	2.18	2.18	2.18	2.17	2.17	2.17
	pred	2.16	2.16	2.16	2.15	2.15	2.15	2.15
mn 2 eq en ax cn	data	2.16	2.17	2.18	2.19	2.2	2.2	2.2
	pred	2.19	2.19	2.2	2.2	2.2	2.2	2.2
mn 2 eq bipy ax h2o	data	2.25	2.25	2.25	2.25	2.25	2.25	2.25
	pred	2.24	2.24	2.24	2.25	2.25	2.25	2.25
mn 2 eq h2o ax h2o	data	2.18	2.18	2.18	2.18	2.18	2.18	2.18
	pred	2.16	2.16	2.16	2.16	2.16	2.16	2.16
mn 2 eq ncs ax nh3	data	2.16	2.17	2.18	2.17	2.17	2.18	2.18
	pred	2.19	2.19	2.19	2.19	2.19	2.2	2.2
mn 2 eq bipy ax bipy	data	2.28	2.28	2.28	2.28	2.28	2.28	2.28
	pred	2.27	2.27	2.27	2.26	2.26	2.26	2.25
mn 2 eq ox ax ox	data	2.23	2.23	2.23	2.23	2.23	-	-
	pred	2.19	2.19	2.18	2.18	2.17		
mn 2 eq c2h3ns ax c2h3ns	data	2.33	2.34	2.34	2.35	2.35	2.35	2.36
	pred	2.32	2.33	2.33	2.33	2.33	2.34	2.34
mn 2 eq ncs ax co	data	2.15	2.16	2.16	2.16	2.16	2.16	2.16
	pred	2.17	2.18	2.18	2.18	2.18	2.18	2.19
mn 2 eq ncs ax ncs	data	2.33	2.34	2.33	2.33	2.34	2.33	2.33
	pred	2.31	2.32	2.32	2.32	2.32	2.32	2.32
mn 2 eq phen ax phen	data	2.3	2.3	2.3	2.29	2.29	2.29	2.29
	pred	2.29	2.29	2.29	2.28	2.28	2.28	2.28
mn 2 eq nh3 ax co	data	2.3	2.3	2.3	2.3	2.29	2.29	2.29
	pred	2.28	2.28	2.28	2.28	2.28	2.28	2.28
mn 2 eq en ax h2o	data	2.27	2.28	2.27	2.27	2.27	2.27	2.27
	pred	2.26	2.26	2.26	2.26	2.26	2.26	2.26
mn 2 eq en ax co	data	2.28	2.28	2.28	2.27	2.27	2.27	2.27
	pred	2.26	2.26	2.26	2.26	2.26	2.26	2.26
mn 2 eq nh3 ax pisc	data	2.29	2.3	2.31	2.32	2.33	2.34	2.34
	pred	2.27	2.28	2.29	2.29	2.3	2.3	2.31
mn 3 eq en ax co	data	2.09	2.09	2.08	2.08	2.08	2.08	2.07
	pred	2.09	2.09	2.09	2.09	2.09	2.09	2.09
mn 3 eq phen ax cl	data	2.12	2.12	2.24	2.24	2.24	2.24	2.24
	pred	2.17	2.18	2.2	2.21	2.22	2.24	2.25
mn 3 eq phen ax phen	data	2.06	2.06	2.05	2.05	2.05	2.05	2.04
	pred	2.07	2.07	2.07	2.07	2.07	2.07	2.07
mn 3 eq cn ax nh3	data	2.02	2.04	2.04	2.04	2.04	2.04	2.04
	pred	2.05	2.05	2.05	2.05	2.06	2.06	2.06
mn 3 eq pisc ax pisc	data	2.01	2.02	2.02	2.03	2.04	2.04	2.04
	pred	2.02	2.03	2.03	2.03	2.03	2.04	2.04
mn 3 eq ncs ax cl	data	2.17	2.17	2.17	2.17	2.17	2.17	2.16
	pred	2.17	2.18	2.18	2.19	2.2	2.2	2.21
mn 3 eq h2o ax co	data	1.93	1.92	1.92	1.92	1.91	1.91	1.91
	pred	1.96	1.96	1.95	1.95	1.95	1.95	1.95
mn 3 eq ox ax ox	data	1.95	1.95	1.94	1.94	1.94	1.93	1.93
	pred	1.99	1.98	1.98	1.98	1.98	1.98	1.98
mn 3 eq co ax co	data	2.12	2.12	2.12	2.12	2.12	2.12	2.12
	pred	2.13	2.13	2.12	2.12	2.13	2.13	2.13
mn 3 eq acac ax acac	data	1.95	1.94	1.94	1.94	1.93	1.93	1.93
	pred	1.97	1.96	1.96	1.96	1.95	1.95	1.95
mn 3 eq nh3 ax nh3	data	2.12	2.12	2.12	2.11	2.11	2.1	2.1
	pred	2.12	2.12	2.12	2.11	2.11	2.11	2.11
mn 3 eq nh3 ax co	data	2.11	2.11	2.1	2.1	2.09	2.09	2.09
	pred	2.12	2.11	2.11	2.11	2.1	2.1	2.1
mn 3 eq cn ax cn	data	2.07	2.08	2.08	2.08	2.08	2.05	2.05
	pred	2.11	2.11	2.11	2.11	2.12	2.12	2.13
mn 3 eq nh3 ax scn	data	1.89	1.89	1.91	1.92	1.92	1.93	1.94
	pred	2	2	2	1.99	2	2	2
mn 3 eq bipy ax bipy	data	2.04	2.04	2.04	2.04	2.04	2.04	2.04
	pred	2.06	2.06	2.06	2.06	2.06	2.06	2.06
mn 3 eq h2o ax pisc	data	2.23	2.23	2.22	2.22	2.21	2.21	-
	pred	2.11	2.1	2.1	2.09	2.08	2.07	
mn 3 eq en ax en	data	2.1	2.1	2.1	2.09	2.09	2.09	2.08
	pred	2.12	2.11	2.11	2.11	2.1	2.1	2.1

mn 3 eq phen ax nh3	data	2.04	2.03	2.04	2.03	2.03	2.03	2.03
	pred	2.07	2.07	2.07	2.07	2.07	2.07	2.07
mn 3 eq h2o ax h2o	data	1.97	1.97	1.96	1.96	1.96	1.95	1.95
	pred	1.98	1.98	1.98	1.97	1.97	1.97	1.97
mn 3 eq c2h3ns ax c2h3ns	data	2.03	2.03	2.03	2.04	2.04	2.04	2.04
	pred	2.04	2.05	2.05	2.05	2.05	2.06	2.06
fe 2 eq co ax co	data	2.27	2.29	2.3	2.31	2.31	2.33	2.33
	pred	2.28	2.28	2.29	2.3	2.3	2.31	2.31
fe 2 eq acac ax acac	data	2.07	2.08	2.08	2.08	2.09	2.09	2.11
	pred	2.07	2.07	2.08	2.08	2.08	2.08	2.09
fe 2 eq tbuc ax co	data	1.91	1.94	1.97	2.05	2.05	2.05	2.06
	pred	1.98	1.99	2	2.01	2.02	2.04	2.06
fe 2 eq cn ax cn	data	2.44	2.45	2.45	2.45	2.45	2.45	2.46
	pred	2.36	2.37	2.38	2.39	2.39	2.39	2.4
fe 2 eq tbuc ax tbuc	data	2.2	2.2	2.2	2.2	2.2	2.19	-
	pred	2.19	2.19	2.19	2.19	2.19	2.19	
fe 2 eq h2o ax ncs	data	1.96	1.97	1.98	1.99	2	2.01	2.01
	pred	1.99	1.99	1.99	1.99	1.99	2	2
fe 2 eq en ax en	data	2.27	2.27	2.27	2.27	2.27	2.27	2.27
	pred	2.25	2.25	2.24	2.24	2.23	2.23	2.22
fe 2 eq cl ax pisc	data	2.01	2.02	2.04	2.05	2.08	2.13	2.15
	pred	2.03	2.04	2.04	2.05	2.06	2.07	2.09
fe 2 eq ncs ax h2o	data	2.04	2.05	2.05	2.13	2.13	2.13	2.14
	pred	2.09	2.09	2.09	2.1	2.11	2.11	2.12
fe 2 eq pisc ax cl	data	2.17	2.18	2.19	2.21	2.23	2.24	2.25
	pred	2.19	2.2	2.21	2.21	2.22	2.23	2.24
fe 2 eq phen ax phen	data	2.2	2.2	2.2	2.2	2.22	2.02	2.03
	pred	2.19	2.18	2.17	2.17	2.16	2.15	2.14
fe 2 eq scn ax h2o	data	2.33	2.31	2.3	2.28	2.27	2.29	2.28
	pred	2.31	2.31	2.31	2.31	2.31	2.31	2.32
fe 2 eq nh3 ax nh3	data	2.29	2.29	2.28	2.28	2.28	2.28	2.28
	pred	2.25	2.25	2.25	2.24	2.24	2.24	2.24
fe 2 eq c2h3ns ax c2h3ns	data	2.22	2.22	2.23	2.25	2.25	2.26	2.26
	pred	2.21	2.21	2.22	2.22	2.23	2.23	2.23
fe 2 eq ox ax pisc	data	2.08	2.07	2.07	2.07	2.07	2.08	2.08
	pred	2.02	2.02	2.03	2.03	2.04	2.05	2.06
fe 2 eq pisc ax cn	data	2.14	2.14	2.14	2.14	2.12	2.14	2.14
	pred	2.13	2.13	2.13	2.14	2.14	2.15	2.15
fe 2 eq acac ax cn	data	2.06	2.07	2.08	2.09	2.08	2.1	2.1
	pred	2.06	2.06	2.07	2.07	2.08	2.08	2.08
fe 2 eq ox ax ox	data	2.16	2.16	2.16	2.15	2.15	2.15	2.15
	pred	2.15	2.15	2.15	2.15	2.15	2.15	2.15
fe 2 eq h2o ax h2o	data	2.11	2.11	2.11	2.11	2.11	2.11	2.11
	pred	2.08	2.08	2.08	2.08	2.09	2.09	2.09
fe 2 eq ncs ax pisc	data	2.12	2.1	2.1	2.14	2.12	-	2.12
	pred	2.11	2.11	2.11	2.11	2.12		2.12
fe 2 eq en ax co	data	2.24	2.23	2.22	2.22	2.22	2.21	2.21
	pred	2.2	2.2	2.2	2.2	2.2	2.2	2.2
fe 2 eq pisc ax ncs	data	2.05	2.04	2.04	2.04	2.03	2.03	2.03
	pred	2.07	2.06	2.06	2.05	2.05	2.05	2.05
fe 2 eq ncs ax co	data	2.13	2.12	2.11	2.1	2.09	2.09	2.09
	pred	2.11	2.11	2.11	2.11	2.11	2.11	2.11
fe 2 eq ncs ax ncs	data	2.23	2.24	2.24	2.24	2.24	2.24	2.25
	pred	2.2	2.21	2.22	2.23	2.24	2.25	2.26
fe 3 eq tbuc ax tbuc	data	2.09	2.08	2.07	2.06	2.06	2.05	2.05
	pred	2.09	2.09	2.08	2.08	2.08	2.07	2.07
fe 3 eq cn ax cn	data	2.25	2.25	2.25	2.24	2.24	2.23	2.23
	pred	2.24	2.24	2.24	2.23	2.23	2.22	2.22
fe 3 eq acac ax acac	data	2.06	2.05	2.05	2.04	2.03	2.02	2.02
	pred	2.04	2.03	2.02	2.02	2.02	2.01	2.01
fe 3 eq porphyrin ax h2o	data	2.05	2.05	2.05	2.05	2.05	2.04	2.03
	pred	2.06	2.06	2.06	2.05	2.05	2.05	2.05
fe 3 eq h2o ax cn	data	2.02	2.02	2.02	2.02	2.02	2.02	2.02
	pred	2.02	2.02	2.02	2.02	2.02	2.02	2.02
fe 3 eq porphyrin ax cn	data	2.09	2.09	2.09	2.09	2.09	2.08	2.08
	pred	2.1	2.1	2.1	2.1	2.1	2.1	2.1
fe 3 eq co ax co	data	2.32	2.31	2.31	2.3	2.3	2.29	2.29
	pred	2.28	2.28	2.28	2.28	2.28	2.28	2.27
fe 3 eq ox ax ox	data	2.04	2.04	2.03	2.03	2.02	2.02	2.01
	pred	2.04	2.03	2.03	2.03	2.02	2.02	2.02
fe 3 eq pisc ax h2o	data	2.16	2.16	2.19	2.18	2.21	2.21	2.2
	pred	2.15	2.15	2.15	2.16	2.15	2.15	2.15
fe 3 eq acac ax cl	data	2.14	2.13	2.12	2.11	2.11	2.11	2.1
	pred	2.11	2.11	2.11	2.11	2.11	2.11	2.11
fe 3 eq ox ax ncs	data	2.03	2.02	2.01	2.01	2	1.99	1.99
	pred	2.02	2.02	2.01	2.01	2.01	2	2
fe 3 eq en ax en	data	2.26	2.25	2.24	2.23	2.23	2.22	2.21
	pred	2.22	2.22	2.21	2.21	2.2	2.2	2.19
fe 3 eq pisc ax pisc	data	2.17	2.19	2.19	2.2	2.21	2.25	2.23
	pred	2.18	2.19	2.2	2.2	2.21	2.21	2.21
fe 3 eq bipy ax co	data	2.15	2.15	2.17	2.23	2.23	2.22	2.22
	pred	2.18	2.18	2.18	2.18	2.18	2.18	2.18
fe 3 eq pisc ax ncs	data	2	1.97	1.96	1.95	2.1	2.1	2.11
	pred	2.05	2.05	2.04	2.04	2.04	2.04	2.05
fe 3 eq ncs ax cl	data	2.12	2.11	2.11	2.1	2.1	2.1	2.09
	pred	2.14	2.14	2.14	2.14	2.14	2.14	2.14
fe 3 eq phen ax phen	data	2.17	2.16	2.15	2.15	2.14	2.14	2.13
	pred	2.15	2.15	2.15	2.15	2.15	2.15	2.15
fe 3 eq h2o ax h2o	data	2.07	2.06	2.05	2.05	2.04	2.04	2.03
	pred	2.05	2.05	2.04	2.04	2.04	2.04	2.03

fe 3 eq cl ax cl	data	2.58	2.57	2.57	2.56	2.55	2.54	2.54
	pred	2.51	2.51	2.51	2.52	2.52	2.52	2.52
fe 3 eq pisc ax cl	data	2.18	2.19	2.2	2.21	2.21	2.21	2.21
	pred	2.19	2.2	2.2	2.2	2.2	2.2	2.2
fe 3 eq c2h3ns ax c2h3ns	data	2.2	2.2	2.2	2.2	2.2	2.19	2.2
	pred	2.19	2.19	2.19	2.19	2.19	2.19	2.19
fe 3 eq nh3 ax scn	data	2.23	2.23	2.23	2.22	2.22	2.2	2.19
	pred	2.24	2.23	2.22	2.22	2.21	2.21	2.2
fe 3 eq acac ax ncs	data	1.92	1.93	1.92	1.92	1.92	1.93	1.93
	pred	1.93	1.93	1.94	1.94	1.95	1.95	1.95
fe 3 eq co ax scn	data	-	-	2.21	2.23	2.25	2.27	2.29
	pred			2.25	2.26	2.27	2.27	2.28
fe 3 eq bipy ax bipy	data	2.14	2.14	2.14	2.14	2.14	2.13	2.13
	pred	2.14	2.14	2.13	2.13	2.13	2.13	2.13
fe 3 eq ncs ax cn	data	2.14	2.14	2.13	2.12	2.12	2.11	2.11
	pred	2.12	2.12	2.12	2.12	2.12	2.12	2.12
fe 3 eq ncs ax ncs	data	2.12	2.12	2.11	2.1	2.1	2.09	2.08
	pred	2.12	2.12	2.12	2.12	2.12	2.12	2.12
fe 3 eq nh3 ax nh3	data	2.26	2.25	2.24	2.23	2.22	2.22	2.21
	pred	2.22	2.22	2.21	2.21	2.21	2.21	2.2
fe 3 eq cl ax pisc	data	2.16	2.17	2.18	2.19	2.18	2.19	2.19
	pred	2.14	2.15	2.16	2.18	2.2	2.21	2.23
co 2 eq ox ax ox	data	2.15	2.12	2.16	2.15	2.15	2.15	2.15
	pred	2.13	2.13	2.13	2.13	2.13	2.13	2.13
co 2 eq en ax en	data	2.25	2.25	2.24	2.24	2.24	2.24	2.24
	pred	2.23	2.23	2.23	2.22	2.22	2.22	2.22
co 2 eq tbuc ax tbuc	data	2.17	2.18	2.18	2.17	2.17	2.17	2.17
	pred	2.17	2.17	2.17	2.17	2.16	2.16	2.16
co 2 eq cl ax pisc	data	1.94	1.97	2	2.03	2.06	2.09	2.11
	pred	2.02	2.03	2.03	2.04	2.04	2.06	2.07
co 2 eq cn ax h2o	data	1.99	2.01	2.02	2.03	2.04	2.05	2.06
	pred	2.02	2.02	2.02	2.03	2.04	2.04	2.05
co 2 eq en ax pisc	data	2.11	2.16	2.19	2.21	2.22	2.21	2.21
	pred	2.15	2.16	2.17	2.17	2.18	2.18	2.18
co 2 eq phen ax h2o	data	2.16	2.16	2.16	2.15	2.17	2.17	2.15
	pred	2.15	2.15	2.15	2.15	2.15	2.15	2.15
co 2 eq phen ax phen	data	2.15	2.16	2.16	2.18	2.18	2.18	2.18
	pred	2.17	2.16	2.16	2.15	2.15	2.15	2.14
co 2 eq h2o ax ncs	data	1.97	1.98	1.98	1.99	2	2.01	2.01
	pred	2	2	2	2	2	2	2.01
co 2 eq c2h3ns ax c2h3ns	data	2.18	2.19	2.2	2.2	2.21	2.22	2.22
	pred	2.18	2.18	2.18	2.19	2.19	2.2	2.2
co 2 eq nh3 ax nh3	data	2.25	2.25	2.25	2.24	2.24	2.24	2.24
	pred	2.24	2.24	2.23	2.23	2.23	2.23	2.23
co 2 eq acac ax cn	data	2.03	2.03	2.04	2.05	2.06	2.06	2.06
	pred	2.02	2.02	2.03	2.03	2.03	2.04	2.04
co 2 eq nh3 ax h2o	data	2.2	2.2	2.2	2.2	2.2	2.2	2.2
	pred	2.18	2.18	2.18	2.18	2.18	2.17	2.17
co 2 eq acac ax acac	data	2.07	2.08	2.08	2.08	2.08	2.1	2.1
	pred	2.08	2.08	2.08	2.08	2.08	2.08	2.09
co 2 eq ncs ax ncs	data	2.22	2.22	2.22	2.22	2.22	2.22	2.23
	pred	2.19	2.2	2.21	2.21	2.22	2.22	2.22
co 2 eq bipy ax bipy	data	2.18	2.18	2.18	2.17	1.97	-	-
	pred	2.15	2.15	2.14	2.14	2.13		
co 2 eq co ax co	data	2.23	2.25	2.26	2.27	2.27	2.28	2.29
	pred	2.24	2.24	2.25	2.26	2.26	2.27	2.28
co 2 eq en ax h2o	data	2.17	2.17	2.17	2.17	2.16	2.16	2.15
	pred	2.16	2.16	2.16	2.16	2.15	2.15	2.16
co 2 eq h2o ax h2o	data	2.13	2.13	2.12	2.12	2.11	2.11	2.11
	pred	2.09	2.09	2.09	2.09	2.09	2.09	2.09
co 3 eq co ax nh3	data	-	2.26	2.26	2.26	2.25	2.25	2.24
	pred		2.23	2.24	2.24	2.24	2.24	2.24
co 3 eq acac ax nh3	data	2.06	2.06	2.05	2.04	2.03	2.02	2.01
	pred	2.03	2.03	2.03	2.03	2.02	2.02	2.02
co 3 eq tbuc ax pisc	data	1.98	2	2.04	2.08	2.11	2.1	2.05
	pred	2.01	2.02	2.02	2.03	2.04	2.05	2.05
co 3 eq nh3 ax nh3	data	2.24	2.23	2.23	2.22	2.21	2.2	2.19
	pred	2.22	2.22	2.22	2.21	2.21	2.21	2.21
co 3 eq ox ax ox	data	2.04	2.03	2.02	2.02	2	2	2
	pred	2.05	2.04	2.04	2.04	2.03	2.03	2.03
co 3 eq en ax en	data	2.25	2.24	2.23	2.22	2.21	2.2	2.19
	pred	2.23	2.23	2.23	2.23	2.22	2.22	2.22
co 3 eq acac ax acac	data	2.05	2.04	2.04	2.03	2.02	2.01	2
	pred	2.03	2.02	2.02	2.02	2.01	2.01	2.01
co 3 eq phen ax phen	data	2.16	2.16	2.18	2.19	2.19	2.19	2.19
	pred	2.16	2.16	2.16	2.17	2.17	2.17	2.17
co 3 eq cn ax cn	data	2.22	2.22	2.21	2.21	2.21	2.19	2.2
	pred	2.2	2.2	2.2	2.2	2.19	2.19	2.19
co 3 eq c2h3ns ax c2h3ns	data	2.15	2.16	2.16	2.16	2.16	2.16	2.16
	pred	2.16	2.16	2.16	2.16	2.16	2.16	2.16
co 3 eq co ax co	data	2.29	2.28	2.28	2.27	2.26	2.26	2.25
	pred	2.25	2.25	2.25	2.25	2.25	2.25	2.24
co 3 eq tbuc ax tbuc	data	2.09	2.09	2.08	2.07	2.1	2.11	2.11
	pred	2.09	2.09	2.09	2.09	2.08	2.08	2.08
co 3 eq h2o ax h2o	data	2.07	2.06	2.05	2.04	2.03	2.02	2.01
	pred	2.03	2.03	2.03	2.02	2.02	2.02	2.02
co 3 eq ncs ax ncs	data	2.11	2.11	2.11	2.12	2.17	2.17	2.17
	pred	2.13	2.13	2.13	2.14	2.14	2.14	2.14
co 3 eq pisc ax pisc	data	2.13	2.15	2.17	2.19	2.2	2.2	2.21
	pred	2.14	2.15	2.15	2.16	2.17	2.17	2.18

co 3 eq bipy ax bipy	data	2.14	2.14	2.18	2.19	2.18	2.18	2.18
	pred	2.15	2.15	2.15	2.15	2.15	2.15	2.15
co 3 eq acac ax ncs	data	1.89	1.9	1.9	1.93	1.96	1.98	2
	pred	1.92	1.92	1.93	1.93	1.94	1.94	1.95
co 3 eq ox ax ncs	data	2.05	2.04	2.02	2.02	2.02	2.02	2.02
	pred	2.04	2.04	2.04	2.03	2.02	2.02	2.01
co 3 eq acac ax co	data	2.02	2.01	2	2	1.99	1.99	1.99
	pred	2.02	2.01	2.01	2.01	2	2	2
ni 2 eq tbuc ax tbuc	data	2.16	2.16	2.15	2.14	2.14	2.14	2.14
	pred	2.15	2.15	2.14	2.14	2.14	2.14	2.13
ni 2 eq bipy ax nh3	data	2.16	2.16	2.16	2.16	2.16	2.16	2.16
	pred	2.15	2.15	2.15	2.15	2.15	2.14	2.14
ni 2 eq en ax en	data	2.21	2.2	2.2	2.2	2.19	2.19	2.19
	pred	2.2	2.19	2.19	2.19	2.18	2.18	2.17
ni 2 eq co ax co	data	2.17	2.19	2.19	2.2	2.21	2.21	2.22
	pred	2.17	2.17	2.18	2.19	2.2	2.21	2.22
ni 2 eq nh3 ax nh3	data	2.21	2.21	2.21	2.21	2.2	2.2	2.2
	pred	2.2	2.2	2.2	2.2	2.2	2.19	2.19
ni 2 eq acac ax cn	data	2.01	2.01	2.02	2.02	2.02	2.03	2.03
	pred	2.01	2.01	2.01	2.02	2.02	2.02	2.02
ni 2 eq pisc ax pisc	data	2.11	2.12	2.13	2.14	2.14	2.15	2.16
	pred	2.09	2.09	2.1	2.11	2.11	2.12	2.13
ni 2 eq en ax h2o	data	2.13	2.13	2.13	2.13	2.13	2.12	2.12
	pred	2.14	2.14	2.14	2.14	2.14	2.14	2.14
ni 2 eq bipy ax bipy	data	2.13	2.13	2.13	2.13	2.13	2.13	2.13
	pred	2.13	2.13	2.13	2.13	2.13	2.13	2.12
ni 2 eq ncs ax ncs	data	2.17	2.17	2.17	2.17	2.17	2.17	2.18
	pred	2.17	2.17	2.17	2.17	2.17	2.17	2.17
ni 2 eq cn ax cn	data	-	-	2.34	2.34	2.34	2.35	2.35
	pred			2.33	2.33	2.34	2.34	2.34
ni 2 eq acac ax co	data	2.08	2.09	2.08	2.07	2.06	2.05	2.05
	pred	2.05	2.05	2.05	2.05	2.05	2.05	2.06
ni 2 eq c2h3ns ax c2h3ns	data	2.13	2.13	2.14	2.15	2.16	2.16	2.17
	pred	2.13	2.14	2.14	2.14	2.15	2.15	2.16
ni 2 eq acac ax nh3	data	2.1	2.1	2.1	2.1	2.1	2.09	2.09
	pred	2.08	2.08	2.08	2.08	2.08	2.08	2.08
ni 2 eq acac ax acac	data	2.05	2.05	2.07	2.07	2.06	2.06	2.06
	pred	2.06	2.06	2.06	2.05	2.05	2.05	2.05
ni 2 eq ox ax ox	data	2.13	2.13	2.12	2.12	2.11	2.11	2.11
	pred	2.12	2.12	2.12	2.12	2.12	2.11	2.11

Table S29: RMSEs and MUEs (in Å) for minimum metal-ligand bond length prediction on test data by metal and oxidation state for both min(RLS) and min(RHS). The number of test cases is indicated in parentheses.

Species	RMSE (Å)		MUE (Å)	
	min(RLS)	min(RHS)	min(RLS)	min(RHS)
Cr(II)	0.02 (68)	0.02 (78)	0.02	0.01
Cr(III)	0.02 (54)	0.02 (54)	0.01	0.01
Mn(II)	0.02 (65)	0.03 (49)	0.02	0.02
Mn(III)	0.02 (60)	0.05 (59)	0.01	0.03
Fe(II)	0.02 (64)	0.03 (59)	0.01	0.03
Fe(III)	0.02 (84)	0.03 (88)	0.01	0.02
Co(II)	0.03 (55)	0.04 (53)	0.02	0.03
Co(III)	0.02 (52)	0.02 (51)	0.02	0.02
Ni(II)	0.01 (36)	0.02 (47)	0.01	0.01

Table S30: molSimplify initial structure projected (g) gradients and RMS gradients with and without preliminary ANN assisted bond lengths

name		g (kcal/Å)	RMS grad. (Hartree/Bohr)
$\mathrm{Co\,(acac)_3}$	default	-52	0.0487
	ANN	-5	0.0490
$\mathrm{Cr\,(bipy)_3}$	default	-48	0.0237
	ANN	29	0.0161
$\mathrm{Fe\,(acac)_3}$	default	-50	0.0429
	ANN	23	0.0437
$\mathrm{Mn\,(misc)_3}$	default	-57	0.0459
	ANN	-53	0.0454

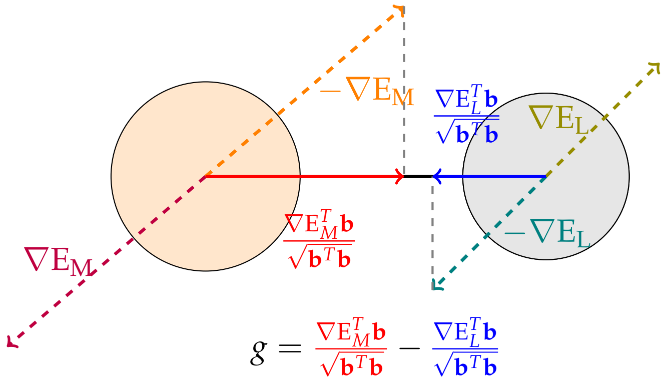

Figure S28: Illustration showing definition of bond projected gradient, g, used to estimate the closeness of an initial geometry to equilibrium. The projected gradient is the scalar difference between the component of the negative energy gradient projected into the vector joining nuvlear positions of the metal (larger orange circle) and the ligand (smaller grey circle).

Table S31: Spin splitting energy predictions and data in kcal/mol, for the CSD test structures. For each complex, values from DFT using B3LYP and and ANN predictions are shown, along with the standard deviation of the ANN model

Test number	ANN	DFT/B3LYP	ANN Std. Dev.	Error
1	21.80	29.06	3.29	-7.81
2	21.90	14.20	3.28	7.70
3	-26.50	-23.90	3.25	-2.66
4	28.70	34.10	3.65	-5.35
5	32.90	35.10	3.46	-2.18
6	-17.00	-11.50	3.87	-5.53
7	12.60	35.2	4.25	-22.60
8	-13.80	-16.40	4.14	2.61
9	30.60	0.26	3.77	30.40
10	-44.50	-40.90	3.27	-3.61
11	-31.20	-15.20	3.74	-16.00
12	4.50	-23.2	5.29	27.70
13	-13.10	-16.70	4.00	3.62
14	28.30	30.70	4.60	-2.38
15	-61.00	-45.10	3.08	-15.9
16	44.40	69.70	5.06	-25.30
17	6.92	3.51	3.92	3.41
18	-19.50	-6.35	3.25	-13.10
19	-11.50	-3.14	4.27	-8.32
20	-21.90	-15.00	3.69	-6.93
21	-5.78	-1.69	3.23	-4.10
22	-11.70	-8.49	3.91	-3.19
23	-4.20	-5.36	3.52	1.16
24	6.44	2.57	3.46	3.87
25	9.44	-0.23	4.73	9.67
26	-7.91	-24.40	4.00	16.40
27	0.54	3.59	3.24	-3.04
28	-13.40	-36.90	5.56	23.50
29	-6.85	-23.90	3.30	17.10
30	-15.60	-17.20	3.76	1.63
31	-28.20	-38.30	3.26	10.10
32	-10.00	-8.98	3.60	-1.03
33	4.50	-23.40	5.29	27.90
34	-22.00	-24.00	3.29	2.01
35	-13.90	-22.40	4.03	8.55

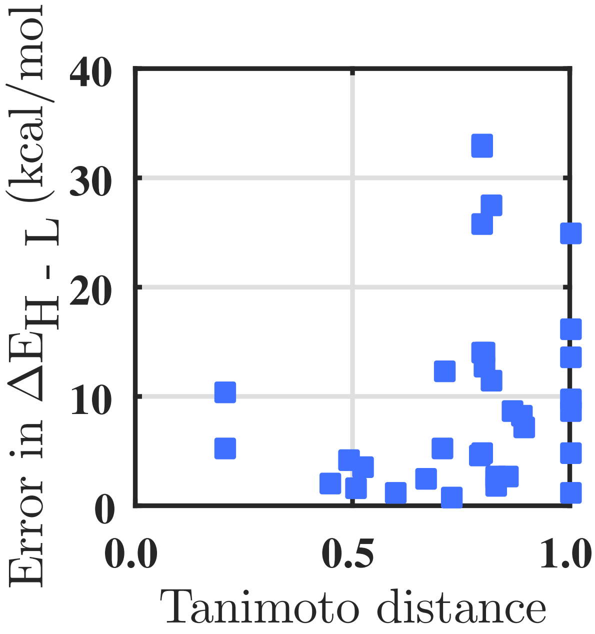

Figure S29: Dissimilarity metrics for CSD data: errors in spin energy predictions for CSD structures are on the y-axis in kcal/mol and the minimum Tanimoto/FP2 disimilarity metric (1 - the Tanimoto index) between the CSD ligands and the training ligands is shown on the x-axis. A value of 1 inidicates no matches with the FP2 fingerprint.

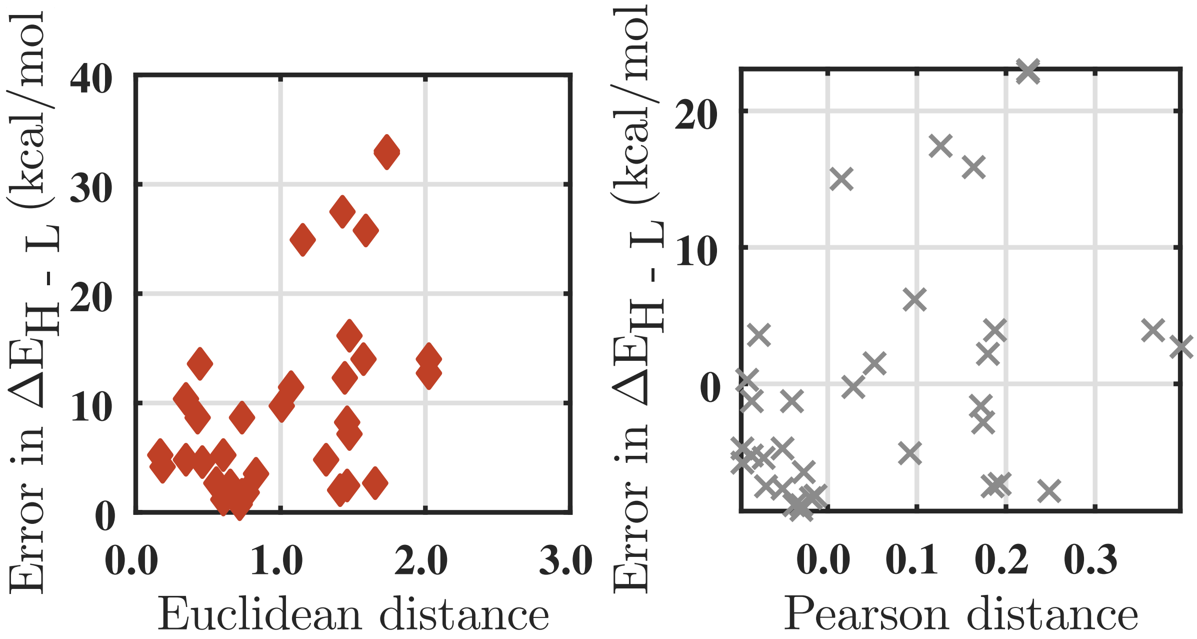

Figure S30: Comparison of dissimilarity metrics for CSD data: errors in spin energy predictions for CSD structures are on the y-axis in kcal/mol and the Euclidean (left, red) and uncentered Pearson distances (gray, right) between the CSD structure and its nearest representation in dimensionless descriptor space is shown on the x-axis.

Table S32: LS bond distance predictions and data in Å, for the CSD test structures. For each complex, values from DFT using B3LYP and and ANN predictions are shown, along with the standard deviation of the ANN model

Test number	ANN	DFT/B3LYP	ANN Std. Dev.	Error
testno	nn	data	variance	error
1	2.01	1.95	0.02	0.06
2	2.01	1.90	0.02	0.11
3	1.97	2.06	0.03	-0.10
4	1.98	1.95	0.03	0.04
5	1.98	2.00	0.03	-0.02
6	1.99	1.97	0.03	0.03
7	1.97	1.96	0.02	0.01
8	2.02	2.07	0.03	-0.05
9	1.99	2.03	0.03	-0.03
10	2.02	2.03	0.03	-0.01
11	1.89	1.99	0.03	-0.10
12	2.03	2.04	0.02	-0.02
13	1.91	1.89	0.03	0.02
14	2.02	1.95	0.03	0.07
15	2.04	2.08	0.03	-0.05
16	1.99	1.91	0.03	0.09
17	2.04	2.01	0.03	0.03
18	1.94	2.00	0.02	-0.06
19	1.90	1.88	0.03	0.02
20	2.08	2.05	0.03	0.03
21	2.08	2.04	0.02	0.03
22	2.05	1.98	0.02	0.07
23	1.94	1.91	0.03	0.03
24	2.05	2.03	0.02	0.02
25	1.98	1.99	0.03	-0.01
26	2.00	1.94	0.03	0.06
27	2.04	1.97	0.02	0.06
28	2.01	2.08	0.02	-0.07
29	1.99	2.04	0.3	-0.05
30	1.88	2.04	0.03	-0.17
31	2.10	2.09	0.02	0.01
32	1.99	2.02	0.03	-0.03
33	2.03	2.04	0.02	-0.01
34	2.08	2.01	0.02	0.07
35	2.16	1.98	0.02	0.19

Table S33: HS bond distance predictions and data in Å, for the CSD test structures. For each complex, values from DFT using B3LYP and and ANN predictions are shown, along with the standard deviation of the ANN model

Test Number	ANN	DFT/B3LYP	ANN Std. Dev.	Error
1	2.06	1.95	0.02	0.11
2	2.06	1.87	0.02	0.16
3	1.94	2.07	0.02	-0.12
4	2.02	2.01	0.02	0.016
5	2.05	2.13	0.02	-0.08
6	2.02	2.06	0.02	-0.04
7	2.06	2.03	0.02	0.03
8	2.09	2.32	0.02	-0.23
9	2.00	2.05	0.02	-0.04
10	2.15	2.12	0.03	0.03
11	1.96	2.03	0.02	-0.07
12	1.98	2.06	0.02	-0.08
13	2.02	1.94	0.02	0.09
14	2.00	2.08	0.02	-0.08
15	2.21	2.22	0.02	-0.01
16	2.07	1.91	0.02	0.16
17	2.06	2.01	0.02	0.06
18	2.14	2.13	0.02	0.01
19	2.02	1.98	0.02	0.04
20	2.11	2.19	0.02	-0.08
21	2.23	2.05	0.02	0.187
22	2.09	2.07	0.02	0.02
23	2.12	2.03	0.02	0.10
24	2.24	2.16	0.02	0.08
25	1.99	2.02	0.02	-0.02
26	2.03	1.96	0.02	0.07
27	2.15	2.17	0.02	-0.02
28	2.13	2.26	0.02	-0.13
29	2.03	2.07	0.02	-0.03
30	2.12	2.08	0.02	0.04
31	2.28	2.25	0.02	0.03
32	2.19	2.22	0.02	-0.03
33	1.98	2.06	0.02	-0.09
34	2.09	2.02	0.02	0.07
35	2.21	1.99	0.02	0.22

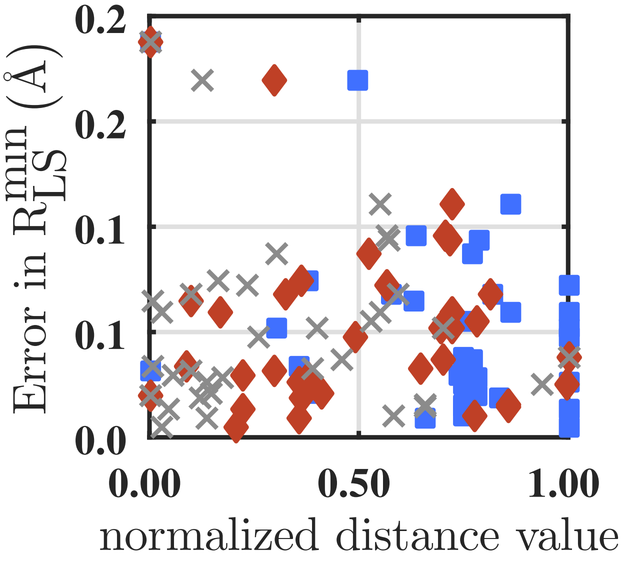

Figure S31: Comparison of dissimilarity metrics for CSD data: errors in LS bond length prediction for CSD structures are shown on the y-axis in Å, and three normalized dissimilarity metrics are compared on the x-axis: the Tanimoto/FP2 disimilarity metric between the CSD ligands and the training ligands (blue circles), and the Euclidean (red diamonds) and uncentered Pearson distances (gray crosses) between the CSD structure and its nearest representation in dimensionless descriptor space.

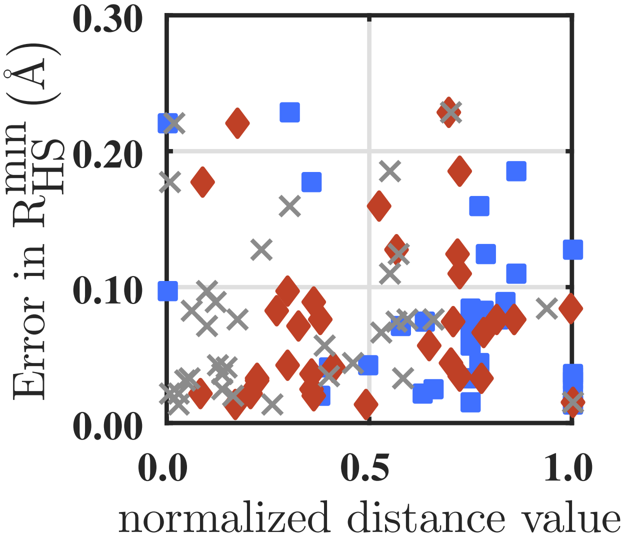

Figure S32: Comparison of dissimilarity metrics for CSD data: errors in HS bond length prediction for CSD structures are shown on the y-axis in Å, and three normalized dissimilarity metrics are compared on the x-axis: the Tanimoto/FP2 disimilarity metric between the CSD ligands and the training ligands (blue circles), and the Euclidean (red diamonds) and uncentered Pearson distances (gray crosses) between the CSD structure and its nearest representation in dimensionless descriptor space.

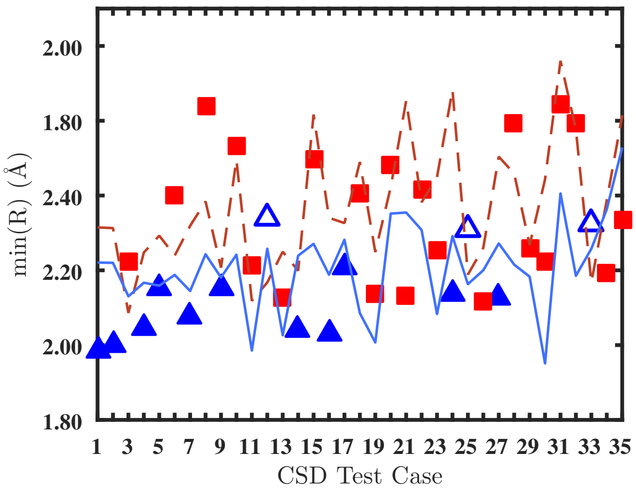

Figure S33: Comparison of measured CSD bond distances in the crystal phase, represented by symbols (red squares for high-spin or blue triangles for low-spin based on DFT assignment at aHF=0.20) with the ANN predicted HS (red line) and LS (blue line) bond distances.

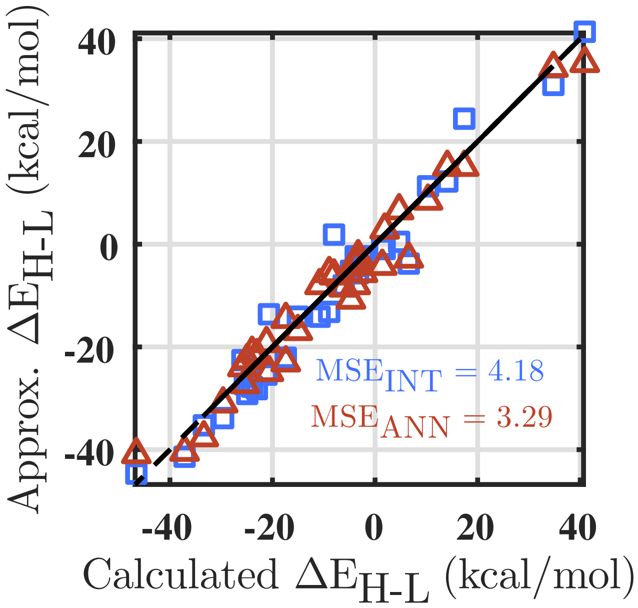

Figure S34: Parity plot comparing prediction of $\Delta E_{HS\text{-}LS}$ in kcal/mol. The x-axis is the DFT value at $a_{HF} = 0.20$, while the y-axis is the predicted value. The blue squares are obtained by interpolating from the values at $a_{HF} = 0.00$ using the predicted slopes. The red triangles are the matching test cases (where these were tested at $a_{HF} = 0.20$).

Table S34: Predicted spin splitting energies in kcal/mol for CSD structures at $a_{HF} = 0.20$ (B3LYP) interpolating data from $a_{HF} = 0$ (GGA) using ANN-predicted HFX sensitivity, compared to data from DFT using B3LYP. The predictions and standard deviations of the ANN model are given in the second and third columns in kcal/mol$\cdot$HFX^{-1}.

Test	ANN $\frac{\partial E_{H-L}}{\partial a_{HF}}$	ANN Std. Dev.	DFT,$a_{HF} = 0.00$	Interpolated	DFT,$a_{HF} = 0.20$	Error
1	-112	16.31	36.54	14.15	29.61	-15.46
2	-113.6	15.90	24.95	2.22	14.18	-11.96
3	-6.95	14.38	-22.58	-23.97	-23.87	-0.10
4	-90.68	15.10	43.97	25.83	34.05	-8.22
5	-85.80	15.01	43.98	26.82	35.05	-8.23
6	-21.31	14.56	-2.01	-6.27	-11.49	5.22
7	-101.30	14.58	37.01	16.75	35.19	-18.44
8	-126.30	13.61	3.26	-21.99	-16.41	-5.58
9	-123.10	14.90	20.23	-4.37	0.26	-4.63
11	-52.19	14.18	-8.39	-18.83	-15.21	-3.62
12	-25.51	14.53	-17.82	-22.92	-23.15	0.23
13	-59.05	14.16	-3.19	-15.00	-16.74	1.75
14	-127.00	15.08	57.46	32.07	30.66	1.41
15	-48.81	14.19	-31.03	-40.80	-45.11	4.32
16	-116.70	14.13	70.10	46.75	69.67	-22.92
17	-95.78	16.33	11.45	-7.70	3.51	-11.21
18	-46.71	13.99	5.40	-3.94	-6.35	2.41
19	-64.62	14.26	12.74	-0.18	-3.14	3.00
20	-83.78	13.97	-3.82	-20.58	-15.01	-5.56
21	-90.32	13.88	17.65	-0.42	-1.69	1.27
22	-105.20	13.67	11.70	-9.34	-8.49	-0.85
23	-80.10	14.00	8.43	-7.59	-5.36	-2.22
24	-62.35	13.85	15.41	2.94	2.57	0.37
25	-72.41	14.40	11.80	-2.69	-0.23	-2.46
26	-16.00	14.40	-21.00	-24.20	-24.36	0.15
27	-115.50	13.71	26.11	3.01	3.59	-0.58
28	-94.16	14.69	-12.56	-31.4	-36.86	5.46
29	-57.17	15.41	-17.22	-28.65	-23.90	-4.75
30	-107.30	13.88	-13.39	-34.85	-17.18	-17.67
31	-79.92	13.64	-23.14	-39.12	-38.34	-0.78
32	-69.41	14.77	2.12	-11.77	-8.98	-2.79
33	-25.51	14.53	-20.30	-25.40	-23.41	-2.00
34	-6.862	14.24	-19.72	-21.09	-23.98	2.89
35	-34.22	13.42	-18.83	-25.68	-22.43	-3.25

.

References

[1] A. A. Aruffo, B. D. Santarsiero, V. Schomaker, and E. C. Lingafelter. Bis(phenylmethanethiolato)(2,3,9,10-tetramethyl-1,4,8,11-tetraaza-1,3,8,10-cyclotetradecatetraene)iron(III) hexafluorophosphate, C28H38FeN4S2+.PF6. *Acta Crystallogr., Sect. C Cryst. Struct. Commun.*, 40(10):1693–1695, 1984. ISSN 01082701. doi: 10.1107/S0108270184009185.

[2] E Bang and O Monsted. *Acta Chem. Scand. A*, page 353, 1982.

[3] AV Capilla, RA Aranda, and F Gomez-Beltran. *Cryst. Struct. Commun*, 9(147), 1980.

[4] Iraklii I. Ebralidze, Gregory Leitus, Linda J.W. Shimon, Yong Wang, Sason Shaik, and Ronny Neumann. Structural Variability in Manganese(II) Complexes of N,N-bis(2-pyridinylmethylene) Ethane (and Propane) Diamine Ligands. *Inorg. Chim. Acta*, 362(13): 4713–4720, 2009. ISSN 00201693. doi: 10.1016/j.ica.2009.06.037.

[5] G.N.Nadzhafov I.R.Amiraslanov K.S.Mamedov E.M.Movsumov F.N.Musaev. X-Raying Investigation into Complexes of n-Aminobenzoic Acid with Metals. *Zh. Strukt. Khim.*, 20: 1075, 1979.

[6] Yarin Gal and Zoubin Ghahramani. Dropout as a Bayesian Approximation: Representing Model Uncertainty in Deep Learning. *arXiv:1506.02142*, 2015.

[7] Burghard Grüning, Gerhard Holze, Titus A. Jenny, Petr Nesvadba, Albert Gossauer, Ludger Ernst, and William S. Sheldrick. Structure and Reactivity of Xanthocorrinoids. Part II. Influence of the c-Acetic-Acid Chain on the Course of the Hydroxylation of the Corrin Chromophore by Oxygen in the Presence of Ascorbic Acid. *Helv. Chim. Acta*, 68(6): 1754–1770, 1985. ISSN 0018019X. doi: 10.1002/hlca.19850680631.

[8] Hong Hai, Hui Wang, Wen Ying Jin, Xiao Dong An, Wen Gang Huang, and Shu Hua Zhang. Synthesis, Crystal Structures, and Properties of Three Complexes of 5-(pyridin-2-ylmethoxy) Isophthalic Acid. *Synth. React. Inorg., Met.-Org., Nano-Met. Chem.*, 45(12): 1870–1874, 2015. ISSN 1553-3174. doi: 10.1080/15533174.2013.867882.

[9] Katja Hansen, Grgoire Montavon, Franziska Biegler, Siamac Fazli, Matthias Rupp, Matthias Scheffler, O. Anatole von Lilienfeld, Alexandre Tkatchenko, and Klaus-Robert Mller. Assessment and validation of machine learning methods for predicting molecular atomization energies. *Journal of Chemical Theory and Computation*, 9(8):3404–3419, 2013. doi: 10.1021/ct400195d. PMID: 26584096.

[10] Øyvind Hatlevik, Atta M. Arif, and Joel S. Miller. Synthesis and Characterization of Hexakis(acetonitrile)chromium(III) tetrafluoroborate, [CrIII(NCMe)6][BF4]3. A Nonaqueous CrIII Source. *J. Phys. Chem. Solids*, 65(1):61–63, 2004. ISSN 00223697. doi: 10.1016/j.jpcs.2003.08.020.

[11] Timo Huxel, Sebastian Riedel, Jochen Lach, and Julia Klingele. Iron(II) and Nickel(II) Complexes of N-Alkylimidazoles and 1-Methyl-1H-1, 2, 4-Triazole: X-ray Studies, Magnetic Characterization, and DFT Calculations. *Z. anorg. allg. Chem*, 638(6):925–934, 2012. ISSN 00442313. doi: 10.1002/zaac.201200117.

[12] Timo Huxel, Serhiy Demeshko, and Julia Klingele. 2-Amino-5-(2-pyridyl)-thiadiazole as Bidentate Ligand. *Z. anorg. allg. Chem.*, 641(10):1711–1717, 2015. ISSN 00442313. doi: 10.1002/zaac.201500144. URL http://doi.wiley.com/10.1002/zaac.201500144.

[13] Efthymios I. Ioannidis, Terry Z. H. Gani, and Heather J. Kulik. molSimplify: A Toolkit for Automating Discovery in Inorganic Chemistry. *J. Comp. Chem.*, 37(22):2106–2117, 2016. ISSN 01928651. doi: 10.1002/jcc.24437.

[14] Zofia Janas, Piotr Sobota, and Tadeusz Lis. Interaction Of Tin Chlorides With Iron, Chromium And Vanadium Chlorides In Tetrahydrofuran. Crystal Structures Of [Fe 2 (-Cl) 3 (thf) 6][SnCl 5 (thf)], [Sn 2 (-OH) 2 Cl 6 (thf) 2]·2thf and trans-[CrCl 2 (thf) 4][SnCl 5 (thf)]. *J. Chem. Soc., Dalt. Trans.*, (9):2429–2434, 1991. ISSN 0300-9246. doi: 10.1039/DT9910002429.

[15] Jan Janczak and Ryszard Kubiak. Stereochemistry And Properties Of The M(II)N(Py) Coordination Bond In The Low-Spin Dipyridinated Iron(II) And Cobalt(II) Phthalocyanines. *Inorg. Chim. Acta*, 342:64–76, 2003. ISSN 00201693. doi: 10.1016/S0020-1693(02)01060-5.

[16] Qing-Qing Liang, Zheng-Yu Liu, En-Cui Yang, and Xiao-Jun Zhao. 1,2,4-Triazole Controlled Cd II /Mn II Complexes with Discrete Mononuclear, Polymeric 1D Chain, and 2D Layer Motifs. *Z. anorg. allg. Chem*, pages NA–NA, 2009. ISSN 00442313. doi: 10.1002/zaac.200900145.

[17] Yan Liu, Duanjun Xu, and Jiagen Liu. Synthesis And Crystal Structure Of Tetraimidazole(Diaqua)-Manganese(II) Terephthalate. *J. Coord. Chem.*, 54(2):175–181, 2001. ISSN 0095-8972. doi: 10.1080/00958970108027153.

[18] Zhen Ma, Manas Sutradhar, Atash V. Gurbanov, Abel M. Maharramov, Rafiga A. Aliyeva, Farqana S. Aliyeva, Fidan N. Bahmanova, Vusala I. Mardanova, Famil M. Chyragov, and Kamran T. Mahmudov. CoIII, NIII and Uo2II Complexes with -Diketones and Their Arylhydrazone Derivatives: Synthesis, Structure and Catalytic Activity in Henry Reaction. *Polyhedron*, 101:14–22, 2015. ISSN 02775387. doi: 10.1016/j.poly.2015.07.054.

[19] Anthony J. Markwell, John M. Pratt, M. Salim Shaikjee, and James G. Toerien. The Chemistry of Vitamin B12. Part 28. Crystal structure of Dicyanocobyrinic Acid Heptamethyl Ester and its Interaction with Alcohols: the Effects of Hydrogen Bonding to Coordinated Cyanide. *J. Chem. Soc., Dalt. Trans.*, (6):1349, 1987. ISSN 0300-9246. doi: 10.1039/dt9870001349.

[20] Dana S. Marlin, Marilyn M. Olmstead, and Pradip K. Mascharak. Reaction of (μ-Oxo)diiron(III) Core with CO_2 in N-Methylimidazole: Formation of Mono(μ-carboxylato)(μ-oxo)diiron(III) Complexes with N-Methylimidazole as Ligands. *Inorg. Chem.*, 42(5):1681–1687, 2003. ISSN 0020-1669. doi: 10.1021/ic0206140. URL http://pubs.acs.org/doi/abs/10.1021/ic0206140.

[21] Toshio Mashiko, Christopher A. Reed, Kenneth J. Haller, Margaret E. Kastner, and W. Robert Scheidt. Thioether Ligation in Iron-Porphyrin Complexes: Models for Cytochrome C. *J. Am. Chem. Soc.*, 103(19):5758–5767, 1981. ISSN 0002-7863. doi: 10.1021/ja00409a024.

[22] Grégoire Montavon, Matthias Rupp, Vivekanand Gobre, Alvaro Vazquez-Mayagoitia, Katja Hansen, Alexandre Tkatchenko, Klaus-Robert Müller, and O Anatole von Lilienfeld. Machine learning of molecular electronic properties in chemical compound space. *New Journal of Physics*, 15(9):095003, 2013. URL http://stacks.iop.org/1367-2630/15/i=9/a=095003.

[23] Susanne Mossin, Henning Osholm Sørensen, and Høgni Weihe. Trans-bis(cyano-κ C)(1,4,8,11-tetraazacyclotetradecane-κ 4 N)manganese(III) Perchlorate, a Low-Spin Manganese(III) Complex. *Acta Crystallogr., Sect. C Cryst. Struct. Commun.*, 58(4):m204–m206, 2002. ISSN 0108-2701. doi: 10.1107/S0108270102001713.

[24] Susanne Mossin, Henning Osholm Sørensen, Høgni Weihe, Jørgen Glerup, and Inger Søtofte. Manganese (III) Cyclam Complexes with Aqua, Iodo, Nitrito, Perchlorato and Acetic Acid/acetato Axial Ligands. *Inorg. Chim. Acta*, 358(4):1096–1106, 2005. ISSN 00201693. doi: 10.1016/j.ica.2004.10.005.

[25] B. Moubaraki, M. Ley, D. Benlian, and J. P. Sorbier. Structure and Single-Crystal Conductivity Measurements of Oxidized 3D Metal Phthalocyanines. Bis-Chloro Derivatives of Chromium(III), Iron(III) and Cobalt(III). *Acta Crystallogr., Sect. C Cryst. Struct. Commun.*, 46(3):379–385, 1990. ISSN 01082701. doi: 10.1107/S0108270189007092.

[26] Emmanuel N. Nfor, Peter F. Asobo, Justin Nenwa, Oswald N. Nfor, Julius N. Njapba, Romanus N. Njong, and Offiong E. Offiong. Nickel (II) and Iron (II) Complexes with Azole Derivatives: Synthesis, Crystal Structures and Antifungal Activities. *Int. J. Inorg. Chem.*, 2013:1–6, 2013. ISSN 2090-2026. doi: 10.1155/2013/987574.

[27] Junichi Nishijo and Masaya Enomoto. Synthesis, Structure And Magnetic Properties Of [Crcyclam(Cc-6-Methoxynaphthalene)2](Tcnq)N(1,2-Dichloroethane) (N=1, 2). *Inorg. Chim. Acta*, 437:59–63, 2015. ISSN 00201693. doi: 10.1016/j.ica.2015.08.007.

[28] Junichi Nishijo, Ken Judai, Shigenori Numao, and Nobuyuki Nishi. Chromium Acetylide Complex Based Ferrimagnet and Weak Ferromagnet. *Inorg. Chem.*, 48(19):9402–9408, 2009. ISSN 0020-1669. doi: 10.1021/ic901295d.

[29] Yukiko Oba and Tomoyuki Mochida. Thermal Properties And Crystal Structures Of Cobalt(III)Cyclam Complexes With The Bis(Trifluoromethanesulfonyl)Amide Anion (Cyclam=1,4,8,11-Tetraazacyclotetradecane). *Polyhedron*, 99:275–279, 2015. ISSN 02775387. doi: 10.1016/j.poly.2015.08.015.

[30] Ranjan Patra, Arvind Chaudhary, Sudip Kumar Ghosh, and Sankar Prasad Rath. Modulation of Metal Displacements in a Saddle Distorted Macrocycle: Synthesis, Structure, and Properties of High-Spin Fe(III) Porphyrins and Implications for the Hemoproteins. *Inorg. Chem.*, 47(18):8324–8335, 2008. ISSN 0020-1669. doi: 10.1021/ic800944q.

[31] C.E. Pfluger and P.S. Haradem. Coordination Sphere Geometry Of Tris(Acetylacetonato)Metal(II) Complexes: The Crystal And Molecular Structure Of Tris(1,1,1,5,5,5-Hexafluoroacetylacetonato)Iron(III). *Inorg. Chim. Acta*, 69:141–146, 1983. ISSN 00201693. doi: 10.1016/S0020-1693(00)83564-1.

[32] Raghunathan Ramakrishnan, Pavlo O. Dral, Matthias Rupp, and O. Anatole von Lilienfeld. Big data meets quantum chemistry approximations: The -machine learning approach. *Journal of Chemical Theory and Computation*, 11(5):2087–2096, 2015. doi: 10.1021/acs.jctc.5b00099. PMID: 26574412.

[33] Hiroshi Sakiyama, Ryoji Mitsuhashi, and Masahiro Mikuriya. Pseudo-S6 Complex Cations of a Hexakis-N-methylformamide Nickel(II) Complex. *X-Ray Struct. Anal. Online*, 31:45–46, 2015. ISSN 1883-3578. doi: 10.2116/xraystruct.31.45.

[34] Fatima Setifi, Akira Ota, Lahcéne Ouahab, Stèphane Golhen, Hideki Yamochi, and Gunzi Saito. Charge Transfer Salts of BO with Paramagnetic Isothiocyanato Complex Anions: (BO)[M(isoq)$_2$(NCS)$_4$]; M=CrIII or FeIII, isoq=isoquinoline and BO=Bis(ethylenedioxo)tetrathiafulvalene. *J. Solid State Chem.*, 168(2):450–456, 2002. ISSN 00224596. doi: 10.1006/jssc.2002.9728. URL http://linkinghub.elsevier.com/retrieve/pii/S0022459602997288.

[35] Y.A. Simonov, A.A. Dvorkin, I.I. Bulgak, M.P. Starish, and D.G. Batir. . *Koord. Khim.*, 5: 1883, 1979.

[36] Thi My Uyen Ton, Ciputra Tejo, Stefani Tania, Joyce Wei Wei Chang, and Philip Wai Hong Chan. Iron(II)-Catalyzed Amidation of Aldehydes with Iminoiodinanes at Room Temperature and under Microwave-Assisted Conditions. *J. Org. Chem.*, 76(12):4894–4904, 2011. ISSN 0022-3263. doi: 10.1021/jo200284a.

[37] Yi-Long Yan, Melissa T. Miller, Yuchen Cao, and Seth M. Cohen. Synthesis of Hydroxypyrone- and Hydroxythiopyrone-Based Matrix Metalloproteinase Inhibitors: Developing a StructureActivity Relationship. *Bioorg. Med. Chem. Lett.*, 19(7):1970–1976, 2009. ISSN 0960894X. doi: 10.1016/j.bmcl.2009.02.044.

[38] Hai-Yan Zhao, Jing-Jun Ma, Xiao-Dong Yang, and Min-Li Yang. Synthesis, Crystal Structure, and Antibacterial Activity of a New Cobalt(II) Complex Containing Imidazole as Ligand. *Synth. React. Inorg., Met.-Org., Nano-Met. Chem.*, 46(1):45–50, 2016. ISSN 1553-3174. doi: 10.1080/15533174.2014.900633.

[39] Xin-Hua Zhao, Shao-Liang Zhang, Dong Shao, and Xin-Yi Wang. Spin Crossover in [Fe(2-Picolylamine) 3] 2+ Adjusted by Organosulfonate Anions. *Inorg. Chem.*, 54(16):7857–7867, 2015. ISSN 0020-1669. doi: 10.1021/acs.inorgchem.5b00870.
